# NLP4NLP+5: The Deep (R)evolution in Speech and Language Processing

**DOI:** 10.3389/frma.2022.863126

**Published:** 2022-07-27

**Authors:** Joseph Mariani, Gil Francopoulo, Patrick Paroubek, Frédéric Vernier

**Affiliations:** ^1^Université Paris-Saclay, CNRS, Laboratoire Interdisciplinaire des Sciences du Numérique, Orsay, France; ^2^Tagmatica, Paris, France

**Keywords:** speech processing, natural language processing, artificial intelligence, neural networks, machine learning, research metrics, text mining

## Abstract

This paper aims at analyzing the changes in the fields of speech and natural language processing over the recent past 5 years (2016–2020). It is in continuation of a series of two papers that we published in 2019 on the analysis of the NLP4NLP corpus, which contained articles published in 34 major conferences and journals in the field of speech and natural language processing, over a period of 50 years (1965–2015), and analyzed with the methods developed in the field of NLP, hence its name. The extended NLP4NLP+5 corpus now covers 55 years, comprising close to 90,000 documents [+30% compared with NLP4NLP: as many articles have been published in the single year 2020 than over the first 25 years (1965–1989)], 67,000 authors (+40%), 590,000 references (+80%), and approximately 380 million words (+40%). These analyses are conducted globally or comparatively among sources and also with the general scientific literature, with a focus on the past 5 years. It concludes in identifying profound changes in research topics as well as in the emergence of a new generation of authors and the appearance of new publications around artificial intelligence, neural networks, machine learning, and word embedding.

## Introduction

### Preliminary Remarks

The global aim of this series of studies was to investigate the speech and natural language processing (SNLP), research area through the related scientific publications, using a set of NLP tools, in harmony with the growing interest for scientometrics in SNLP [refer to Banchs, 2012; Jurafsky, 2016; Atanassova et al., 2019; Goh and Lepage, 2019; Mohammad, 2020a,b,c; Wang et al., 2020; Sharma et al., 2021 and many more] or in various domains such as economics (Muñoz-Céspedes et al., 2021), finance (Daudert and Ahmadi, 2019), or disinformation (Monogarova et al., 2021).

The first results of these studies were presented in two companion papers, published in the first special issue “Mining Scientific Papers Volume I: NLP-enhanced Bibliometrics” of the *Frontiers in Research Metrics and Analytics* journal; one on production, collaboration, and citation: “The NLP4NLP Corpus (I): 50 Years of Publication, Collaboration and Citation in Speech and Language Processing” (Mariani et al., 2019a) and a second one on the evolution of research topics over time, innovation, use of language resources and reuse of papers and plagiarism within and across publications: “The NLP4NLP Corpus (II): 50 Years of Research in Speech and Language Processing” (Mariani et al., 2019b).

We now extend this corpus by considering the articles published in the same 34 sources over the past 5 years (2016–2020). We watched during this period an increasing interest for machine-learning approaches for processing speech and natural language, and we wanted to examine how this was reflected in the scientific literature. Here, we therefore analyze these augmented data to identify the changes that happened during this period, both in terms of scientific topics and in terms of research community, reporting the results of this new study in a single article covering papers and authors' production and citation within these sources, which is submitted to the second special issue “Mining Scientific Papers Volume II: Knowledge Discovery and Data Exploitation” of the *Frontiers in Research Metrics and Analytics* journal. We invite the reader to refer to the previous extensive articles to get more insights on the used data and developed methods. In addition, we conducted here the study of the more than 1 million total number of references, to measure the possible influence of neighboring disciplines outside the NLP4NLP sources.

### The NLP4NLP Speech and Natural Language Processing Corpus

The NLP4NLP corpus^1^ (Mariani et al., 2019a) contained papers from 34 conferences and journals on natural language processing (NLP) and spoken language processing (SLP) (Table 1) published over 50 years (1965–2015), gathering about 68,000 articles and 270MWords from about 50,000 different authors, and about 325,000 references. Although it represents a good picture of the international research investigations of the SNLP community, many papers, including important seminal papers, related to this field, may have been published in other publications than these. Given the uncertainty of the existence of a proper review process, we did not include the content neither of workshops nor of publications such as *arXiv*^2^, a popular non-peer-reviewed, free distribution service and open-access archive created in 1991 and now maintained at Cornell University. It should be noticed that conferences may be held annually, may appear every 2 years (on even or odd years), and may also be organized jointly on the same year.

**Table 1:** The NLP4NLP+5 corpus of conferences (24) and journals (10).

**Short** ** name**	**#** ** docs**	**Format**	**Long name**	**Language**	**Access to** ** content**	**Period**	**#** ** events**
acl	6,713	Conference	Association for Computational Linguistics conference series	English	Open access^*^	1979–2020	42
acmtslp	82	Journal	ACM Transaction on Speech and Language Processing	English	Private access	2004–2013	10
alta	361	Conference	Australasian Language Technology Association conference series	English	Open access^*^	2003–2019	17
anlp	278	Conference	Applied Natural Language Processing	English	Open access^*^	1983–2000	6
cath	927	Journal	Computers and the Humanities	English	Private access	1966–2004	39
cl	905	Journal	American Journal of Computational Linguistics	English	Open access^*^	1980–2020	41
coling	5,091	Conference	Conference on Computational Linguistics	English	Open access^*^	1965–2020	24
conll	1,124	Conference	Computational Natural Language Learning	English	Open access^*^	1997–2020	23
csal	1,111	Journal	Computer Speech and Language	English	Private access	1986–2020	34
eacl	1,139	Conference	European Chapter of the ACL conference series	English	Open access^*^	1983–2017	15
emnlp	4,588	Conference	Empirical methods in natural language processing	English	Open access^*^	1996–2020	25
hlt	2,219	Conference	Human Language Technology	English	Open access^*^	1986–2015	19
icassps	10,971	Conference	IEEE International Conference on Acoustics, Speech and Signal Processing - Speech Track	English	Private access	1990–2020	31
ijcnlp	2,047	Conference	International Joint Conference on NLP	English	Open access^*^	2005–2019	8
inlg	495	Conference	International Conference on Natural Language Generation	English	Open access^*^	1996–2020	12
isca	22,778	Conference	International Speech Communication Association conference series	English	Open access	1987–2020	33
jep	739	Conference	Journées d'Etudes sur la Parole	French	Open access^*^	2002–2020	8
lre	490	Journal	Language Resources and Evaluation	English	Private access	2005–2020	16
lrec	6,920	Conference	Language Resources and Evaluation Conference	English	Open access^*^	1998–2020	12
ltc	793	Conference	Language and Technology Conference	English	Private access	1995–2019	9
modulad	232	Journal	Le Monde des Utilisateurs de L'Analyse des Données	French	Open access	1988–2010	23
mts	906	Conference	Machine Translation Summit	English	Open access^*^	1987–2019	17
muc	149	Conference	Message Understanding Conference	English	Open access^*^	1991–1998	5
naacl	2,175	Conference	North American Chapter of the ACL conference series	English	Open access^*^	2000–2019	14
paclic	1,352	Conference	Pacific Asia Conference on Language, Information and Computation	English	Open access^*^	1995–2018	23
ranlp	521	Conference	Recent Advances in Natural Language Processing	English	Open access^*^	2009–2019	4
sem	1,089	Conference	Lexical and Computational Semantics / Semantic Evaluation	English	Open access^*^	2001–2020	13
speechc	1,087	Journal	Speech Communication	English	Private access	1982–2020	39
tacl	307	Journal	Transactions of the Association for Computational Linguistics	English	Open access^*^	2013–2020	8
tal	222	Journal	Revue Traitement Automatique du Langage	French	Open access	2006–2020	15
taln	1,250	Conference	Traitement Automatique du Langage Naturel	French	Open access^*^	1997–2020	24
taslp	7,387	Journal	IEEE/ACM Transactions on Audio, Speech, and Language Processing	English	Private access	1975–2020	46
tipster	105	Conference	Tipster Defense Advanced Research Projects Agency (DARPA) text program	English	Open access^*^	1993–1998	3
trec	2,199	Conference	Text Retrieval Conference	English	Open access	1992–2020	29
Total incl. duplicates	88,752					1965–2020	687
Total excl. duplicates	85,138					1965–2020	667

* *Included in the ACL anthology*.

### The NLP4NLP+5 Speech and Natural Language Processing Corpus

The NLP4NLP+5 corpus covers the same 34 publications up to 2020, hence 5 more years (2016–2020), which represents an addition in time of 10%. We preferred not to add new sources to facilitate the comparison between the situations in 2015 and 2020. However, we added in the present paper a Section Analysis of the Citation in NLP4NLP Papers of Sources From the Scientific Literature Outside NLP4NLP on the study of references to papers published in other sources than those of NLP4NLP. This new corpus also includes some articles published in 2015, which were not yet available at the time we produced the first NLP4NLP corpus. Some publications may no longer exist in this extended period (Table 1).

The extended NLP4NLP+5 new corpus contains 88,752 papers [+20,815 papers (+30%) compared with NLP4NLP], 85,138 papers if we exclude duplicates (such as papers published at joint conferences) and 84,006 papers after content checking (+20,649 papers), 587,000 references [+262,578 references (+80%)], 381 MWords [+111 MWords (+40%)], and 66,995 authors [+18,101 authors (+40%)]. The large increase in these numbers illustrates the dynamics of this research field.

To study the possible differences across different communities, we considered two different research areas, speech processing and natural language processing, and we attached the sources to each of those areas (Table 2), given that some sources (e.g., LREC, LRE, L&TC, MTS) may be attached to both research domains. We see that the number of documents related to speech is larger than the one related to NLP. We only considered the papers related to speech processing (named ICASSPS) in the IEEE ICASSP conference, which also includes a large number of papers on acoustics and signal processing in general.

**Table 2:** Sources attached to each of the two research areas.

**Research area**	**Sources**	**# Docs**
NLP-oriented	acl, alta, anlp, cath, cl, coling, conll, eacl, emnlp, hlt, ijcnlp, inlg, lre, lrec, ltc, mts, muc, naacl, paclic, ranlp, sem, tacl, tal, taln, tipster, trec	40,751
Speech-oriented	acmtslp, csal, icassps, isca, jep, lre, lrec, ltc, mts, speechc, taslp	53,264

The number of conference or journal events^3^ may largely vary, from 3 for Tipster to 46 for the *Institute of Electrical and Electronics Engineers (IEEE)/Association for Computing Machiner (ACM) TASLP* and the time span is also different, from 5 years for Tipster to 55 years for COLING. The number of papers in each source largely varies, from 82 papers for the *ACM TSLP* to 22,778 papers for the ISCA conference series.

## Global Analysis Of The Conferences And Journals

### Production of Papers Over the Years

**Figure 1:**
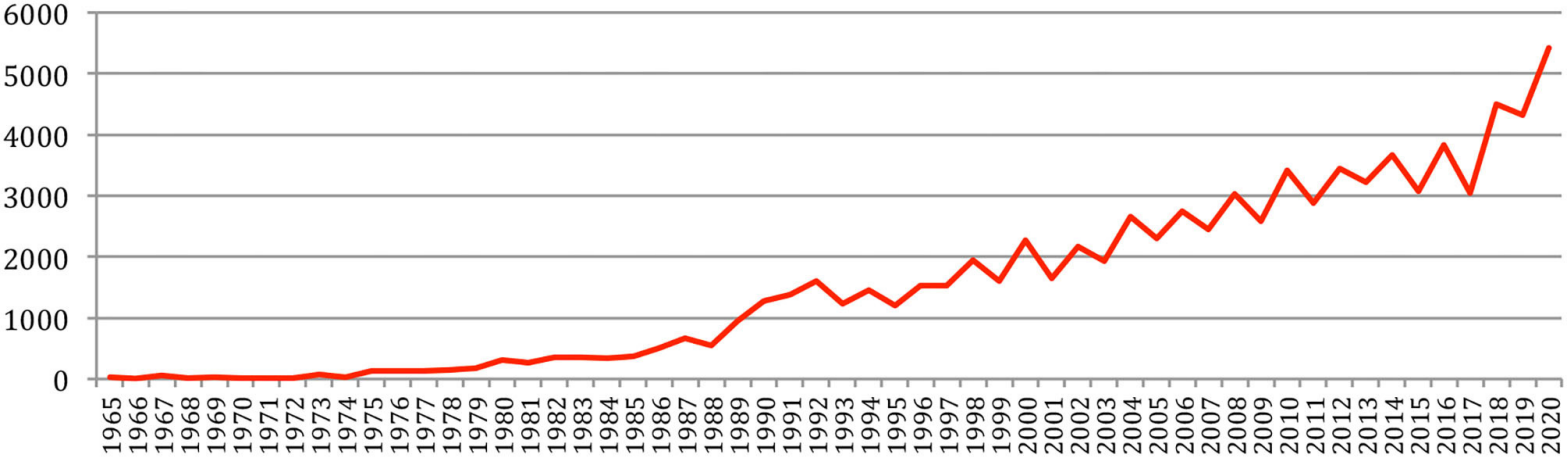
Number of papers each year.

A total number of 88,752 documents have been published in the 34 sources over the years. If we do not separate the papers that were published at joint conferences, it reduces to 85,138 papers (Table 1), with a steady increase over time from 24 papers in 1965 to 5,430 in 2020 (Figure 1). This number fluctuates over the years, mainly due to the biennial frequency of some important conferences (especially LREC and COLING on even-numbered years). The largest number of papers ever has been published in 2020 (5,430 papers), comparable in a single year to the total number of papers (5,725 papers) published over the 25 initial years (1965–1989)!

**Figure 2:**
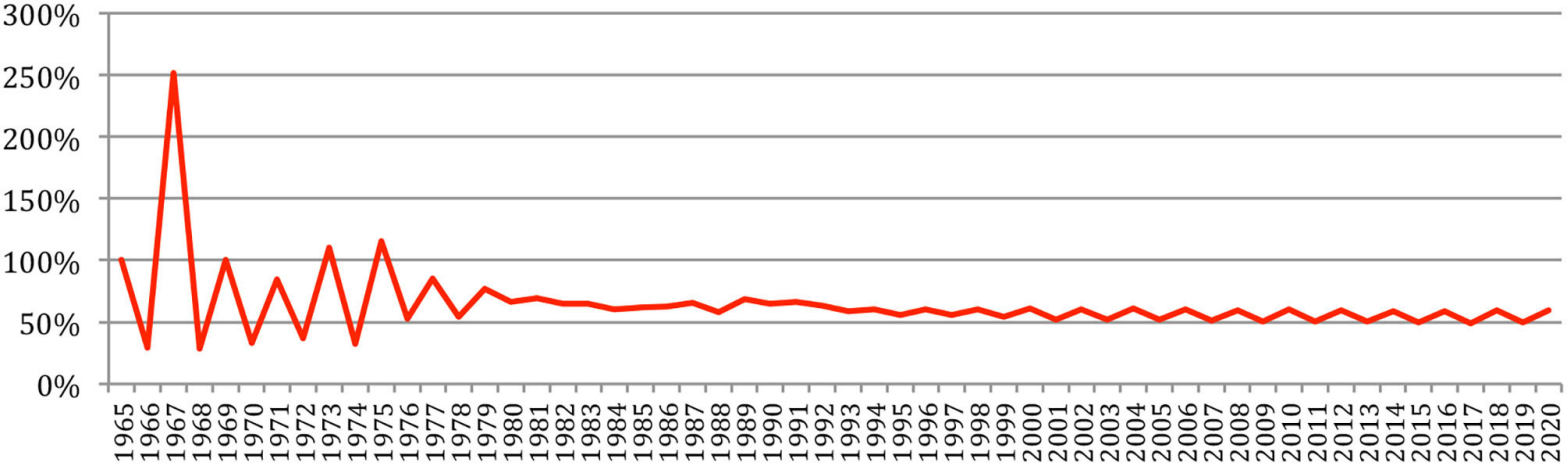
Increase in the number of papers over the years.

**Figure 3:**
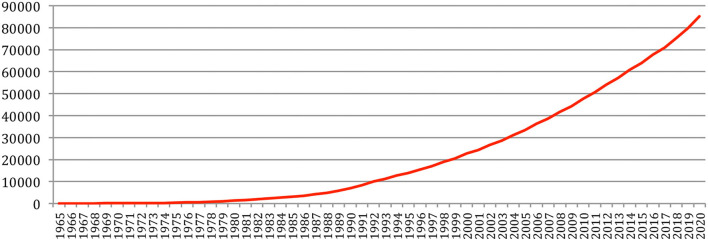
Cumulated number of papers over the years.

The total number of papers itself still increases steadily at a rate which now stabilizes at about 6% per year (Figure 2), reaching 85,138 different documents as of 2020 (Figure 3).

### Data and Tools

Most of the data are freely available online on the Association for Computational Linguistics (ACL) anthology website, and others are freely available in the International Speech Communication Association (ISCA) and Text Retrieval Conference (TREC) archives. IEEE International Conference on Acoustics, Speech and Signal Processing - Speech Track (ICASSP) and Transactions on Audio, Speech, and Language Processing (TASLP) articles have been obtained through the IEEE, and Language Resources and Evaluation (LRE) articles through Springer. For this study, we only considered the papers written in English and French. Most of the documents were available as textual data in PDF, whereas the eldest ones were only available as scanned images and had to be OCRized, which resulted in a lower quality. The study of the authors, of the papers as well as of the papers cited in the references, is problematic due to variations in the same name (family name and given name, initials, middle initials, ordering, married name, etc.) and required a very tedious semi-automatic cleaning process (Mariani et al., 2014), and the same for the sources cited in the references. After a preprocessing phase, the metadata and contents are processed by higher level NLP tools, including a series of Java programs that we developed (Francopoulo et al., 2015a,b, 2016a).

### Overall Analysis

#### Authors' Renewal and Redundancy

**Figure 4:**
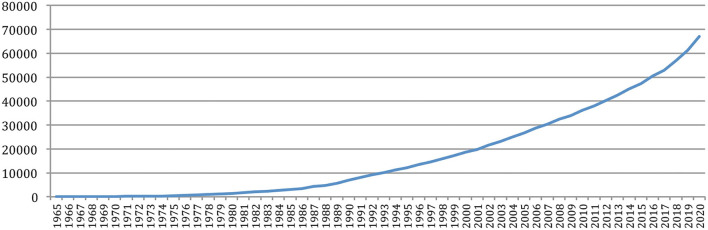
Number of different authors over the years.

We studied the authors' renewal. Figure 4 clearly shows that the number of different authors increased a lot over the years, and especially in the recent years, in a similar way than the number of papers, to reach 66,995 authors in 2020.

**Figure 5:**
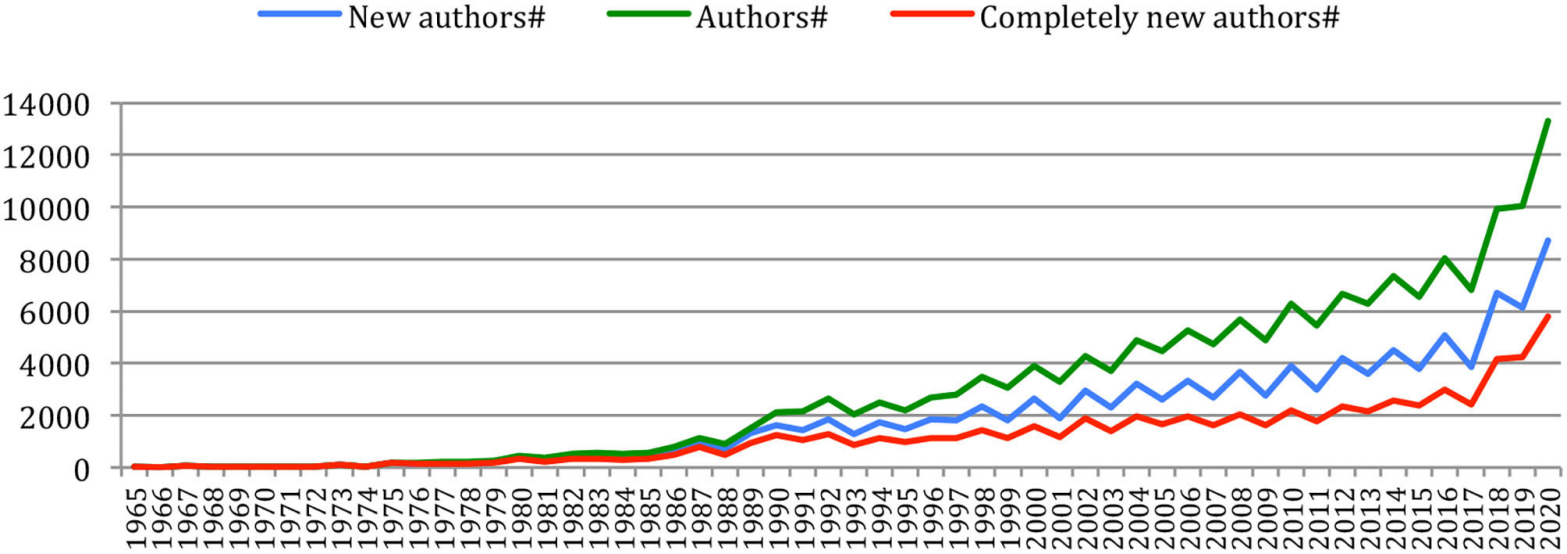
Number of different authors, new authors, and completely new authors over time.

The number of different authors on a year also globally increased over time (Figure 5), with an exceptional increase in the past 5 years (from 6,562 in 2015 to 13,299 in 2020). The number of *new authors* from one conference to the next similarly increased over time, as well as the number of *completely new authors*, who had never published at any previous conference or journal issue. The largest number of *completely new authors* was in 2020 (5,778 authors), comparable in a single year to the total number of different authors (5,688) who published over the 25 initial years (1965–1989)!

**Figure 6:**
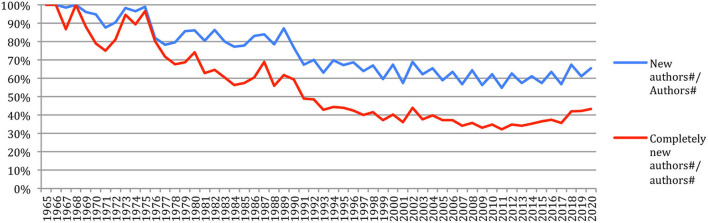
Percentage of new authors and completely new authors over time.

The percentage of *new authors* (Figure 6), which decreased from 100% in 1966 to 55% in 2011, increased since then to reach 65% in 2020, while the percentage of *completely new authors*, which decreased from 100% in 1966 to about 32% in 2011, now increased since then to reach 43% in 2020. This may reflect the arrival of “new blood” in the field, as it will be reflected in the next sections related to the analysis of authors' production, collaboration and citation, and the fact that researchers who started their careers in their 20s in 1965, which corresponds to the first year considered in our study, are now gradually retiring in their 70s.

**Figure 7:**
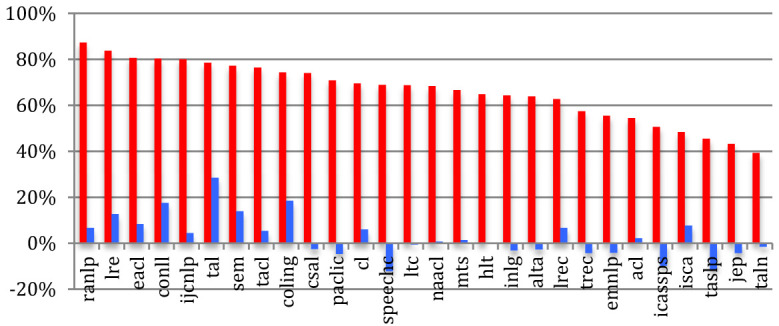
Percentage of completely new authors in the most recent event across the sources in 2020 (red) and difference with 2015 (blue).

If we compare sources, the percentage of *completely new authors* at the most recent event of conferences or journals within the past 5 years (Figure 7) ranges from 39% for TALN or 43% for the JEP to 87% for RANLP or 81% for EACL, while the largest conferences show relatively low percentages (48% for ISCA, 51% for IEEE ICASSPS, 55% for ACL, and 56% for EMNLP). Compared with 2015, we notice a global increase in the percentage of completely new authors, especially in conferences and journals related to NLP.

**Figure 8:**
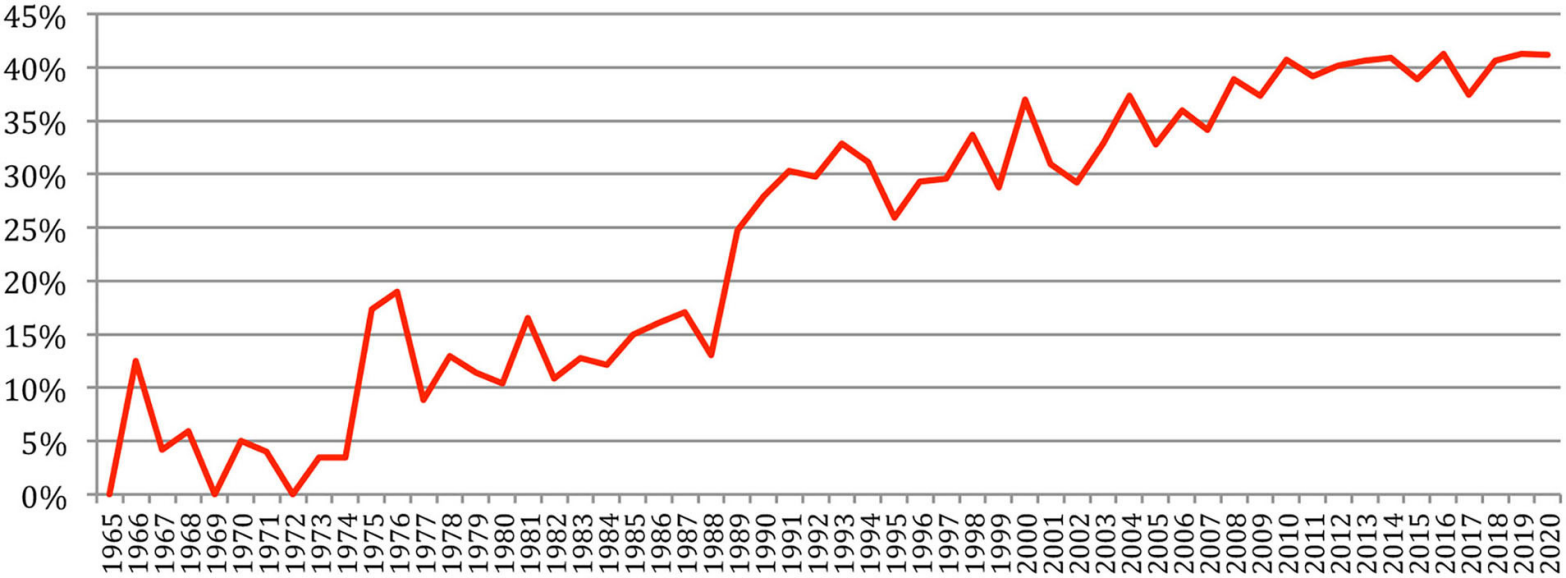
Author redundancy over time.

We defined the *author variety* as the ratio of the number of different authors to the number of authorships^4^ at each conference. This ratio would be 100% if each author's name appears in only one paper. *Author redundancy* corresponds to 100% *author variety*. *Author redundancy* increased over time and has now stabilized at about 40% since 2008 (Figure 8).

**Figure 9:**
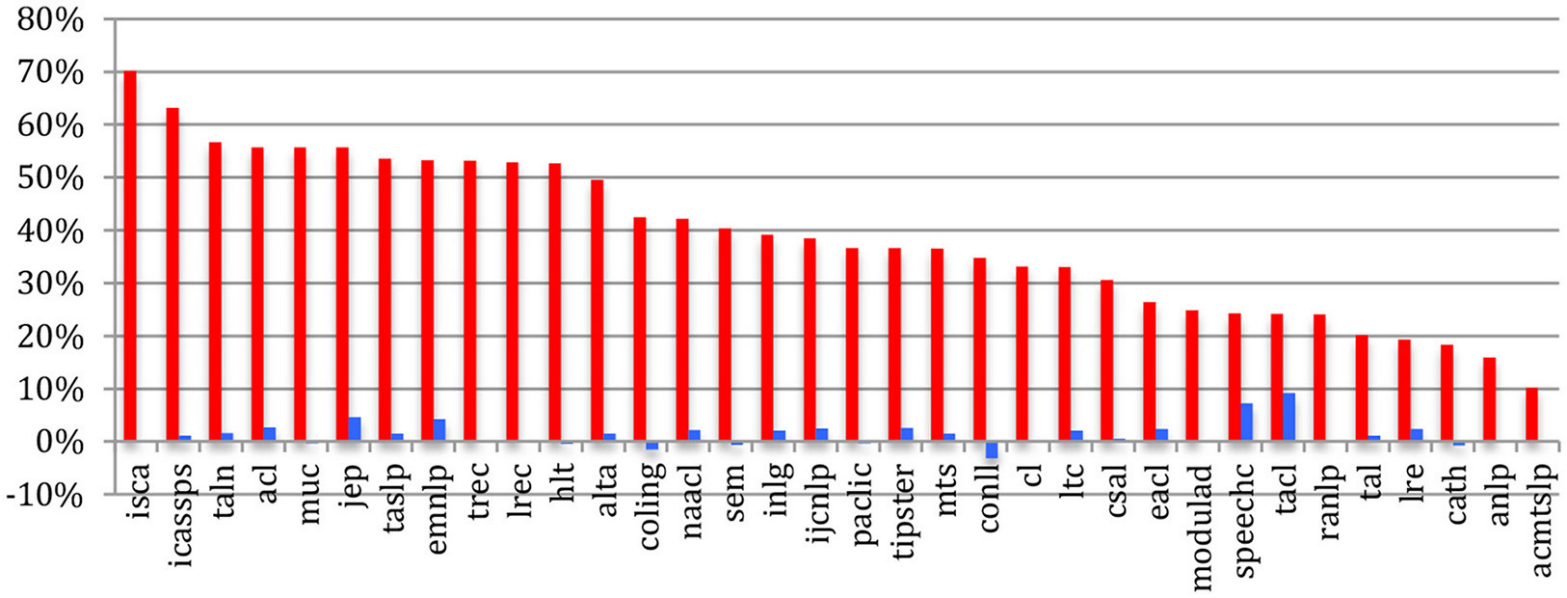
Author redundancy across the sources in 2020 (red) and difference with 2015 (blue).

*Author redundancy* is large in conferences such as ISCA or ICASSP, whereas it is lower in journals and slightly increased globally since 2015 (Figure 9).

#### Papers and Authorship

**Figure 10:**
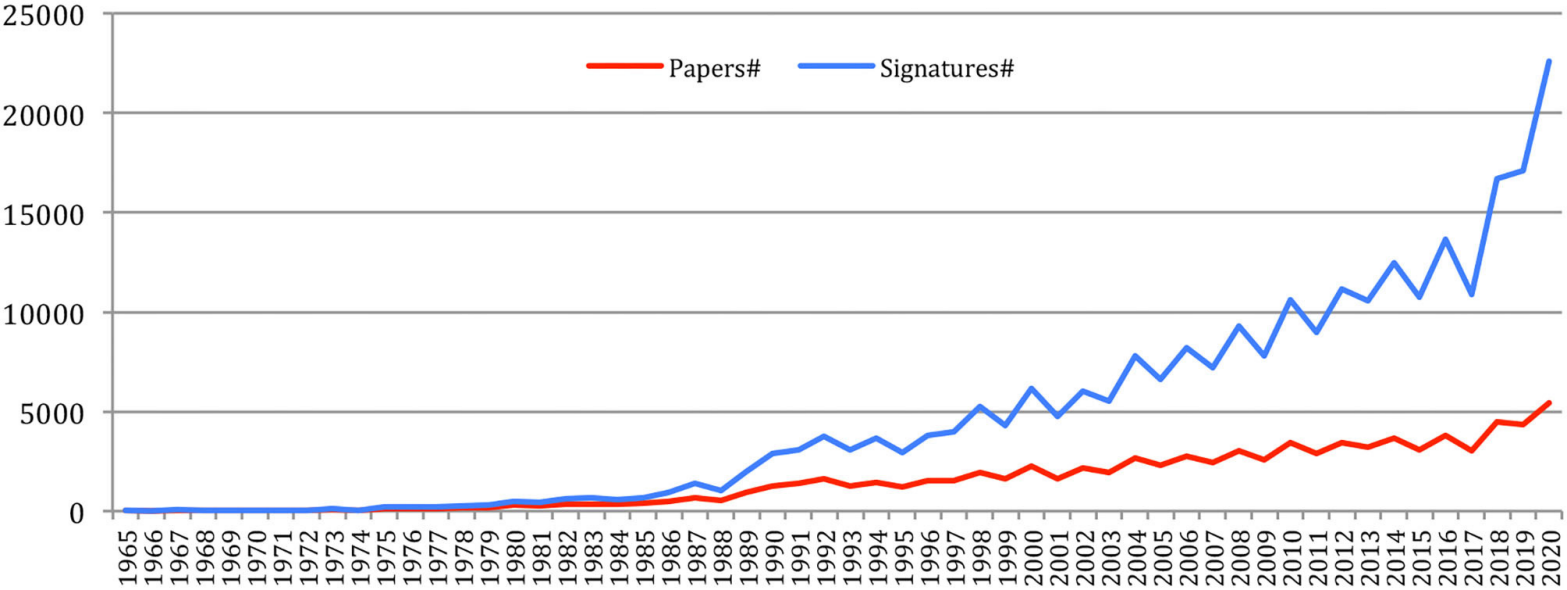
Number of papers and authorships over time.

The number of authorships increases from 32 in 1965 to 22,610 in 2020 at even a higher pace than the number of papers (Figure 10).

#### Authors' Gender

The author gender study is performed with the help of a lexicon of given names with gender information (male, female, epicene^5^). As already noted, variations due to different cultural habits for naming people (single vs. multiple given names, family vs. clan names, inclusion of honorific particles, ordering of the components, etc.) (Fu et al., 2010), changes in editorial practices, and sharing of the same name by large groups of individuals contribute to make identification by name a real issue (Vogel and Jurafsky, 2012). In some cases, we only have an initial for the first name, which made gender guessing impossible unless the same person appears with his/her first name in full in another publication. Although the result of the automatic processing was hand-checked by an expert of the domain for the most frequent names, the results presented here should therefore be considered with caution, allowing for an error margin.

**Figure 11:**
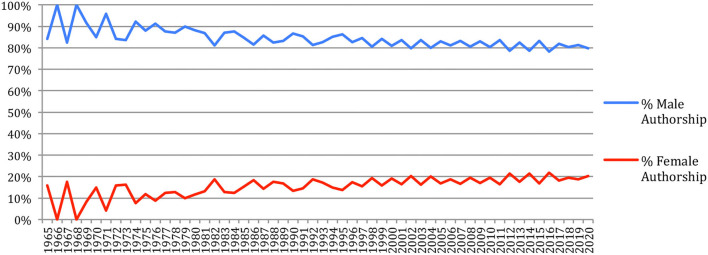
Gender of the authors' contributions over time.

A total of 46% of the authors are male, whereas 14% are female, 4% are epicene, and 36% are of unknown gender. Considering the paper authorships, which take into account the authors' productivity, and assuming that the authors of unknown gender have the same gender distribution as the ones that are categorized, male authors account in 2020 for 80% (compared to 83% in 2015) and female authors for 20% (compared to 17% in 2015) of the authorships (Figure 11), hence a slight improvement.

**Figure 12:**
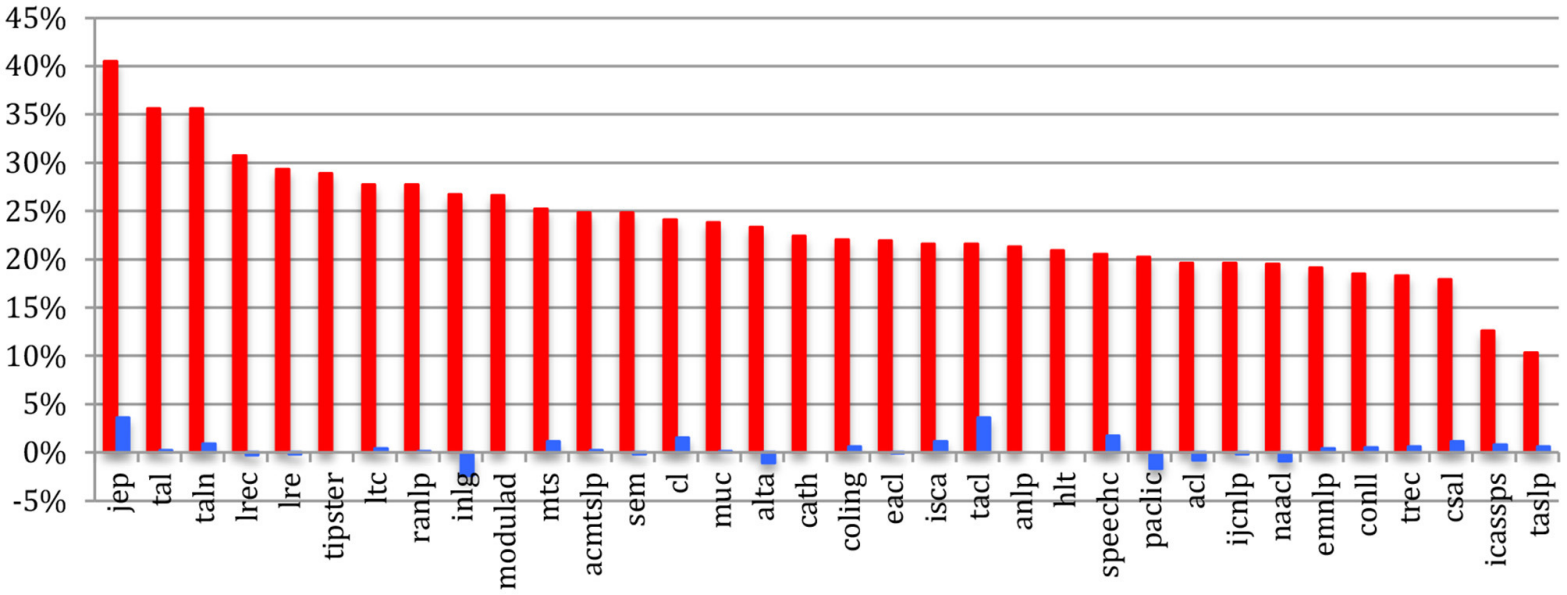
Percentage of female authors across the sources in 2020 (red) and difference with 2015 (blue).

IEEE *TASLP* and ICASSPS have, in 2020 just as in 2015, the most unbalanced situation (respectively, 10 and 13% of female authors), whereas the French conferences (JEP, TALN) and journals (TAL), together with LRE and LREC, have a better balanced one (from 30 to 41% of female authors). The largest increase over the past 5 years (+4%) appears for JEP and TACL (Figure 12).

#### Authors' Production and Co-production

The most productive author published 453 papers, whereas 36,791 authors (55% of the 66,995 authors) published only one paper. Table 3 gives the list of the 12 most productive authors. We see that the eight most productive authors are the same than in 2015, with a slightly different ranking. A total of two newcomers are ranked 9 and 10, specialized in machine learning (ML): James R. Glass (unsupervised ML) and Yang Liu (Federated ML). Some authors (James Glass, Yang Liu, Haizhou Li, Satoshi Nakamura, and Shri Narayanan) increased their number of papers by 30% and more within the past 5 years!

**Table 3:** 12 most productive authors (up to 2020, in comparison with 2015).

**Rank**	**Name**	**#papers**	**Previous**	**Previous**	**Delta**	**Delta%**
**2020**			**Rank**	**#Papers**		
			**2015**			
1	Shrikanth S. Narayanan	453	1	358	95	27%
2	Hermann Ney	388	2	343	45	13%
3	John H. L. Hansen	354	3	299	55	18%
4	Haizhou Li	350	4	257	93	36%
5	Satoshi Nakamura	263	7	205	58	28%
6	Chin Hui P. Lee	261	5	218	43	20%
7	Alex Waibel	234	6	207	27	13%
8	Mark J. F. Gales	230	8	195	35	18%
9	James R. Glass	214	25	142	72	51%
10	Yang Liu	209	19	148	61	41%
11	Lin Shan Lee	204	9	193	11	6%
12	Li Deng	201	10	192	9	5%

But if we focus on the past 5 years (2016–2020) (Table 4), we notice that only two authors (Shrikanth S. Narayanan and Haizhou Li)^6^ still appear in that ranking. Others energically contributed to the research effort on speech and language processing with a new angle benefiting from supervised or unsupervised machine-learning approaches, some already active in that field but also many new names, showing the great vitality of a new generation of researchers, who published more than 15 papers per year in this recent 5-year period.

**Table 4:** 12 most productive authors in the past 5 years (2016 to 2020).

**Rank**	**Name**	**#papers**
1	Graham Neubig	109
2	Shrikanth S. Narayanan	103
3	Haizhou Li	100
4	Yue Zhang	99
5	Björn W. Schuller	91
6	Dong Yu	83
7	Iryna Gurevych	80
8	Junichi Yamagishi	80
9	Shinji Watanabe	78
10	James R. Glass	77
11	Helen M. Meng	72
12	Pushpak Bhattacharyya	71

### Collaborations

#### Authors' Collaborations

**Figure 13:**
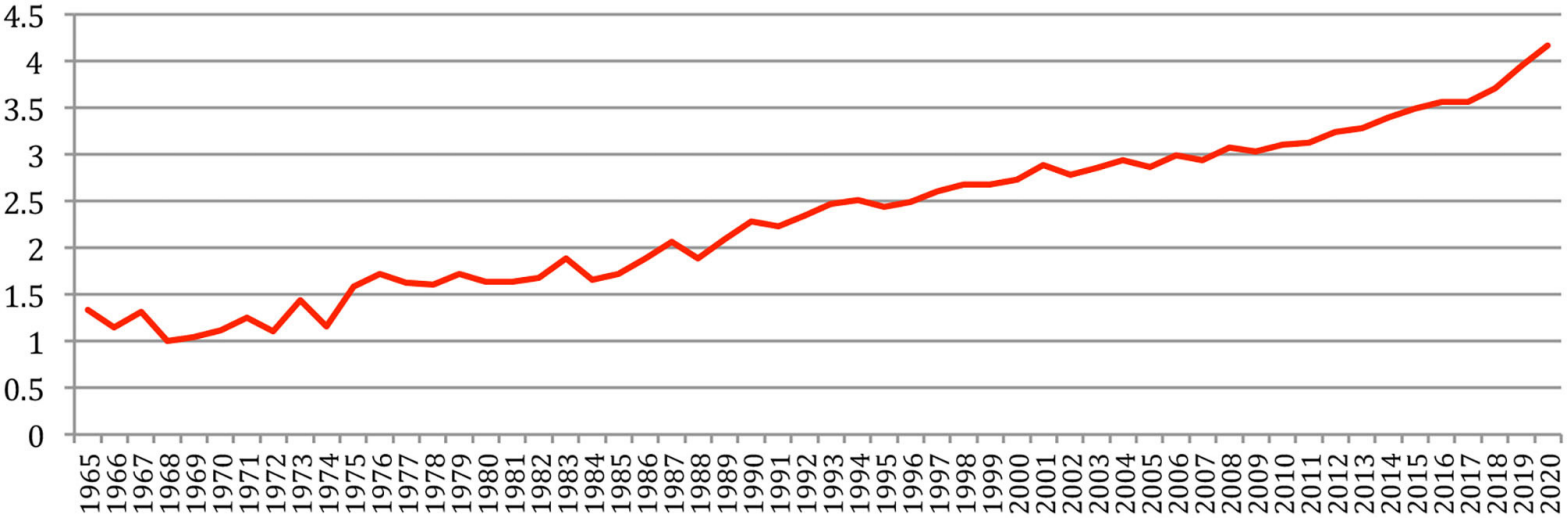
Average number of authors per paper.

The number of authors per paper still increases, with more than 4 authors per paper on average, compared with 3.5 in 2015 (Figure 13).

Table 5 gives the number of authors who published papers as single authors, and the names of the ones who published 10 papers or more. A total of 60,193 authors (90% of the authors) never published a paper as single author^7^. The ranking is very similar to 2015, including six newcomers (Mark Huckvale, Mark Jan Nederhof, Hagai Aronowitz, Philip Rose, Shunichi Ishihara, and Oi Yee Kwong).

**Table 5:** Number and names of authors of single author papers.

**#Papers**	**#Authors**	**Author name**
28	1	W. Nick Campbell
26	1	Jerome R. Bellegarda
24	2	Ellen M. Voorhees, Olivier Ferret
21	1	Ralph Grishman
20	1	Takayuki Arai
18	2	Mark A. Johnson, Rathinavelu Chengalvarayan
17	3	Beth M. Sundheim, Douglas B. Paul, Kenneth C. Litkowski
16	3	Jerry R. Hobbs, Oi Yee Kwong, Steven M. Kay
15	1	Donna Harman
14	4	Dominique Desbois, John Makhoul, Patrick Saint-Dizier, Sadaoki Furui
13	4	Eckhard Bick, Paul S. Jacobs, Rens Bod, Robert C. Moore
12	11	David S. Pallett, Harvey F. Silverman, Jen Tzung Chien, Jörg Tiedemann, Lynette Hirschman, Marius A. Pasca, Martin Kay, Reinhard Rapp, Stephen Tomlinson, Ted Pedersen, Yorick Wilks
11	10	Dekang Lin, Eduard H. Hovy, Hagai Aronowitz, Michael Schiehlen, Philip Rose, Philippe Blache, Roger K. Moore, Shunichi Ishihara, Stephanie Seneff, Tomek Strzalkowski
10	11	Aravind K. Joshi, Hermann Ney, Hugo Van Hamme, Joshua T. Goodman, Karen Spärck Jones, Kenneth Ward Church, Kuldip K. Paliwal, Mark Hepple, Mark A. Huckvale, Mark Jan Nederhof, Olov Engwall
9	31	…
8	25	…
7	51	…
6	90	…
5	124	…
4	224	…
3	447	…
2	1,088	…
1	4,667	…
0	60,193	…

The number of papers with a single author decreased from 75% in 1965 to 3% in 2020, illustrating the changes in the way research that is being conducted.

Up to 2015, the paper with the largest number of co-authors was a META-Net paper published at LREC 2014 (44 co-authors). It is now surpassed by three other papers:

- A paper with 45 co-authors from Microsoft published in TACL 2020
- A paper with 47 co-authors on the European Language Technology landscape published at LREC 2020
- A paper with 58 co-authors on the I4U Speaker Recognition NIST evaluation 2016 published at Interspeech 2017.

The most collaborating author collaborated with 403 different co-authors, whereas 2,430 authors only published alone. An author collaborates on average with 7.9 other authors (compared to 6.6 in 2015), whereas 157 authors published with 100 or more different co-authors. Table 6 provides the list of the 12 authors with the highest number of co-authors.

**Table 6:** The 12 authors with the largest number of co-authors (up to 2020, in comparison with 2015).

**Name**	**#Co-**	**Rank**	**Previous**	**Previous**	**New**
	**authors**	**2020**	**rank**	**#co-**	**co-authors**
			**2015**	**authors**	**2015–2020**
Shrikanth S. Narayanan	403	1	1	299	104
Haizhou Li	355	2	3	252	103
Satoshi Nakamura	292	3	4	234	58
Björn W. Schuller	291	4	39	135	156
Yang Liu	290	5	12	178	112
Hermann Ney	288	6	2	254	34
Sanjeev Khudanpur	284	7	8	193	91
Khalid Choukri	253	8	15	177	76
Ming Zhou	246	9	71	115	131
Chin Hui P. Lee	241	10	7	194	47
Dong Yu	241	10	187	82	159
Alan W. Black	238	12	25	149	89

Table 7 provides the list of the 12 authors who had the largest number of collaborations, possibly with the same co-authors.

**Table 7:** The 12 authors with the largest number of collaborations (up to 2020, in comparison with 2015).

**Name**	**#Collaborations**	**Rank**	**Previous**	**#Collaborations**	**New**
	**2020**	**2020**	**rank 2015**	**2015**	**collaborations 2015–2020**
Shrikanth S. Narayanan	1,411	1	1	1,035	376
Haizhou Li	1,288	2	2	899	389
Hermann Ney	1,026	3	3	890	136
Satoshi Nakamura	861	4	4	672	189
Björn W. Schuller	841	5	26	408	433
Helen M. Meng	717	6	46	337	380
Dong Yu	716	7	63	293	423
Chin Hui P. Lee	710	8	6	544	166
Junichi Yamagishi	685	9	48	332	353
Ming Zhou	680	10	57	315	365
Alex Waibel	679	11	5	580	99
Bin Ma	670	12	10	503	167

As we can see, some authors increased a lot, and even doubled, the number of co-authors and of collaborations in the recent years, whereas there are seven newcomers in the ranking (Björn Schüller, Khalid Choukri, Dong Yu, Alan Black, Helen Meng, Junichi Yamagishi, and Ming Zhou).

If we focus on the past 5 years (2016–2020), we see that only three authors (Haizhou Li, Shri Narayanan, and Yang Liu) are still among the 12 authors with the largest number of co-authors (Table 8), whereas we notice many new names, often of Asian origin (Yue Zhang, Dong Yu, Yu Zhang, Kongaik Lee, Ming Zhou, and Shinji Watanabe) who constitute a new community around the use of supervised or unsupervised machine-learning approaches.

**Table 8:** The 12 authors with the largest number of co-authors in the past 5 years (2016–2020).

**Rank**	**Name**	**#Co-authors**
1	Graham Neubig	193
1	Björn W Schuller	193
3	Yue Zhang	187
4	Dong Yu	175
4	Yu Zhang	175
6	Haizhou Li	161
7	Kongaik Lee	158
8	Shrikanth S. Narayanan	154
9	Ming Zhou	151
10	Shinji Watanabe	145
10	Jan Hajic	145
12	Yang Liu	143

#### Collaboration Graph

The NLP4NLP+5 collaboration graph (refer to Appendix 4) contains 66,995 nodes corresponding to the 66,995 different authors (48,894 in 2015) and163,189 edges between these nodes (162,497 in 2015).

**Figure 14:**
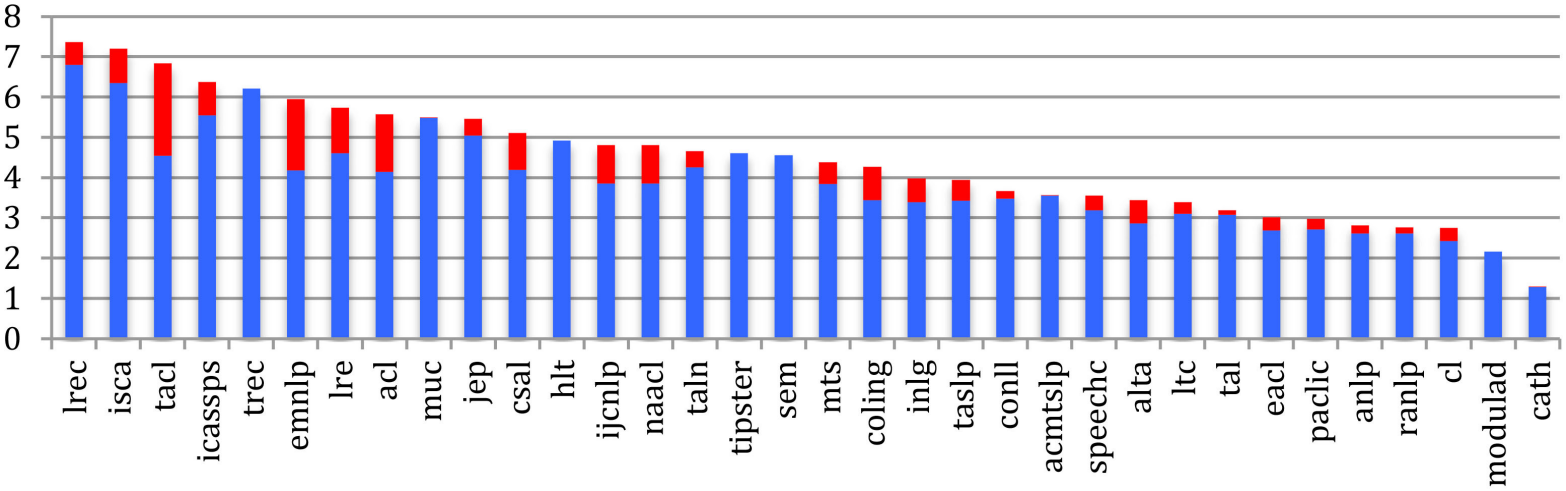
Mean degree of the collaboration graph for the 34 sources in 2015 (blue) and 2020 (red).

When comparing the various sources, we do not notice any meaningful changes between 2015 and 2020 regarding the *diameter, density, average clustering coefficient*, or *connected components* that were presented in our previous paper, whereas the *mean degree* (defined as the average number of co-authors for each author), which illustrates the degree of collaboration within a source, shows a large increase for TACL (4.5–6.9), EMNLP (4.2–5.9), and ACL (4.2–5.6) over this period (Figure 14).

#### Measures of Centrality in the Collaboration Graph

As we see in Table 9, some authors in the top 10 in terms of *closeness centrality* also appear in the two other types of centralities (*degree centrality* and *betweeness centrality*), eventually with a different ranking, whereas others do not. Compared with 2015, we notice “newcomers” among the 10 most “central” authors:

- Yang Liu, Alan Black, Dong Yu (*closeness centrality*: those who are central in a community)
- Björn Schuller, Yang Liu, Khalid Choukri, Ming Zhou, Dong Yu (*degree centrality*: those who most collaborate)
- Laurent Besacier, Alan Black, Sanjeev Khudanpur (*betweenness centrality*: those who make bridges between communities).

**Table 9:** Computation and comparison of the closeness centrality, degree centrality, and betweenness centrality for the 10 most central authors (up to 2020, in comparison with 2015).

**Closeness centrality**					**Degree centrality**				**Betweenness centrality**				
**Rank** ** 2020**	**Previous** ** rank 2015**	**Author's name**	**Harmonic centrality**	**Norm** ** on first**	**Rank** ** 2020**	**Previous** ** rank 2015**	**Author's name**	**Index and** ** norm on first**	**Rank** ** 2020**	**Previous** ** rank 2015**	**Author's name**	**Index**	**Norm on first**
1	8	Sanjeev Khudanpur	17863.281	**1**	1	1	Shrikanth S Narayanan	**1**	1	1	Shrikanth S Narayanan	44717979	**1**
2	5	Haizhou Li	17782.575	**0.995**	2	3	Haizhou Li	**0.881**	2	2	Haizhou Li	34084103	**0.762**
3	2	Shrikanth S Narayanan	17709.094	**0.991**	3	4	Satoshi Nakamura	**0.725**	3	8	Yang Liu	32048199	**0.717**
4	1	Mari Ostendorf	17565.169	**0.983**	4	41	Björn W Schuller	**0.722**	4	3	Satoshi Nakamura	28679912	**0.641**
5	3	Chin Hui P Lee	17454.696	**0.977**	5	12	Yang Liu	**0.72**	5	4	Chin Hui P Lee	25895571	**0.579**
6	6	Julia B Hirschberg	17449.533	**0.977**	6	2	Hermann Ney	**0.715**	6	28	Laurent Besacier	25076596	**0.561**
7	15	Yang Liu	17442.071	**0.976**	7	8	Sanjeev Khudanpur	**0.705**	7	11	Alan W Black	23527696	**0.526**
8	11	Alan W Black	17409.874	**0.975**	8	15	Khalid Choukri	**0.628**	8	10	Khalid Choukri	22889904	**0.512**
9	4	Hermann Ney	17272.551	**0.967**	9	14	Ming Zhou	**0.61**	9	18	Sanjeev Khudanpur	21917631	**0.49**
10	115	Dong Yu	17249.284	**0.966**	10	7	Chin Hui P Lee	**0.598**	10	5	Hermann Ney	21262259	**0.475**
					10	187	Dong Yu	**0.598**					

If we consider the period 2016–2020, we see that only Sanjeev Khudanpur is still among the 10 most central authors, in terms of *closeness centrality* (Table 10).

**Table 10:** Closeness centrality for the 10 most central authors in the past 5 years (2016–2020).

**Rank**	**Name**	**Harmonic centrality**	**Norm on first**
1	Dong Yu	7205.507	**1**
2	Yu Zhang	7109.654	**0.987**
3	Graham Neubig	7103.21	**0.986**
4	Yue Zhang	7012.758	**0.973**
5	Sanjeev Khudanpur	6908.953	**0.959**
6	Heng Ji	6897.558	**0.957**
7	Shinji Watanabe	6881.992	**0.955**
8	Xin Wang	6836.757	**0.949**
9	Mark A. Hasegawa Johnson	6811.851	**0.945**
10	Lukás Burget	6732.778	**0.934**

*Values in bold indicate normalize values based on the first one*.

In addition to that, only three authors among the 10 most “*Betweenness Central*” authors up to 2015 are still in the ranking for the 2016–2020 period (Shri Narayanan, Yang Liu, and Haizhou Li). New authors may bring bridges with new scientific communities. Some authors may be absent from this 2016–2020 ranking, while increasing their global “up to 2020” ranking in this period due to the enlargement of previous communities (Table 11).

**Table 11:** Betweenness centrality for the 10 most central authors in the past 5 years (2016–2020).

**Rank**	**Name**	**Index**	**Norm on first**
1	Yue Zhang	12633450	**1**
2	Graham Neubig	12539019	**0.993**
3	Dong Yu	10394169	**0.823**
4	Yu Zhang	9117498	**0.722**
5	Shrikanth S. Narayanan	8093016	**0.641**
6	Laurent Besacier	7640198	**0.605**
7	Yang Liu	6931507	**0.549**
8	Shinji Watanabe	6751311	**0.534**
9	Haizhou Li	6233480	**0.493**
10	Xin Wang	6096768	**0.483**

### Citations

#### Global Analysis

We studied citations of NLP4NLP+5 papers in the 78,927 NLP4NLP+5 papers that contain a list of references. If we consider the papers that were published in joint conferences as different papers, the number of references is equal to 585,531. If we consider them as the same paper, the number of references in NLP4NLP+5 papers goes down to 535,989 and is equal to the number of citations of NLP4NLP+5 papers.

**Figure 15:**
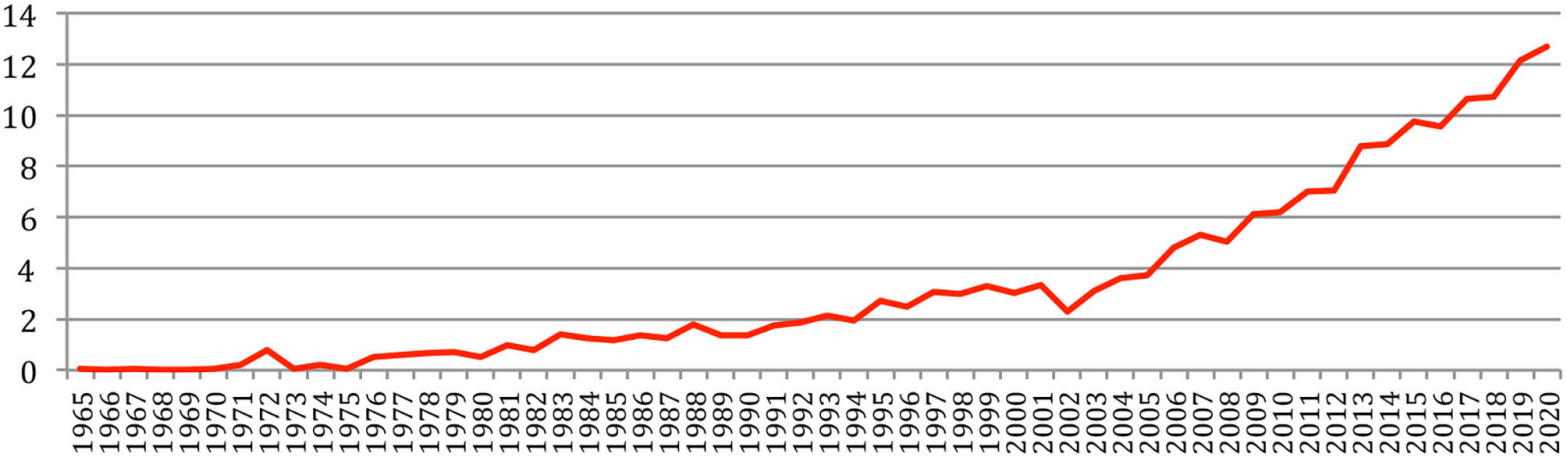
The average number of references per paper over the years.

The average number of NLP4NLP+5 references in NLP4NLP+5 papers increased over time from close to 0 in 1965 to 12.7 in 2020 (was 9.7 in 2015) (Figure 15), as a result of the citing habits and of the increase in the number of published papers.

**Figure 16:**
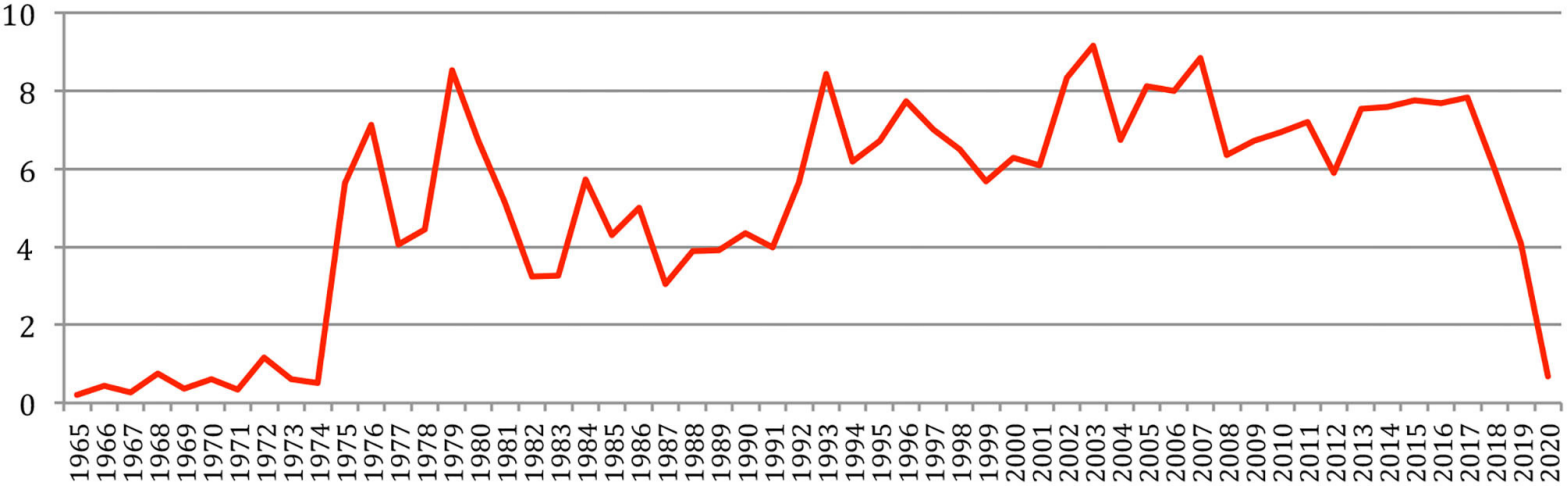
The average number of citations of a paper over the years.

The trend concerning the average number of citations per paper over the years (Figure 16) is less clear. Obviously, the most recent papers are less cited than the older ones, with a number of more than nine citations on average per paper for the papers of the most cited year (2003) and less than one citation on average for the papers published in 2020, given that they can only be cited by the papers published on the same year. It seems that papers need on average 3 years after publication to be properly cited, and that the average number of citations for a paper is stabilized at about 6–8 citations per paper if we consider the period 1993–2018.

Among the 66,995 authors, 23,850 (36%) are never cited (even 25,281 (38%) if we exclude self-citations). These percentages slightly improved compared with 2015 (respectively, 42 and 44%). However, those never cited authors may come from neighboring research domains (artificial intelligence, machine learning, medical engineering, acoustics, phonetics, general linguistics, etc.), where they may be largely cited. Among the 85,138 papers, 31,603 (37%) are never cited [even 40,111 (47%) if we exclude self-citations by the authors of these papers] also showing a slight improvement compared with 2015 (respectively, 44 and 54%) (Table 12).

**Table 12:** Absence of citations of authors and papers within NLP4NLP+5.

	**Number**	**Percentage**	**Previous**
		**2020**	**% 2015**
Papers never referenced	31,603	37	44
Papers never referenced (aside self ref)	40,111	47	54
Authors never referenced	23,850	36	42
Authors never referenced (aside self ref)	25s ,281	38	44

#### Analysis of Authors' Citations

##### Most Cited Authors

Table 13 gives the list of the 20 most cited authors up to 2020, with the number of citations for each author, the number of papers written by the author, and the percentage of self-citation with a comparison to 2015. We may notice that the seven most cited authors up to 2015 are still present in 2020, but that 50% of the authors of 2020 (mostly attached to the machine learning and word embedding research-based communities) are newcomers in this ranking.

**Table 13:** A total of 20 most cited authors up to 2020.

**Rank 2020**	**Previous rank 2015**	**Name**	**#Citations**	**Nb of papers written by this author**	**Ratio #citations/nb of papers written by this author**	**Percentage of self-citations**
1	3	Christopher D. Manning	13,195	152	86.809	2.145
2	1	Hermann Ney	7,109	388	18.322	16.205
3	>20	Christopher Dyer	5,372	114	47.123	3.984
4	>20	Richard Socher	5,175	37	139.865	1.198
5	2	Franz Josef Och	5,041	42	120.024	1.825
6	5	Dan Klein	4,945	130	38.038	6.249
7	4	Philipp Koehn	4,726	59	80.102	2.412
8	>20	Noah A. Smith	4,648	160	29.05	6.713
9	7	Andreas Stolcke	4,532	145	31.255	6.355
10	6	Michael John Collins	4,256	69	61.681	3.195
11	>20	Kenton Lee	4,251	21	202.429	0.729
12	>20	Luke S. Zettlemoyer	4,158	92	45.196	5.075
13	9	Salim Roukos	4,132	71	58.197	1.5
14	18	Daniel Jurafsky	4,056	118	34.373	2.342
15	>20	Kristina Toutanova	4,055	47	86.277	0.764
16	>20	Sanjeev Khudanpur	4,051	135	30.007	6.492
17	>20	Daniel Povey	3,796	112	33.893	7.929
18	16	Li Deng	3,672	201	18.269	14.842
19	>20	Dong Yu	3,653	177	20.638	10.895
20	>20	Mirella Lapata	3,578	138	25.928	6.987

Table 14 provides the number of citations, either by themselves (self-citations) or by others (external citations), for the most productive authors that appear in Table 3. We see that only two of the 20 most productive authors (Herman Ney, Li Deng) also appear among the 20 most cited authors.

**Table 14:** The number of citations for the 20 most productive authors (1965–2020).

**Number of written papers**	**Name**	**#as first author**	**% as first author**	**#as last author**	**% as last author**	**#as sole author**	**% as sole author**	**Rank citations**	**#self-citations**	**Ratio of #self-citations/** **number of written papers**	**#external citations**	**Ratio of #external citations/number of written papers**
453	Shrikanth S. Narayanan	13	3	388	86	0	0	>20	782	1.726	2,129	4.7
388	Hermann Ney	27	7	325	84	10	3	2	1,152	2.969	5,957	15.353
354	John H. L. Hansen	29	8	283	80	3	1	>20	779	2.201	1,076	3.04
350	Haizhou Li	13	4	256	73	2	1	>20	490	1.4	1,623	4.637
263	Satoshi Nakamura	17	6	190	72	1	0	>20	160	0.608	648	2.464
261	Chin Hui P. Lee	14	5	207	79	5	2	>20	577	2.211	2,852	10.927
234	Alex Waibel	13	6	199	85	2	1	>20	262	1.12	2,048	8.752
230	Mark J. F. Gales	31	13	105	46	9	4	>20	638	2.774	2,923	12.709
214	James R. Glass	11	5	152	71	1	0	>20	428	2	2,084	9.738
209	Yang Liu	48	23	83	40	3	1	>20	240	1.148	2,080	9.952
204	Lin Shan Lee	10	5	189	93	0	0	>20	328	1.608	656	3.216
201	Li Deng	57	28	73	36	6	3	18	545	2.711	3,127	15.557
197	Hervé Bourlard	10	5	141	72	3	2	>20	277	1.406	940	4.772
195	Mari Ostendorf	29	15	100	51	5	3	>20	309	1.585	2,136	10.954
195	Tatsuya Kawahara	33	17	110	56	0	0	>20	248	1.272	708	3.631
192	Björn W. Schuller	40	21	105	55	0	0	>20	511	2.661	1,583	8.245
188	Keikichi Hirose	28	15	95	51	1	1	>20	140	0.745	330	1.755
183	Frank K. Soong	9	5	78	43	0	0	>20	208	1.137	1,240	6.776
182	Kiyohiro Shikano	1	1	142	78	0	0	>20	276	1.516	1,161	6.379
180	Timothy Baldwin	21	12	115	64	4	2	>20	216	1.2	1,160	6.444

We may express that the publishing profile is very different among authors. The authors who publish a lot are not necessarily the ones who are the most cited (from 1.75 to 15 citations per paper on average) and the role of authors varies, from the main contributor to team manager, depending on their place in the authorship list and the cultural habits. Some authors are used to cite their own papers, while others are not (from 0.6 to 2.9 citations of their own paper on average).

If we now only consider the 2016–2020 period (papers published over 55 years that are cited in this 5-year period) (Table 15), we see that only one author of the 2015 ranking (Chris Manning) is still among the 20 most cited authors for this period!

**Table 15:** A number of 20 most cited authors in the past 5 years (2016–2020).

**Rank**	**Name**	**#Citations**	**#Papers written by this author**	**Ratio #citations/#papers written by this author**	**Percentage of self-citations**
1	Christopher D. Manning	9,148	152	60.184	0.875
2	Richard Socher	4,404	37	119.027	0.749
3	Kenton Lee	4,250	21	202.381	0.729
4	Christopher Dyer	3,881	114	34.044	3.015
5	Luke S. Zettlemoyer	3,640	92	39.565	3.407
6	Sanjeev Khudanpur	3,168	135	23.467	5.966
7	Kristina Toutanova	3,154	47	67.106	0.254
8	Noah A. Smith	3,115	160	19.469	4.687
9	Ming Wei Chang	2,990	31	96.452	1.204
10	Daniel Povey	2,852	112	25.464	6.872
11	Jacob Devlin	2,836	20	141.8	0.353
12	Jeffrey Pennington	2,586	2	1293	0
13	Percy Liang	2,312	56	41.286	3.287
14	Dong Yu	2,238	177	12.644	6.702
15	Tomáš Mikolov	2,232	18	124	0.314
16	Yoshua Bengio	2,170	47	46.17	2.074
17	Mirella Lapata	2,106	138	15.261	7.123
18	Daniel Jurafsky	2,002	118	16.966	1.049
19	Eduard H. Hovy	1,970	168	11.726	2.69
20	Yoav Goldberg	1,860	72	25.833	2.527

Some authors who published a small number of seminal papers got a huge number of citations (such as Jeffrey Pennington, for the “Glove paper,” with two papers totaling 2,586 citations, with no self-citation!). However, as it will appear in the next section, getting a high h-index necessitates both publishing a lot and having a lot of citations of these published papers.

##### Authors' H-Index

Despite the criticisms that are attached to this measure and as it was included in our previous paper, we computed the h-index of the authors based only on the papers published in the NLP4NLP+5 corpus. Table 16 provides the list of the 20 authors with the largest h-index up to 2020, with a comparison to 2015 (based on the papers published and cited in the respective 55- and 50-year time periods). We see that Christopher Manning has still the largest h-index: he published 49 papers, which were cited at least 49 times. About 55% of the authors with highest h-index up to 2015 are still in the top 20 authors with highest h-index up to 2020, while 45% are newcomers (also mostly coming from the machine learning and word embedding research-based communities).

**Table 16:** List of the 20 authors with the largest h-index up to 2020 in comparison with 2015.

**Rank**	**Previous**	**Name**	**h-index**	**Previous**
**2020**	**Rank**		**2020**	**h-Index**
	**2015**			**2015**
1	1	Christopher D. Manning	49	32
2	12	Noah A. Smith	36	22
3	4	Dan Klein	35	25
4	2	Hermann Ney	34	29
5	12	Daniel Jurafsky	33	22
6	15	Mirella Lapata	33	21
7	12	Li Deng	33	22
8	3	Andreas Stolcke	32	28
9	>20	Christopher Dyer	31	
10	>20	Luke S. Zettlemoyer	31	
11	>20	Kevin Knight	29	
12	5	Michael John Collins	29	24
13	>20	Dan Roth	28	
14	>20	Dong Yu	28	
15	>20	Regina Barzilay	27	
16	12	Stephen J. Young	27	22
17	>20	Eduard H. Hovy	27	
18	>20	Daniel Povey	27	
19	15	Joakim Nivre	27	21
20	>20	Deliang Wang	26	

If we consider the h-index in the past 5 years (based on the papers published on 55 years and cited in the 2016–2020 period) (Table 17), we see that only five authors (Chris Manning, Noah Smith, Dan Klein, Daniel Jurafsky, and Mirella Lapata) with highest h-index up to 2015 are still in the top 20 authors with highest h-index for the 2016–2020 period!

**Table 17:** List of the 20 authors with the largest h-index for the past 5 years (2016–2020).

**Rank**	**Name**	**h-index 2020**
1	Christopher D. Manning	38
2	Noah A. Smith	31
3	Christopher Dyer	29
4	Luke S. Zettlemoyer	28
5	Mirella Lapata	26
6	Daniel Jurafsky	23
7	Dong Yu	23
8	Daniel Povey	22
9	Tara N. Sainath	22
10	Dan Klein	22
11	Yoav Goldberg	22
12	Percy Liang	21
13	Dan Roth	21
14	Yang Liu	21
15	Shinji Watanabe	20
16	Sanjeev Khudanpur	20
17	Regina Barzilay	20
18	Deliang Wang	20
19	Björn W. Schuller	20
20	Yue Zhang	20

#### Analysis of Papers' Citations

##### Most Cited Papers

Table 18 provides the list of the 20 most cited papers up to 2020 and a comparison with 2015. A number of 11 (55%) of the 20 most cited papers up to 2015 are still among the 20 most cited papers up to 2020, whereas it includes five newcomers and four papers published in or after 2015, with a special emphasis on word embedding and deep learning (Glove and BERT). The most cited paper up to 2015 is still the most cited up to 2020 (BLEU MT evaluation measure). The most cited papers are still mostly those related to language data (Penn Treebank, Wordnet, and Europarl), evaluation metrics (BLEU), language processing tools (Glove, BERT, Moses, SRILM), or methodology surveys (word representations, statistical alignment, statistical and neuronal machine translation). The largest number of highly cited papers comes from the ACL conference (4), NAACL (3), the *Computational Linguistics* journal (3), and the *IEEE TASLP* (3), whereas four papers now come from the EMNLP conference, which was previously absent from this ranking.

**Table 18:** The number of 20 most cited papers up to 2020.

**Rank** ** 2020**	**Rank** ** 2015**	**Title**	**Authors**	**Source**	**Year**	**#Citations 2020**	**#Citations**
1	1	BLEU: a Method for Automatic Evaluation of Machine Translation	Kishore A. Papineni, Salim Roukos, Todd R. Ward, Wei Jing Zhu	acl	2002	3,020	1514
2	>20	Glove: Global Vectors for Word Representation	Jeffrey Pennington, Richard Socher, Christopher D. Manning	emnlp	2014	2,590	
3	0	BERT: Pre-training of Deep Bidirectional Transformers for Language Understanding	Jacob Devlin, Ming Wei Chang, Kenton Lee, Kristina Toutanova	naacl	2019	2,468	
4	2	Building a Large Annotated Corpus of English: The Penn Treebank	Mitchell P. Marcus, Beatrice Santorini, Mary Ann Marcinkiewicz	cl	1993	1,610	1145
5	3	Moses: Open Source Toolkit for Statistical Machine Translation	Philipp Koehn, Hieu Hoang, Alexandra Birch, Chris Callison Burch, Marcello Federico, Nicola Bertoldi, Brooke Cowan, Wade Shen, Christine Moran, Richard Zens, Christopher Dyer, Ondrej Bojar, Alexandra Constantin, Evan Herbst	acl	2007	1,380	860
6	5	SRILM - an extensible language modeling toolkit	Andreas Stolcke	isca	2002	1,319	831
7	>20	Front-End Factor Analysis for Speaker Verification	Najim Dehak, Patrick J. Kenny, Réda Dehak, Pierre Dumouchel, Pierre Ouellet	taslp	2011	1,170	
8	0	Deep Contextualized Word Representations	Matthew E. Peters, Mark Neumann, Mohit Iyyer, Matt Gardner, Christopher Clark, Kenton Lee, Luke S. Zettlemoyer	naacl	2018	1,166	
9	4	A Systematic Comparison of Various Statistical Alignment Models	Franz Josef Och, Hermann Ney	cl	2003	1,079	855
10	6	Statistical Phrase-Based Translation	Philipp Koehn, Franz Josef Och, Daniel Marcu	hlt, naacl	2003	1,038	829
11	7	The Mathematics of Statistical Machine Translation: Parameter Estimation	Peter E. Brown, Stephen A. Della Pietra, Vincent J. Della Pietra, Robert L. Mercer	cl	1993	978	820
12	0	Effective Approaches to Attention-based Neural Machine Translation	Thang Luong, Hieu Pham, Christopher D. Manning	emnlp	2015	907	
13	8	Minimum Error Rate Training in Statistical Machine Translation	Franz Josef Och	acl	2003	879	726
14	>20	Convolutional Neural Networks for Sentence Classification	Yoon Chul Kim	emnlp	2014	862	
15	0	Neural Machine Translation of Rare Words with Subword Units	Rico Sennrich, Barry Haddow, Alexandra Birch	acl	2016	836	
16	>20	Wordnet: A Lexical Database For English	George A. Miller	hlt	1992	814	
17	>20	Spoken Language Translation	Hwee Tou Ng	emnlp	1997	774	
18	15	Europarl: A Parallel Corpus for Statistical Machine Translation	Philipp Koehn	mts	2005	760	472
19	10	Suppression of acoustic noise in speech using spectral subtraction	Steven F. Boll	taslp	1979	728	566
20	13	Speech enhancement using a minimum-mean square error short-time spectral amplitude estimator	Yariv Ephraim, David Malah	taslp	1984	708	488

While if we only consider the 20 most cited papers in the period of 2016–2020 (papers published over 55 years that are cited in this 5-year period) (Table 19), 75% of those papers were not in the 2015 ranking!

**Table 19:** The number of 20 most cited papers for the past 5 years (2016–2020).

**Rank**	**Name and title**	**Corpus**	**Year**	**Authors**	**#Citations 2016–2020**
1	Glove: Global Vectors for Word Representation	emnlp	2014	Jeffrey Pennington, Richard Socher, Christopher D. Manning	2,486
2	BERT: Pre-training of Deep Bidirectional Transformers for Language Understanding	naacl	2019	Jacob Devlin, Ming Wei Chang, Kenton Lee, Kristina Toutanova	2,468
3	BLEU: a Method for Automatic Evaluation of Machine Translation	acl	2002	Kishore A. Papineni, Salim Roukos, Todd R. Ward, Wei Jing Zhu	1,491
4	Deep Contextualized Word Representations	naacl	2018	Matthew E. Peters, Mark Neumann, Mohit Iyyer, Matt Gardner, Christopher Clark, Kenton Lee, Luke S. Zettlemoyer	1,166
5	Effective Approaches to Attention-based Neural Machine Translation	emnlp	2015	Thang Luong, Hieu Pham, Christopher D. Manning	907
6	Neural Machine Translation of Rare Words with Subword Units	acl	2016	Rico Sennrich, Barry Haddow, Alexandra Birch	836
7	Convolutional Neural Networks for Sentence Classification	emnlp	2014	Yoon Chul Kim	820
8	Front-End Factor Analysis for Speaker Verification	taslp	2011	Najim Dehak, Patrick J. Kenny, Réda Dehak, Pierre Dumouchel, Pierre Ouellet	738
9	Enriching Word Vectors with Subword Information	tacl	2017	Piotr Bojanowski, Edouard Grave, Armand Joulin, Tomáš Mikolov	687
10	Learning Phrase Representations using RNN Encoder–Decoder for Statistical Machine Translation	emnlp	2014	Kyunghyun Cho, Bart Van Merrienboer, Caglar Gulçehre, Dzmitry Bahdanau, Fethi Bougares, Holger Schwenk, Yoshua Bengio	566
11	SQuAD: 100,000+ Questions for Machine Comprehension of Text	emnlp	2016	Pranav Rajpurkar, Jian Justin Zhang, Konstantin Lopyrev, Percy Liang	556
12	Moses: Open Source Toolkit for Statistical Machine Translation	acl	2007	Philipp Koehn, Hieu Hoang, Alexandra Birch, Chris Callison Burch, Marcello Federico, Nicola Bertoldi, Brooke Cowan, Wade Shen, Christine Moran, Richard Zens, Christopher Dyer, Ondrej Bojar, Alexandra Constantin, Evan Herbst	505
13	Recursive Deep Models for Semantic Compositionality Over a Sentiment Treebank	emnlp	2013	Richard Socher, Alex Perelygin, Jean Wu, Jason Chuang, Christopher D. Manning, Andrew Y. Ng, Christopher Potts	488
14	Librispeech: An ASR Corpus Based on Public Domain Audio Books	icassps	2015	Vassil Panayotov, Guoguo Chen, Daniel Povey, Sanjeev Khudanpur	474
15	Recurrent neural network-based language model	isca	2010	Tomáš Mikolov, Martin Karafiát, Lukás Burget, Jan Honza Cernocký, Sanjeev Khudanpur	472
16	Wordnet: A Lexical Database for English	hlt	1992	George A. Miller	456
17	Get To The Point: Summarization with Pointer-Generator Networks	acl	2017	Abigail See, Peter J. Liu, Christopher D. Manning	455
18	Building a Large Annotated Corpus of English: The Penn Treebank	cl	1993	Mitchell P. Marcus, Beatrice Santorini, Mary Ann Marcinkiewicz	447
19	A large annotated corpus for learning natural language inference	emnlp	2015	Samuel R. Bowman, Gabor Angeli, Christopher Potts, Christopher D. Manning	446
20	Neural Architectures for Named Entity Recognition	naacl	2016	Guillaume Lample, Miguel Ballesteros, Sandeep Subramanian, Kazuya Kawakami, Christopher Dyer	432

#### Analysis of Citations Among NLP4NLP Sources

##### Comparison of NLP vs. Speech Processing Sources

**Figure 17:**
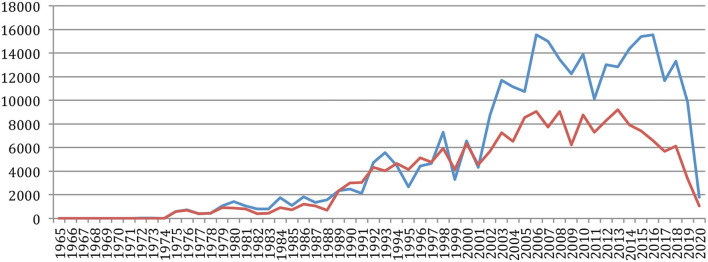
Number of NLP (blue) vs. Speech (red) articles being cited over time.

When comparing the number of articles being cited in NLP vs. speech-oriented publications (Figure 17), we see that this number is increasing much more importantly in the NLP ones since 2001, providing that 2020 cannot be considered due to the previously expressed reason.

**Figure 18:**
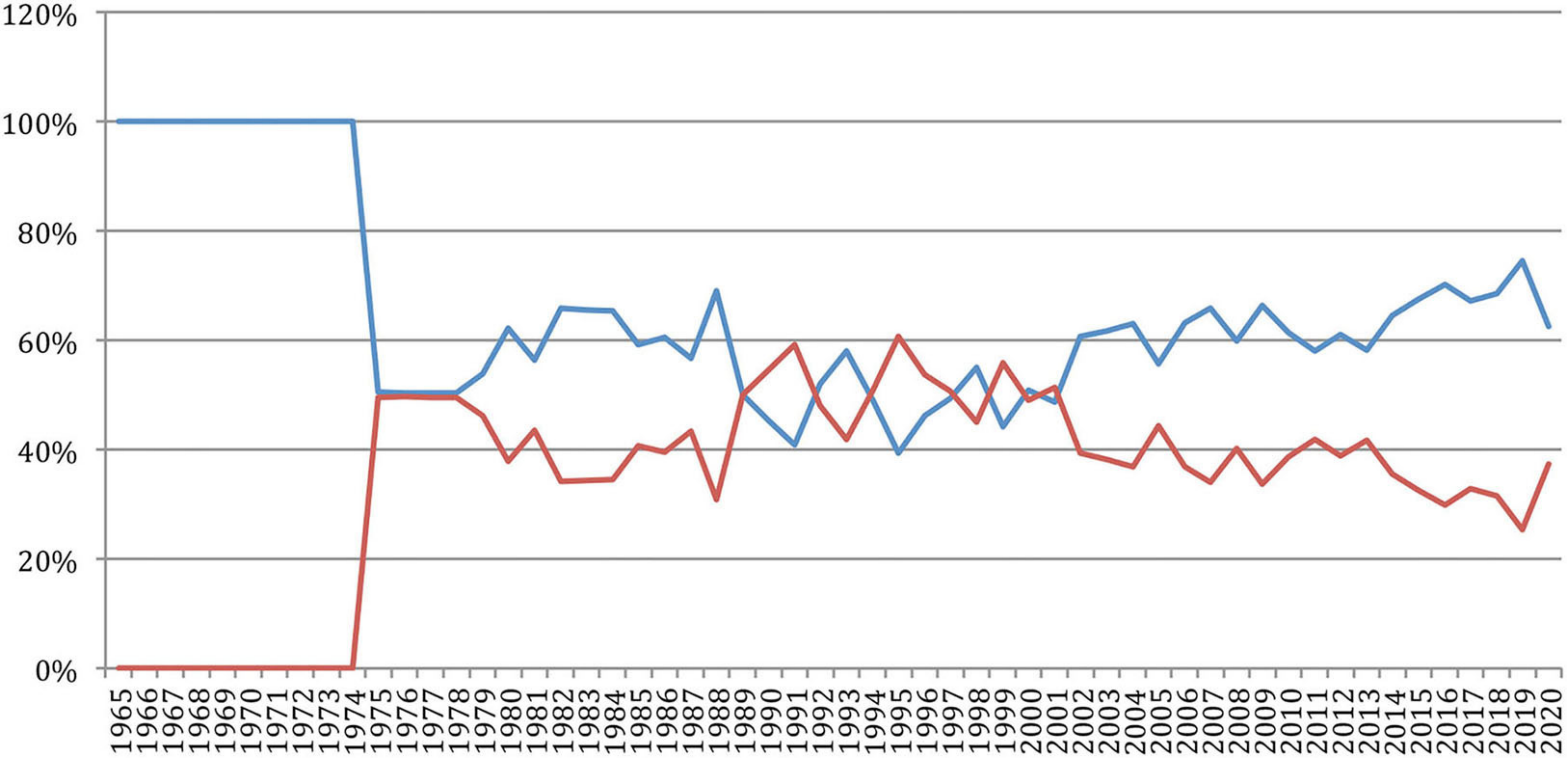
Percentage of NLP (blue) vs. Speech (red) articles being cited over time.

This is also reflected in the ratio of NLP vs. speech articles' citations (Figure 18), given that we only had NLP sources until 1975. We then had a ratio of about 60% of NLP papers being cited from 1975 to 1989, then a balanced ratio until 2001, and since then an increasing percentage of NLP papers which attained 75% in 2019.

##### Comparison of Citations for Six Major Conferences and Journals

**Figure 19:**
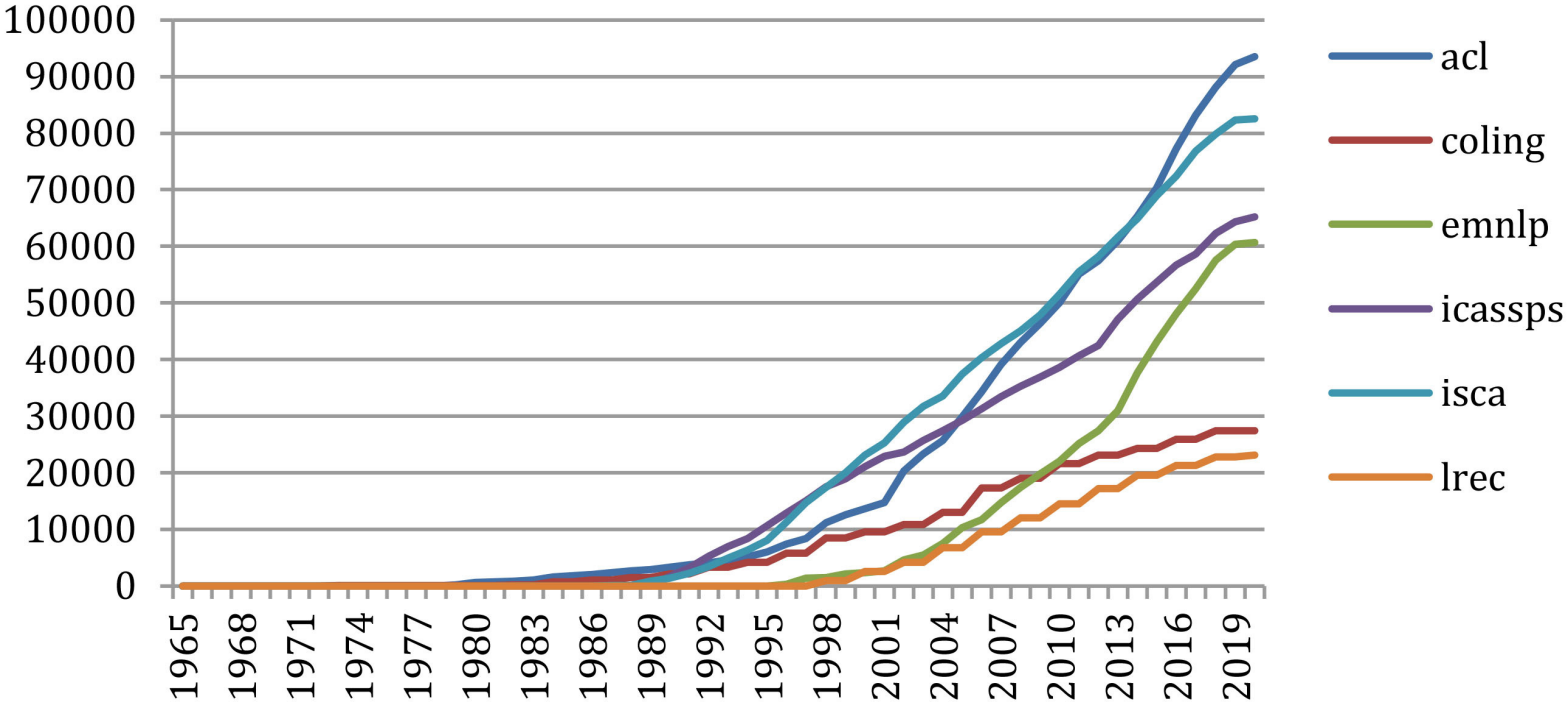
Number of references to papers of 6 major conferences over the years.

The comparative study of the number of cumulative citations of previously published papers in six important conferences (ACL, COLING, EMNLP, ICASSPS, ISCA, and LREC) shows (Figure 19) that the number of ISCA papers being cited grows at a high rate over time, in agreement with the ISCA Board policy which decided in 2005 to enlarge the number of pages from 6 to 7, providing that the allowed extra page should only consist of references. The same appears more recently for ACL. ICASSPS comes in the third position, whereas EMNLP recently showed an important increase. We then find a group of two with COLING and LREC.

**Figure 20:**
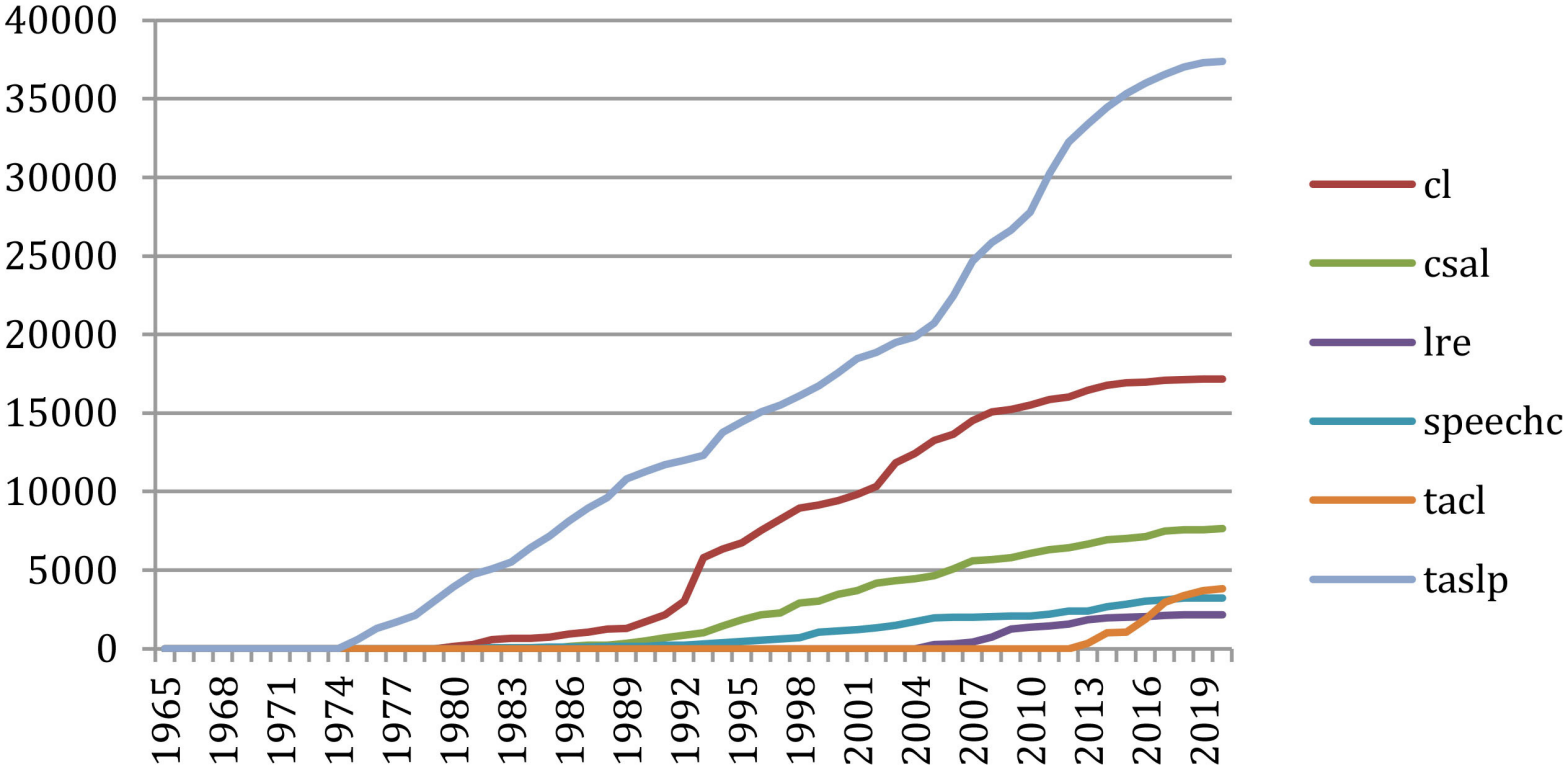
Number of references to papers of 6 major journals over the years.

Doing the same on six major journals (*Computational Linguistics, Computer Speech and Language, Language Resources and Evaluation, Speech Communication, IEEE Transactions on Audio, Speech, and Language Processing, and Transactions of the ACL*) shows (Figure 20) the importance of the reference to the *IEEE Transactions*, followed by *Computational Linguistics*. The *Transactions of the ACL* recently made a large increase.

##### Citation Graph

We considered (refer to Appendix 4) the 85,138 papers and the 66,995 authors in NLP4NLP+5 in the citation graph, which includes 587,000 references. When comparing the sources, it should be remembered that the time periods are different, as well as the frequency and number of events for conferences or journals.

*Authors' Citation Graph*. When comparing the various sources, there are also no meaningful changes between 2015 and 2020 regarding the *diameter, density, average clustering coefficient*, and *connected components* of the *Internal Authors Citations* and *Ingoing Global Authors Citations* that were presented in our previous paper.

**Figure 21:**
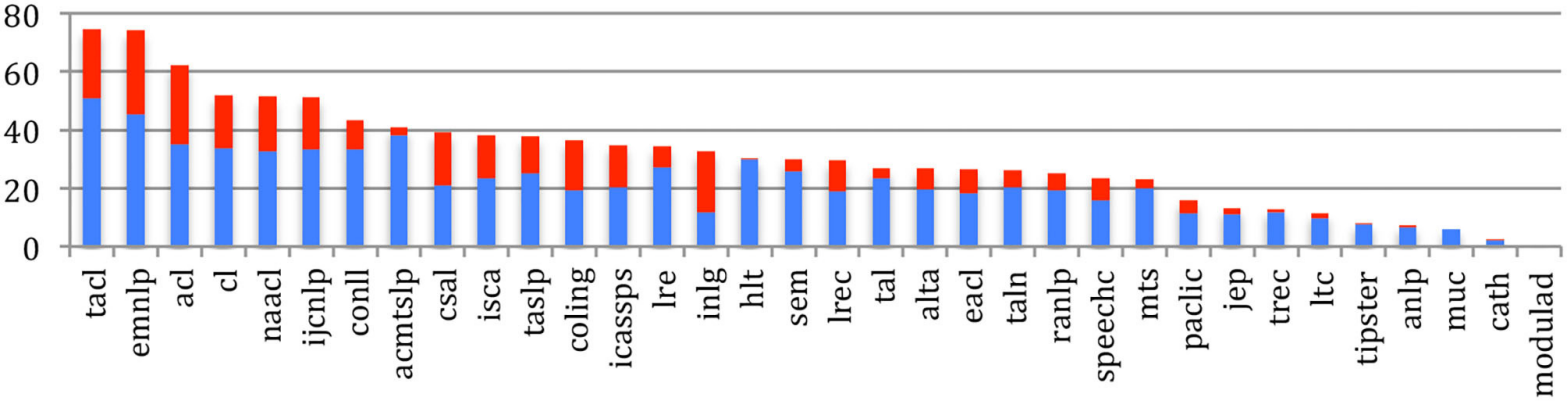
Mean Degree of authors citing authors in general for the 34 sources in 2015 (blue) and 2020 (red).

The mean degree of the *Outgoing Global Authors Citations* graph of the **citing** authors (i.e., average number of authors being cited by each author), measuring the average number of authors citations within a source, shows a large increase for most sources (Figure 21), following the general trend (refer to Figure 15), especially recently in the NLP sources (TACL, EMNLP, ACL, CL, NAACL, IJCNLP, and CONLL) with more than 40 authors being cited by each author on average.

**Figure 22:**
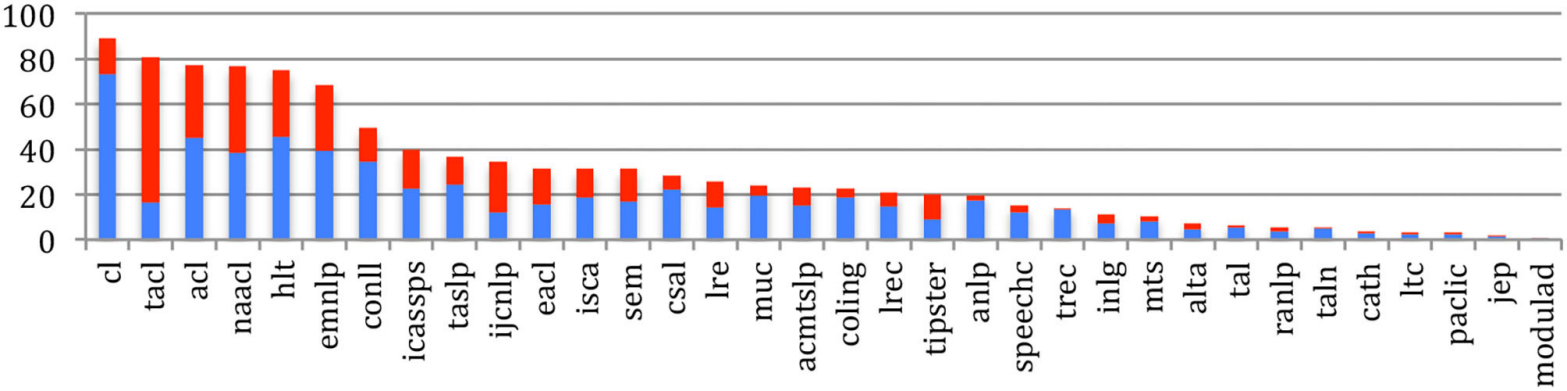
Mean Degree of authors being cited for the 34 sources in 2015 (blue) and 2020 (red).

The mean degree of the *Ingoing Global Authors Citations* graph of the authors **being cited** in each of the 34 sources (Figure 22) shows that authors who publish in *Computational Linguistics* are still the most cited, but are now closely followed by authors in TACL, with a tremendous increase, then ACL, NACL, HLT, and EMNLP, with more than 60 citations of each author on average.

*Papers' Citation Graph*. There are no meaningful changes between 2015 and 2020 regarding the *diameter, density, average clustering coefficient, and connected components* of the *Internal Papers Citations* and *Ingoing Global Papers Citations*, when comparing the various sources.

**Figure 23:**
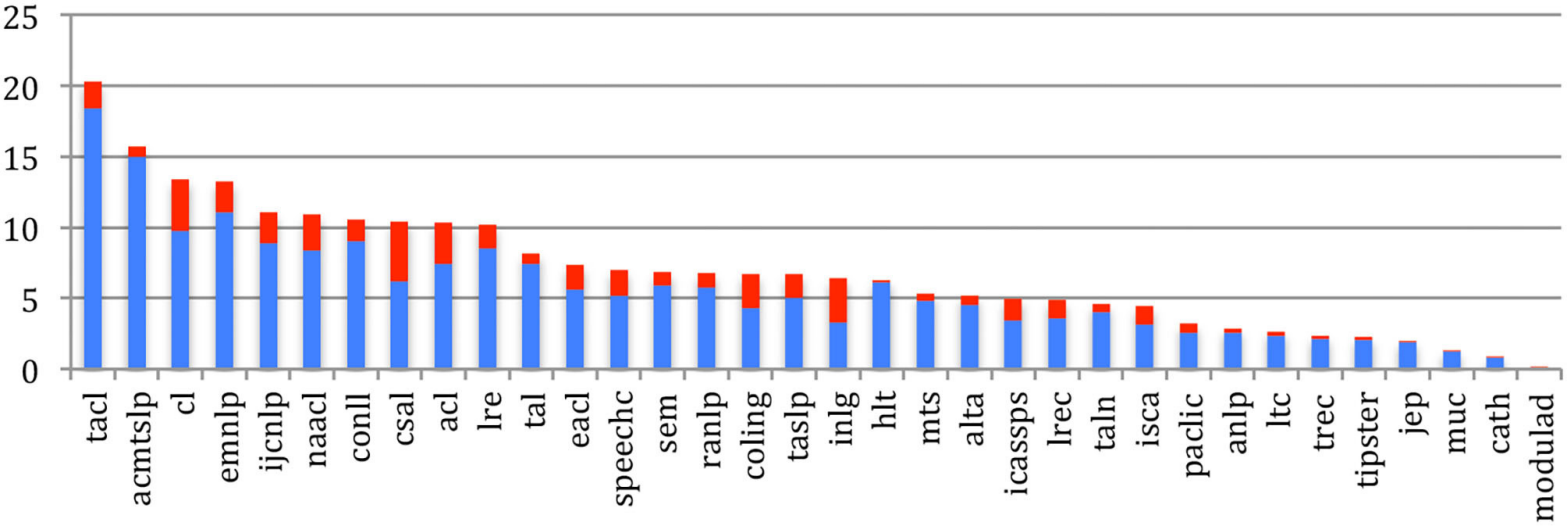
Mean Degree of papers citing papers in general for the 34 sources in 2015 (blue) and 2020 (red).

The mean degree of the *Outgoing Global Papers Citations* graph of the **citing** papers (i.e., average number of references in each paper), measuring the average number of papers citations within a source, shows an increase for most sources (Figure 23), following the general trend (refer to Figure 15), especially the NLP sources (TACL, CL, EMNLP, ACL, NAACL, IJCNLP, CONLL, CSAL, and LRE) with more than 10 references in each paper on average.

**Figure 24:**
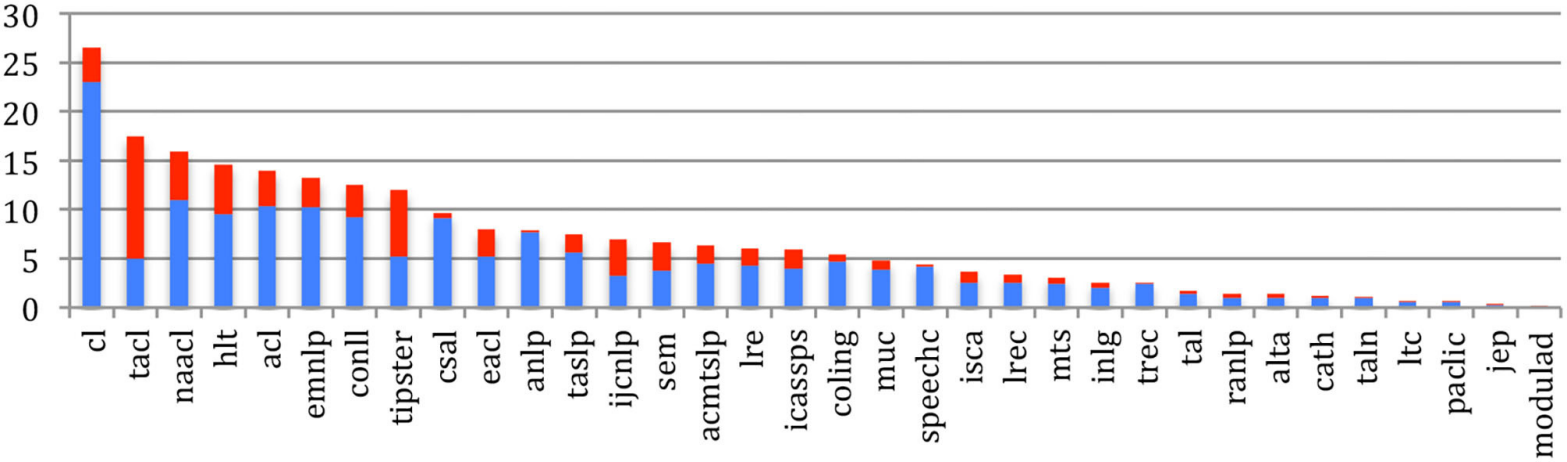
Mean Degree of papers being cited for the 34 sources in 2015 (blue) and 2020 (red).

Figure 24 provides the average number of papers being cited from each of the 34 sources. Papers published in *Computational Linguistics* are still the most cited (more than 25 times on average), but are now more closely followed by various NLP sources (TACL (with a tremendous increase), NAACL, HLT, ACL, EMNLP, and CONLL), with more than 10 citations of each paper on average.

##### Sources' H-Index

**Figure 25:**
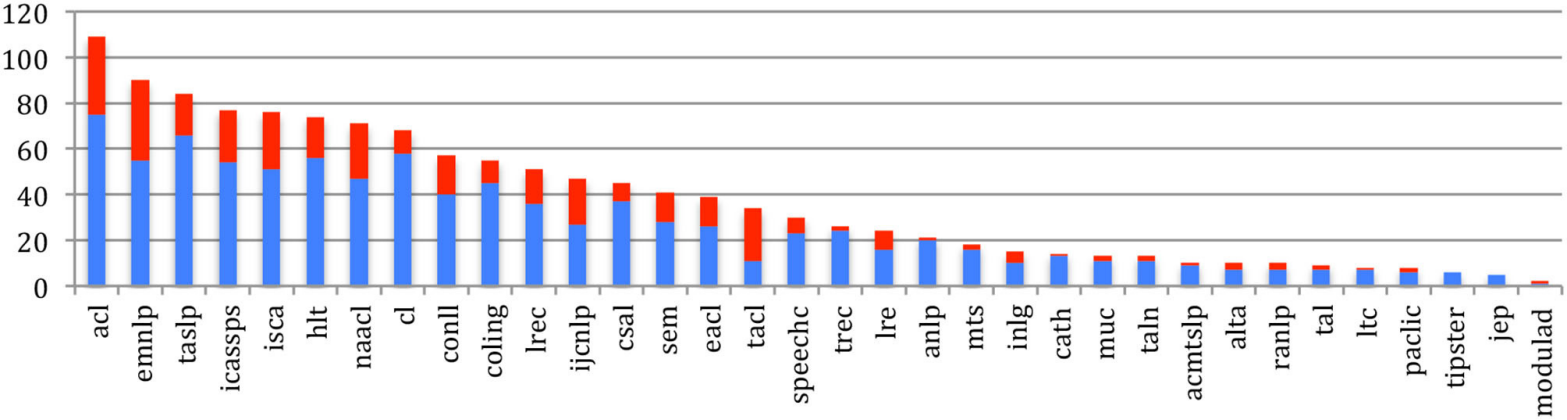
Internal h-index of the 34 sources in 2015 (blue) and 2020 (red).

Figure 25 provides the internal h-index (NLP4NLP papers being cited by papers of any NLP4NLP source) for the 34 sources. The largest h-index is obtained by ACL, where 109 papers are cited 109 times or more in the NLP4NLP+5 papers, followed by EMNLP, which increased considerably its h-index over the past 5 years from 55 in 2015 to 90 in 2020, TASLP (84), ICASSPS (77), and ISCA Interspeech (76).

#### Analysis of the Citation in NLP4NLP Papers of Sources From the Scientific Literature Outside NLP4NLP

##### Extraction of References

In the internal NLP4NLP citation analysis, references were extracted through a highly reliable checking of titles, as we possess the knowledge of the NLP4NLP paper titles. We cannot use the same approach if we want to explore the citation of articles that were published in other sources than the NLP4NLP ones, as we do not have a list of the titles of all those articles. We therefore used a different strategy based on the use of the ParsCit software (Councill et al., 2008) to identify the sources within the reference sections of articles for a limited set of NLP4NLP articles. This new process is resulted in a list of raw variants of source naming, which necessitated a manual cleaning, as it contained a lot of noise, followed by normalization and categorization in four categories (Conferences, Workshops, Journals, and Books).

All the cleaned variants for a given source are kept, for instance, a short name compared to an extended name. We then implemented an algorithm to detect the source names within the reference sections for all NLP4NLP papers. The detection is technically conducted by means of an intermediate computation of a robust key made of uppercase letters and normalization of separators, as the aim is to compare names on the ground of significant characters and to ignore noise and unsignificant details. We then use these data to compare the citations in NLP4NLP articles of the articles published within and outside NLP4NLP sources^8^.

##### Global Analysis

Starting from 32,918 entries, we conducted the manual cleaning and categorization process which resulted in 13,017 different variants of the sources, and, after normalization, in the identification of 3,311 different sources outside the 34 NLP4NLP ones, corresponding to conferences (1,304), workshops (669), journals (1,109), and books (229).

**Figure 26:**
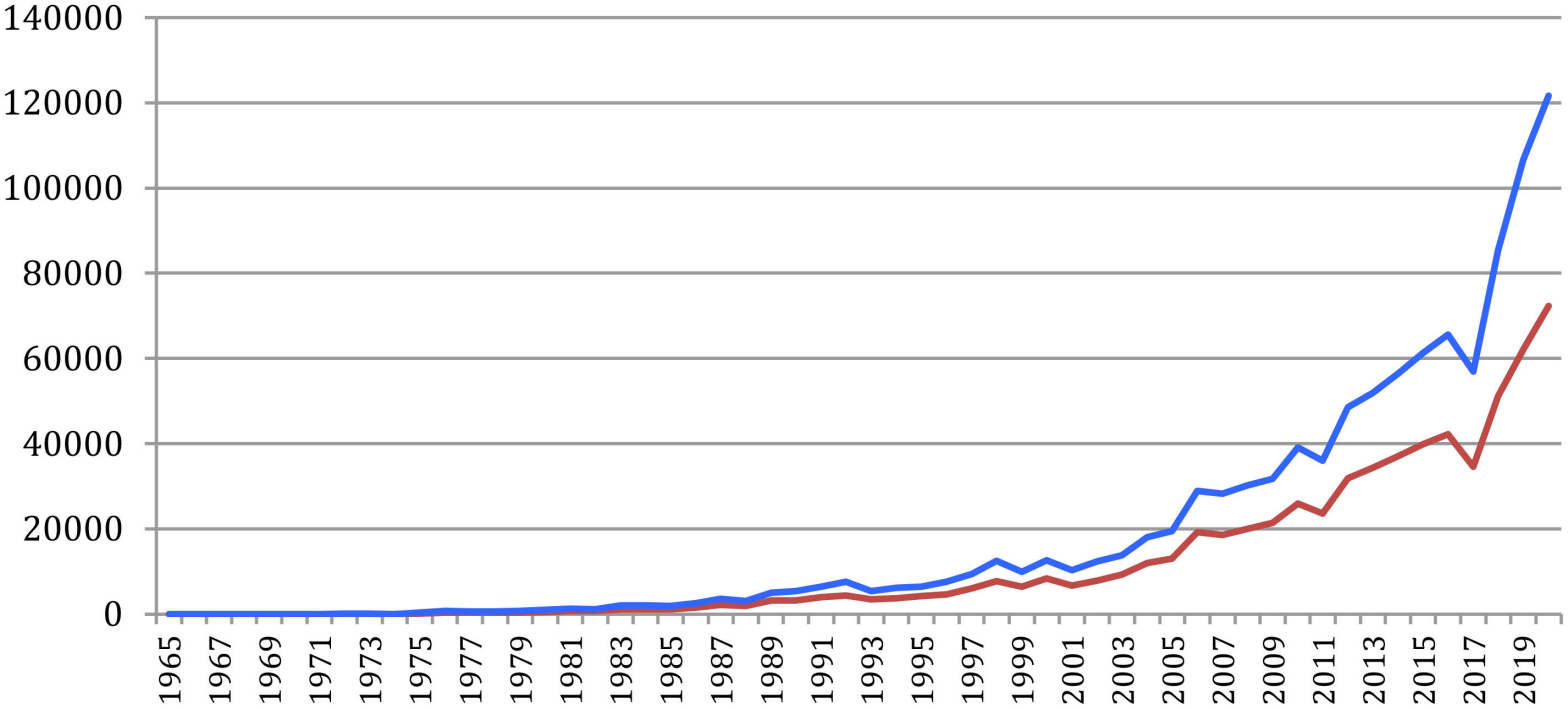
Total number of references (blue) and of NLP4NLP references (red) in NLP4NLP papers yearly.

Figure 26 provides the evolution of the total number of references, which attains 121,619 references in 2020 for a cumulated total of 1,038,468 references over the years, and of NLP4NLP references, which attains 72,289 in 2020 for a cumulated total of 654,340 references (63% of the total number of references) with this new calculation based on source detection. These numbers clearly illustrate the representativity of the 34 NLP4NLP sources totaling close to 20,000 references on average per source, compared with about 110 references on average per source for the 3,311 non-NLP4NLP sources.

**Figure 27:**
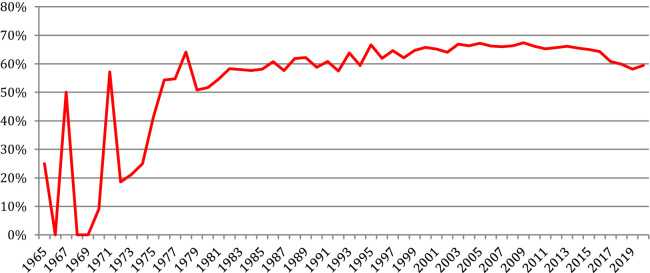
Percentage of NLP4NLP papers in the references.

Figure 27 provides the percentage of NLP4NLP papers in the references. After a hectic period both due to the small quantity and low quality of data, mostly OCRized, until 1976, the ratio of NLP4NLP references stabilized at about 60% until 1994. It then rose up to 67% in 2009 and slowly decreased since then to attain 60% in 2020 with the appearance of new publications.

**Figure 28:**
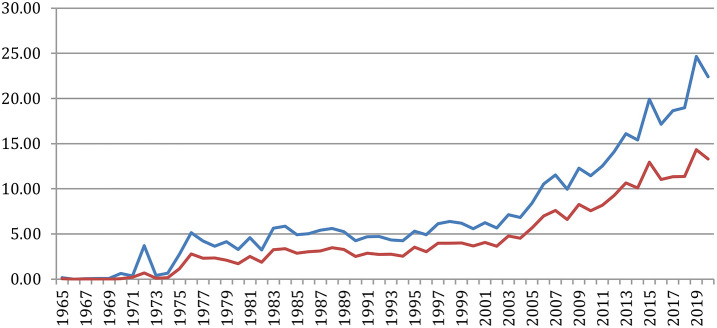
Average number of references per paper globally (blue) or only to NLP4NLP papers (red).

Figure 28 provides the average number of references per paper globally and specifically to NLP4NLP papers. We see that this number increases similarly to attain an average of 25 references per paper, as a result of the citing habits, the increase of the number of publications and of published papers in the literature and the generalization of electronic publishing, as already expressed in section Global Analysis (Figure 15), where only NLP4NLP papers were considered based on title identification.

##### Specific Analysis of Non-NLP4NLP Sources

**Figure 29:**
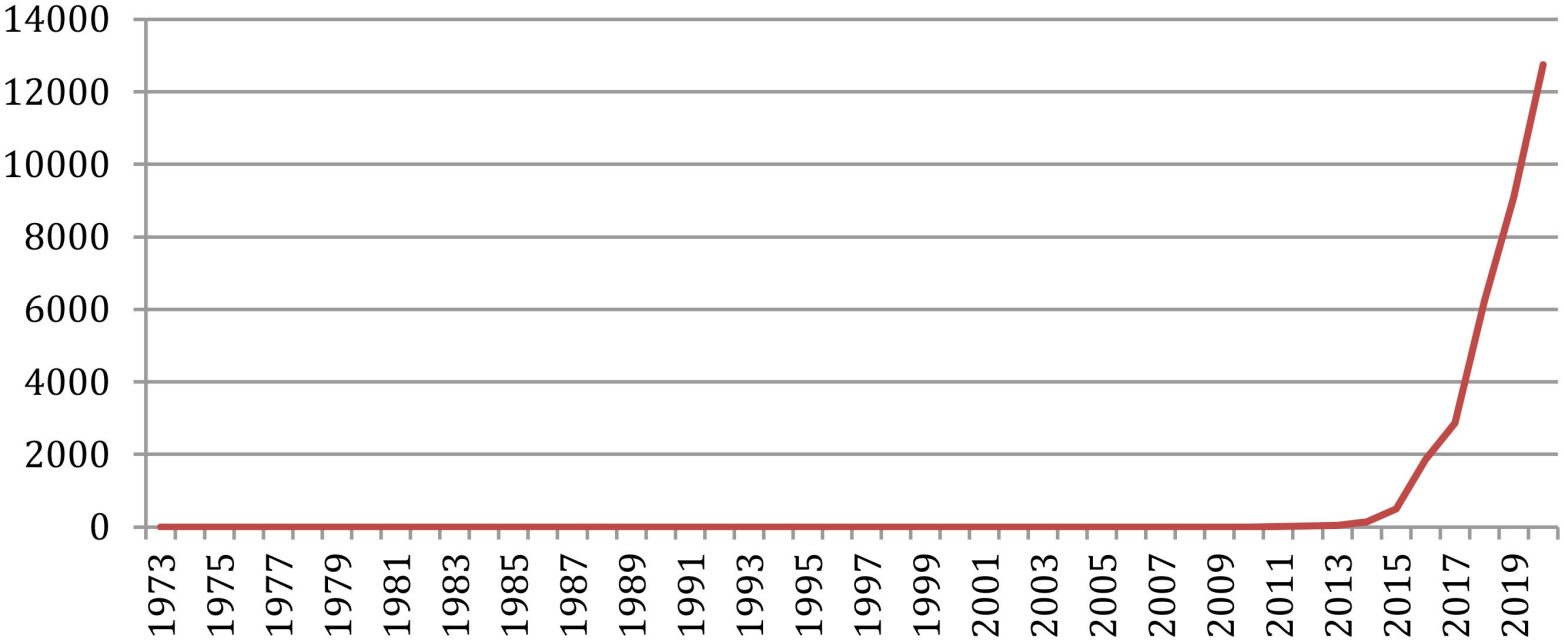
Number of references to arXiv preprints.

Some new sources attract many papers, which resulted in many citations, showing a drastic change in the publications habits. Figure 29 provides the number of references in NLP4NLP+5 papers to *arXiv* preprints, with a huge increase in the recent years (from two references in 2010 to 498 in 2015 and 12,751 in 2020).

**Figure 30:**
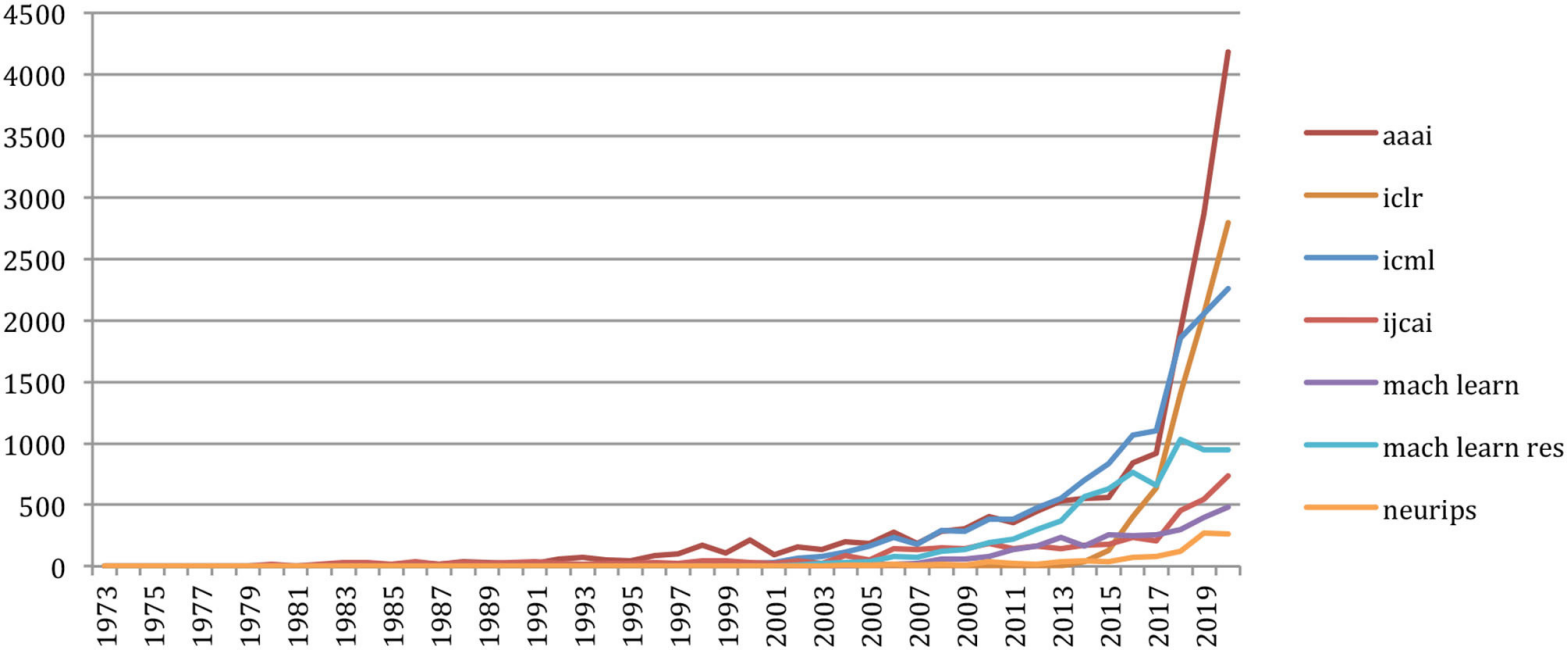
Number of references related to AI, neural networks, and machine-learning sources external to NLP4NLP.

Also, the number of references related to the publications in artificial intelligence, neural networks, and machine learning, such as the conference on Artificial Intelligence of the Association for the Advancement of Artificial Intelligence (aaai), the International Joint Conference on Artificial Intelligence (ijcai), the International Conference on Machine Learning (icml), the International Conference on Learning Representations (iclr), the Neural Information Processing Systems conference (NeurIPS, formerly nips), or the *Machine Learning* and *Machine Learning Research* Journals, greatly increased in the recent years (Figure 30).

##### Google Scholar H-5 Index

As of July 2021, Google Scholar (Table 20) places as we do ACL first in the ranking of the “Computational Linguistics” conferences and journals category^9^ with an h5-index of 157 and an h5-median of 275 within the past 5 years, followed by EMNLP (132), NAACL (105), COLING (64), TACL (59), ELRA LREC (52), SEMEVAL (52), EACL (52), WMT (47), CONLL (43), CSL (34), SIGDIAL (34), *Computational Linguistics* (30), and IJCNLP (30). In the “Signal Processing” category^10^, Google Scholar places IEEE ICASSP (96) first, then ISCA Interspeech (89), IEEE TASLP (60), LREC (53), CSL (34), SIGDIAL (34), and *Speech Communication* (28). This ranking covers the past 5 years and therefore reflects the recent trends compared with our own results, which concern a smaller number of sources but a longer time period.

**Table 20:** Ranking of 28 top sources according to Google Scholar h5-index over the past 5 years (2016–2020)^a^, in comparison with the previous ranking over 2011–2015.

**Rank** ** 2020**	**Previous** ** Rank** ** 2015**	**Name**	**h-5** ** Index**	**h-5** ** Median**	**Previous** ** h-5 index**	**Previous** ** h-5 median**
1	1	Meeting of the Association for Computational Linguistics (ACL)	157	275	65	99
2	2	Conference on Empirical Methods in Natural Language Processing (EMNLP)	132	235	56	81
3	5	Conference of the North American Chapter of the Association for Computational Linguistics: Human Language Technologies (HLT-NAACL)	105	195	48	71
4	3	IEEE International Conference on Acoustics, Speech and Signal Processing (ICASSP)	96	143	54	73
5	6	Conference of the International Speech Communication Association (INTERSPEECH)	89	150	39	70
6	8	International Conference on Computational Linguistics (COLING)	64	103	38	59
7	4	IEEE/ACM Transactions on Audio, Speech, and Language Processing	60	87	51	78
8		Transactions of the Association for Computational Linguistics (TACL)	59	136		
9	7	International Conference on Language Resources and Evaluation (LREC)	53	81	38	64
10	15	International Workshop on Semantic Evaluation (SEMEVAL)	52	93	23	41
10	16	Conference of the European Chapter of the Association for Computational Linguistics (EACL)	52	98	21	34
12	20	Workshop on Machine Translation (WMT)	47	74	18	24
13	13	Conference on Computational Natural Language Learning (CoNLL)	43	77	24	36
14	10	Computer Speech & Language (CSL)	34	49	32	51
14	19	Annual Meeting of the Special Interest Group on Discourse and Dialogue (SIGDIAL)	34	51	18	27
16		IEEE Workshop on Automatic Speech Recognition and Understanding (ASRU)	33	52		
16	18	IEEE Spoken Language Technology Workshop (SLT)	33	58	18	28
18	12	Computational Linguistics (CL)	30	48	31	40
18	17	International Joint Conference on Natural Language Processing (IJCNLP)	30	48	20	27
20	11	Speech Communication	28	49	32	49
21		Workshop on Representation Learning for NLP	27	72		
22		Biomedical Natural Language Processing	26	37		
23		Workshop on Innovative Use of NLP for Building Educational Applications	25	34		
24	14	Language Resources and Evaluation (LRE)	24	36	23	42
24		Odyssey: The Speaker and Language Recognition Workshop	24	45		
24		International Conference on Natural Language Generation (INLG)	24	35		
27		Natural Language Engineering	23	48		
28		IEEE International Conference on Semantic Computing	22	31		

a *According to Google Scholar, “h5-index is the h-index for articles published in the last 5 complete years. It is the largest number h such that h articles published in 2016–2020 have at least h citations each. h5-median for a publication is the median number of citations for the articles that make up its h5-index”*.

Most conferences of the field considerably increased, and some (such as ACL, EMNLP, NAACL, ISCA Interspeech, Semeval, EACL) even more than doubled, their h-index over the past 5 years, whereas journals stayed at about the same level, apart from the *Transactions of the ACL* (*TACL*), which was launched in 2013 and did not appear in the previous ranking. *arXiv* is also not considered here.

### Topics

#### Archive Analysis

Here, our objectives are 2-fold: i) to compute the most frequent terms used in the domain, ii) to study their variation over time. Like the study of citations, our initial input is the textual content of the papers available in a digital format or that had been scanned. It contains a grand total of 380,828,636 words, mostly in English, over 55 years (1965–2020).

#### Terms Frequency and Presence

As depicted in Mariani et al. (2019b), we distinguished SNLP-specific technical terms from common general English ones after syntactic parsing, with the hypothesis that when a sequence of words is *inside* the NLP4NLP+5 corpus and *not inside* the general language profile, the term is specific to the field of SNLP. The 88,752 documents reduce to 81,634 documents when considering only the papers written in English. They include 4,488,521 different terms (unigrams, bigrams, and trigrams) and 34,828,279 term occurrences. The 500 most frequent terms (including their synonyms and variations in upper/lower case, singular/plural number, US/UK difference, abbreviation/expanded form, and the absence/presence of a semantically neutral adjective) in the field of SNLP were computed over the period of 55 years.

We called “*existence*”^11^ the fact that a term exists in a document and “*presence*” the percentage of documents where the term exists. We computed in that way the *occurrences, frequencies, existences*, and *presences* of the terms globally and over time (1965–2020) and also the average number of *occurrences* of the terms in the documents where they appear (Table 21).

**Table 21:** The number of 25 most frequent terms up to 2020 overall, with number of occurrences and existences, frequency and presence, in comparison with 2015 (terms marked in green are those which progressed in frequency).

**Rank**	**Term**	**Variants of all sorts**	**#Occurrences**	**Frequency**	**#Existences**	**Presence**	**Occurrences/** **existences**	**Previous** ** Rank**	**Delta** ** Ranking**
1	Dataset	*Data-set, data-sets, datasets*	240,691	0.00758	24,288	0.28969	9.91	11	10
2	Annotation	*Annotations*	187,175	0.00589	19,942	0.23786	9.39	4	2
3	SR	*ASR, ASRs, Automatic Speech Recognition, Speech Recognition, automatic speech recognition, speech recognition*	179,579	0.00566	25,916	0.30911	6.93	2	−1
4	LM	*LMs, Language Model, Language Models, language model, language models*	164,944	0.00519	19,139	0.22828	8.62	3	−1
5	HMM	*HMMs, Hidden Markov Model, Hidden Markov Models, Hidden Markov model, Hidden Markov models, hidden Markov Model, hidden Markov Models, hidden Markov model, hidden Markov models*	155,335	0.00489	17,131	0.20433	9.07	1	−4
6	Embedding	*Embeddings*	145,844	0.00459	11,804	0.14079	12.36	29	23
7	Classifier	*Classifiers*	143,885	0.00453	18,540	0.22114	7.76	6	−1
8	POS	*POSs, Part Of Speech, Part of Speech, Part-Of-Speech, Part-of-Speech, Parts Of Speech, Parts of Speech, Pos, part of speech, part-of-speech, parts of speech, parts-of-speech*	135,022	0.00425	18,946	0.22598	7.13	5	−3
9	NP	*NPs, noun phrase, noun phrases*	111,726	0.00352	12,139	0.14479	9.20	7	−2
10	Parser	*parsers*	107,678	0.00339	12,071	0.14398	8.92	8	−2
11	Neural network	*ANN, ANNs, Artificial Neural Network, Artificial Neural Networks, NN, NNs, Neural Network, Neural Networks, NeuralNet, NeuralNets, neural net, neural nets, neural networks*	97,039	0.00306	18,724	0.22333	5.18	17	6
12	Metric	*Metrics*	95,056	0.00299	20,451	0.24393	4.65	18	6
13	Segmentation	*Segmentations*	94,888	0.00299	14,033	0.16738	6.76	9	−4
14	SNR	*SNRs, Signal Noise Ratio, Signal Noise Ratios, signal noise ratio, signal noise ratios*	90,820	0.00286	8,517	0.10159	10.66	10	−4
15	MT	*MTs, Machine Translation, Machine Translations, machine translation, machine translations*	88,790	0.0028	13,603	0.16225	6.53	15	0
16	Parsing	*Parsings*	75,189	0.00237	12,551	0.1497	5.99	13	−3
17	DNN	*DNNs, Deep Neural Network, Deep Neural Networks, deep neural network, deep neural networks*	74,921	0.00236	5,740	0.06846	13.05	63	46
18	GMM	*GMMs, Gaussian Mixture Model, Gaussian Mixture Models, Gaussian mixture model, Gaussian mixture models*	74,820	0.00236	8,203	0.09784	9.12	14	−4
19	ngram	*n-gram, n-grams, ngrams*	73,159	0.0023	11,285	0.1346	6.48	21	2
20	Semantic		70,186	0.00221	16,697	0.19915	4.20	12	−8
21	Decoder	*Decoders*	69,385	0.00219	10,274	0.12254	6.75	71	50
22	WER	*WERs, Wer, word error rate, word error rates*	69,297	0.00218	8,547	0.10194	8.11	20	−2
23	LSTM		68,445	0.00216	7,090	0.08457	9.65	145	122
24	SVM	*SVMs, Support Vector Machine, Support Vector Machines, support vector machine, support vector machines*	67,610	0.00213	9,005	0.10741	7.51	19	−5
25	Iteration	*Iterations*	65,686	0.00207	15,372	0.18335	4.27	16	−9

The ranking of the terms may slightly differ according to their frequency or to their presence. The most **frequent** term overall is “*dataset*,” which accounts for 7.6% of the terms and is present in 29% of the papers, whereas the most **present** term is “*Speech Recognition*,” which is present in 31% of the papers while accounting for 5.7% of the terms. The average number of *occurrences* of the terms in the documents where they appear varies a lot (from 4.2 for “*semantic*” to more than 13 for “*Deep Neural Network*” or 12 for “*Embedding*”).

We also compared the ranking with the 2015 one. A total of 17 of the 20 most frequent terms up to 2015 are still present in this list, with few changes. We see a large progress in the terms associated with the neural network and machine-learning approaches [“*dataset,” “embedding,” “neural network,” “DNN (Deep Neural Networks),” “decoder,” and “LSTM (Long Short-Term Memory)”*] and a small decrease for the terms related to previous approaches [“*HMM (Hidden Markov Models)”, “GMM (Gaussian Mixture Models)”, “SVM (Support Vector Machine)”*].

#### Change in Topics

**Figure 31:**
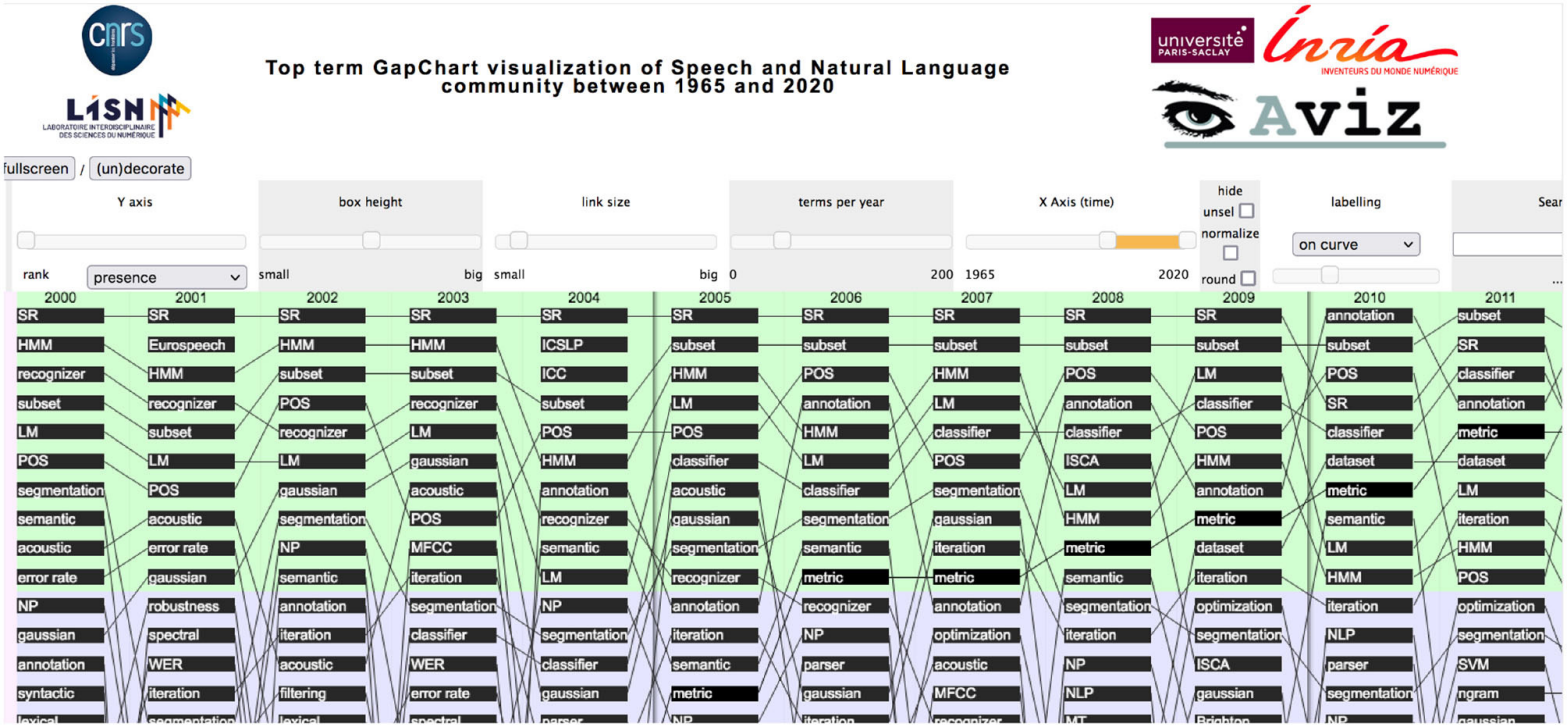
Overview of the GapChart (2000 to 2011) illustrating the parameters. Years appear on the X-axis, and ordered terms (here according to their presence) appear on the Y-axis (10 terms in each color).

The *GapChart* visualization tool^12^ that we developed (Perin et al., 2016) allows us to study the evolution of the terms over the years, based on their frequency or their presence. Figure 31 provides a glimpse of the evolution of topics over time, and we invite the reader to freely access the tool to get a better insight on the evolution of specific terms, such as those illustrated in the following figures.

**Figure 32:**
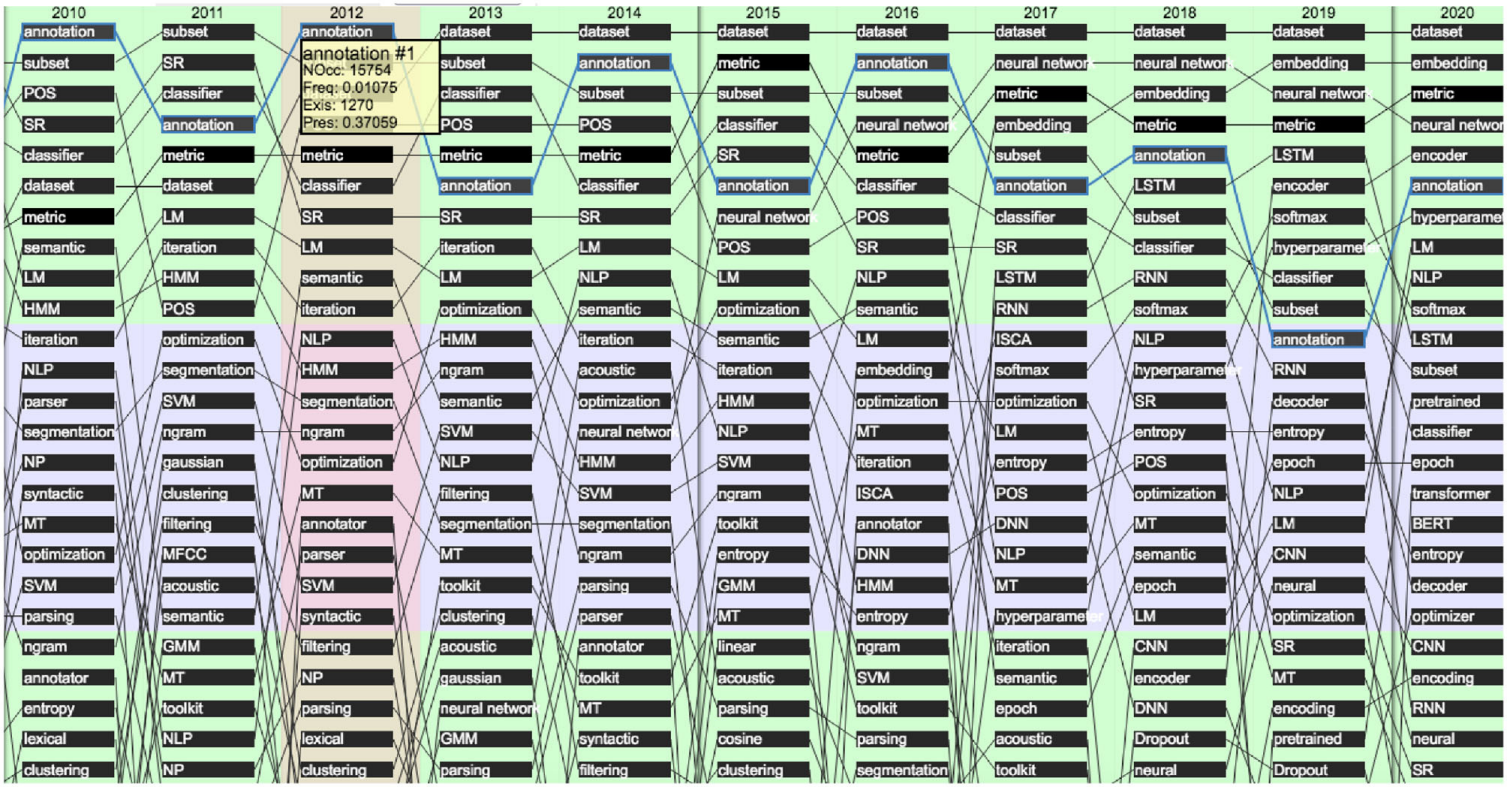
Evolution of the top 25 terms over the past 10 years (2010 to 2020) according to their presence (raw ranking without smoothing).

Figure 32 provides the evolution of the 25 most present terms in the past 10-year period (2010–2020). We see that some terms stay in this list over 10 years, such as “*dataset*,” “*metric*,” or “*annotation*,” while terms related to neural network and machine-learning approaches (such as “*embedding,” “encoder,” “BERT (Bidirectional Encoder Representations from Transformers),” “transformer,” “softmax,” “hyperparameter,” “epoch,” “CNN (Convolutional Neural Networks),” “RNN (Recurrent Neural Networks),” “LSTM,” and “DNN”*) made a large progression.

**Figure 33:**
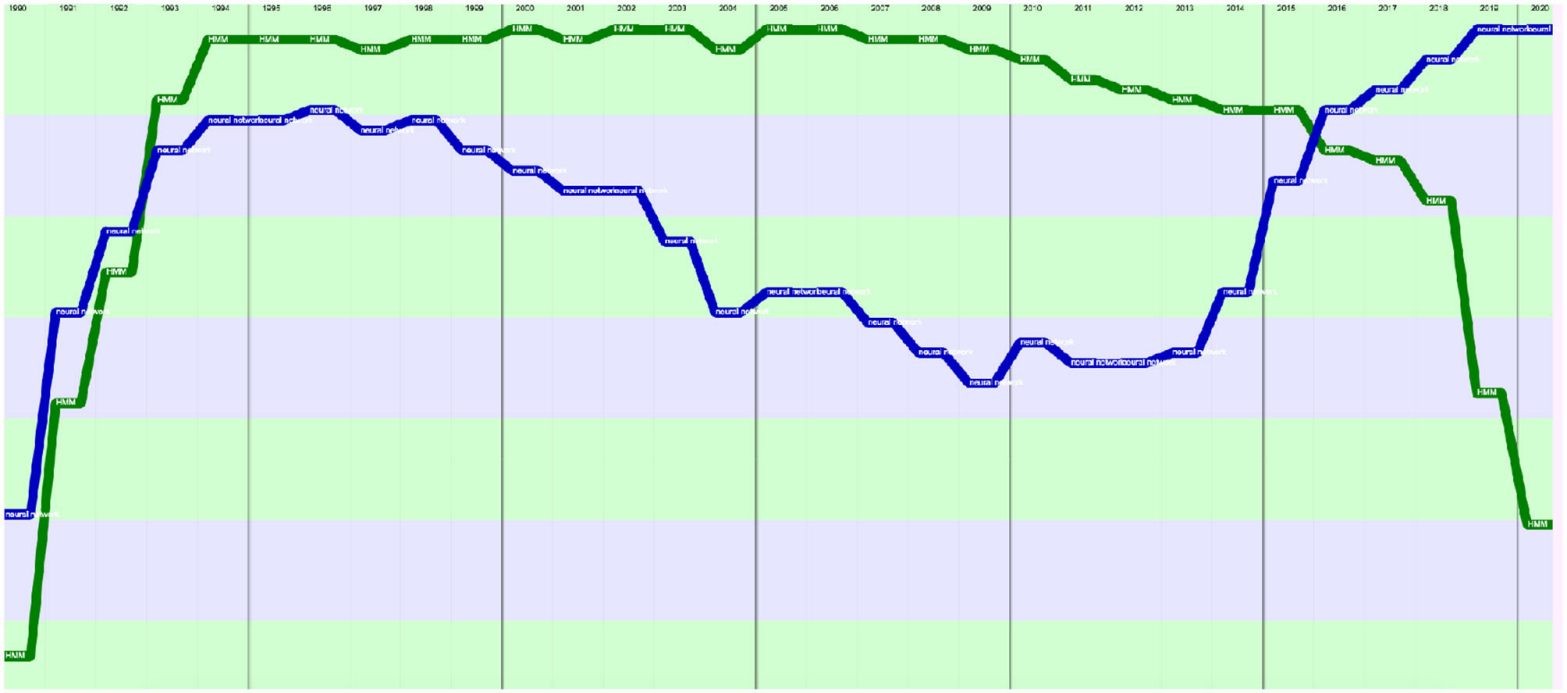
Evolution of the terms “HMM” (in green) and “Neural Network” (in blue) over the past 30 years (1990 to 2020) according to their presence in the papers.

We may also select specific terms. Figure 33 focuses on the terms “*HM*M” and “*Neural Network*” in the 30-year period (1990–2020). It shows the slight preference for “*Neural Network*” up to 1992, then the supremacy of “*HMM*” up to 2015 and the recent burst of “*Neural Networks”* starting in 2013.

**Figure 34:**
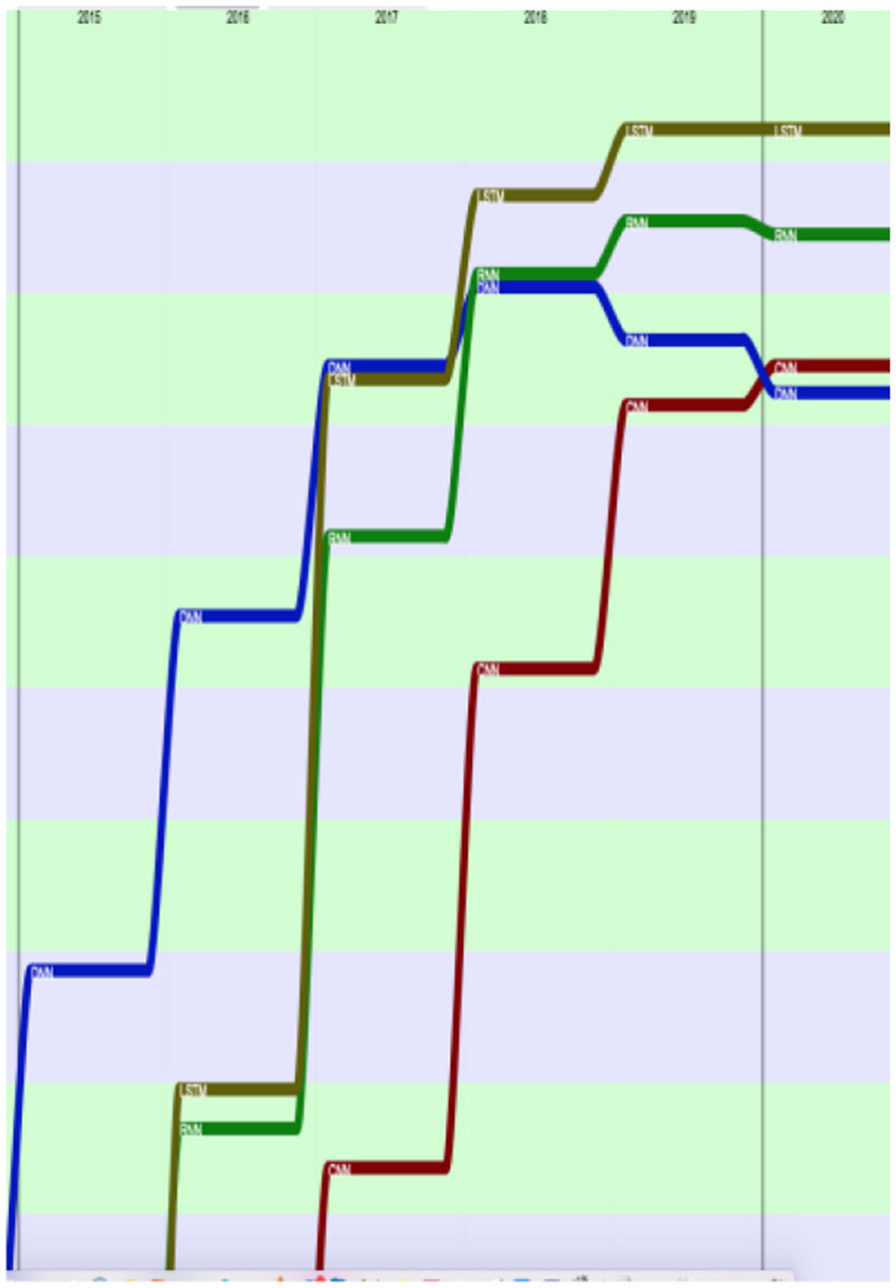
Evolution of the terms “LSTM” (brown), “RNN” (green), “DNN” (blue) and “CNN” (red) over the past 5 years (from the 100th rank in 2015 to the 30th in 2020), according to their presence.

**Figure 35:**
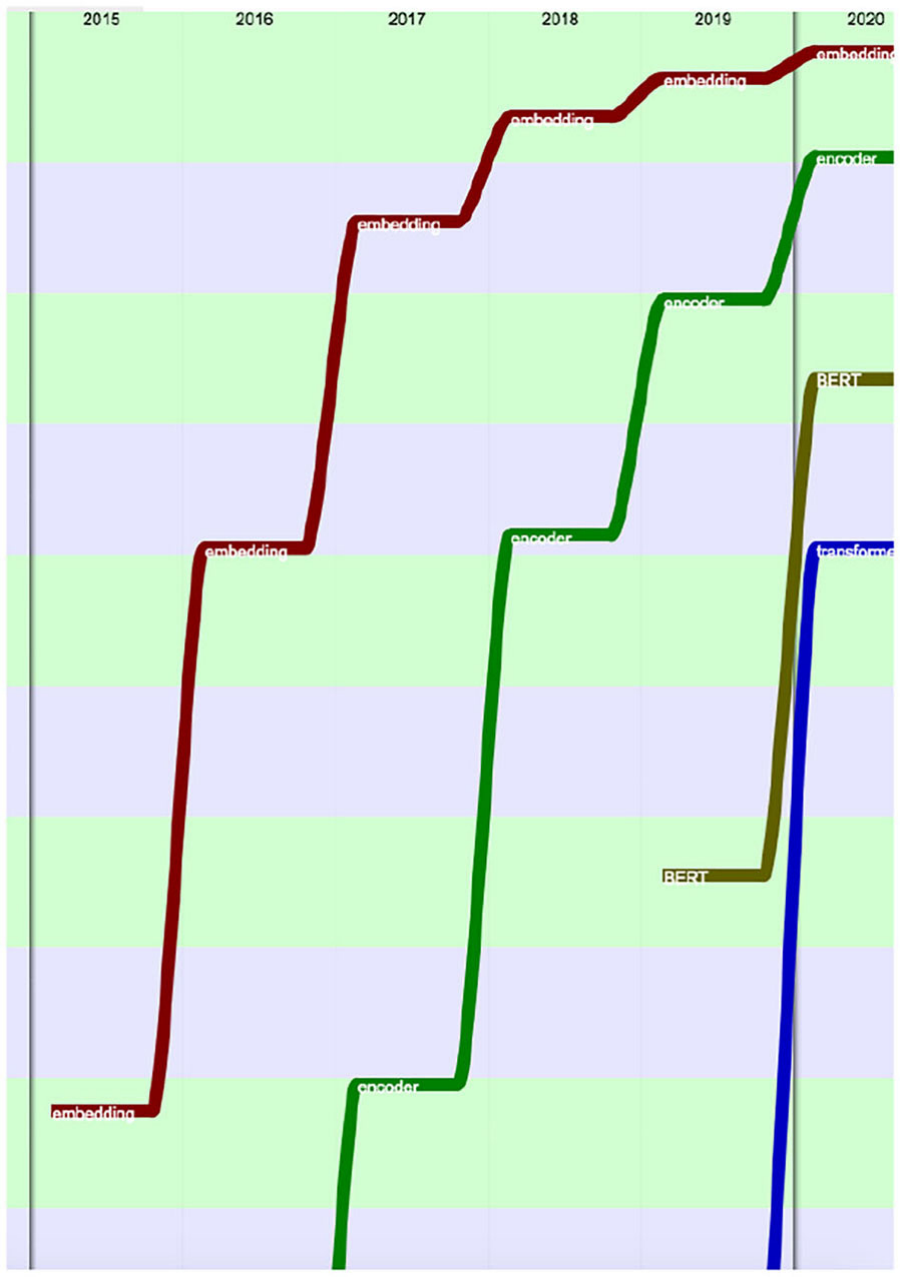
Evolution of the terms “embedding” (red), “encoder” (green), “BERT” (brown) and “transformer” (blue) over the past 5 years (from the 100th rank in 2015 to the 40th in 2020), according to their presence.

**Figure 36:**
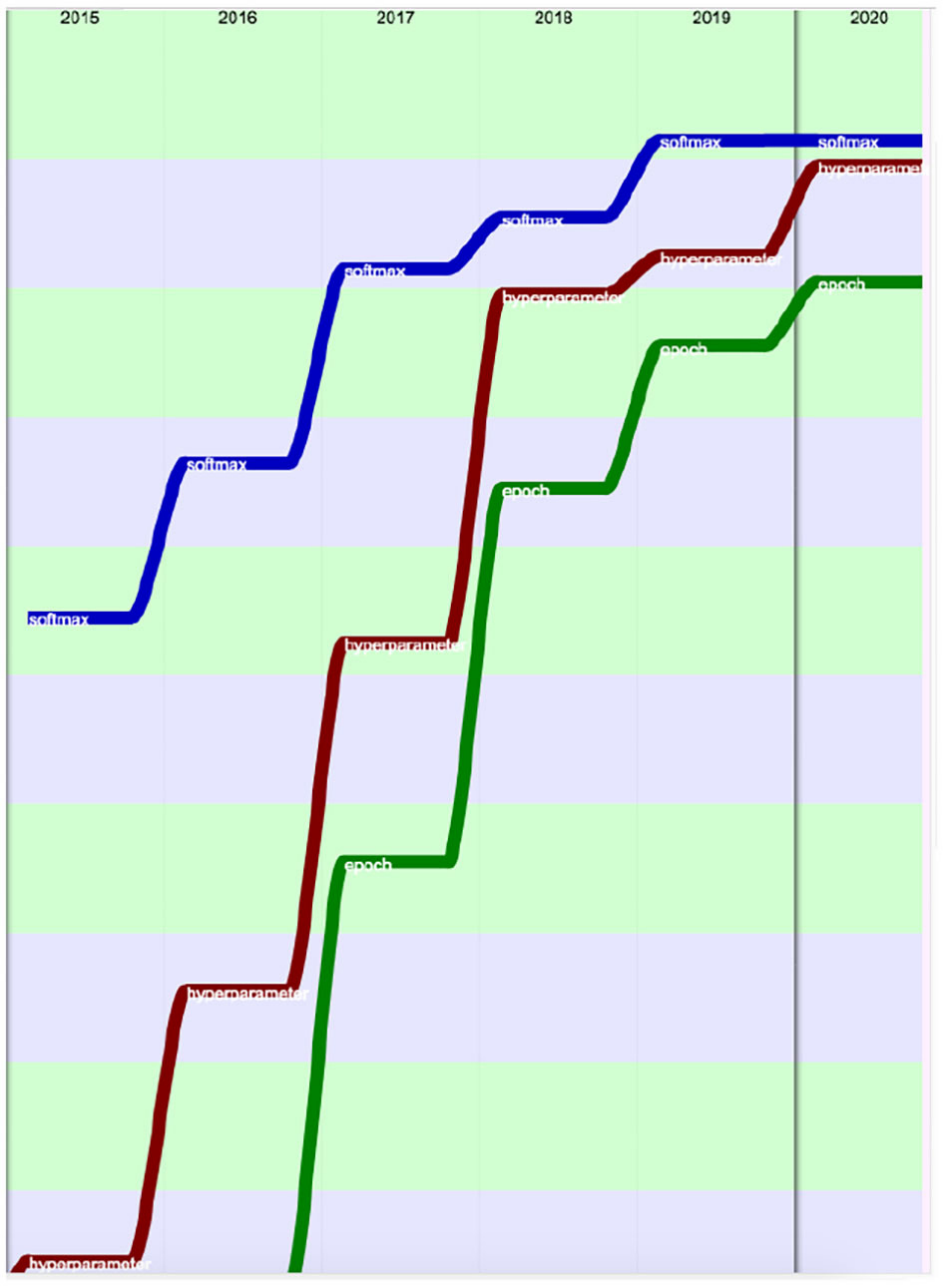
Evolution of the terms “softmax” (blue), “hyperparameter” (red) and “epoch” (green) over the past 5 years (from the 100th rank in 2015 to the 20th in 2020), according to their presence.

The progress on terms related to neural networks and machine learning was especially spectacular over the past 5 years (2015–2020) (Figures 34–36).

#### Tag Clouds for Frequent Terms

**Figure 37:**
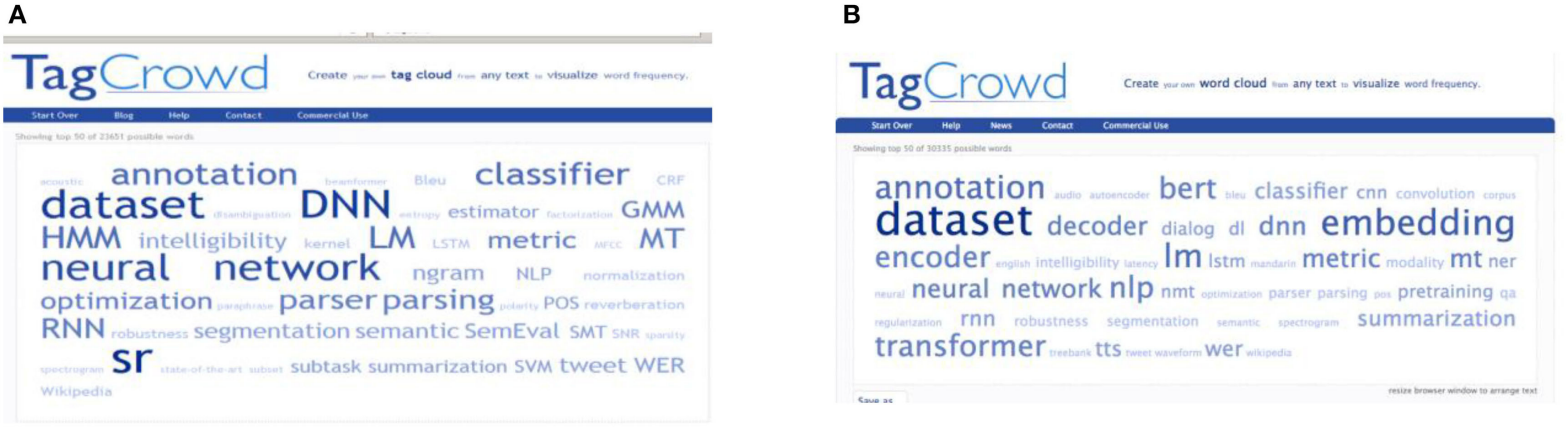
**(A)** Tag Cloud based on the abstracts of 2015. **(B)** Tag Cloud based on the abstracts of 2020.

Tag Clouds provide an estimate of the main terms used on a given year. For this purpose, we use TagCrowd^13^ to generate Tag Clouds. We conducted experiments on full texts and on papers' abstracts and found that papers' abstracts provide a more meaningful analysis as they are more synthetic and contain a larger ratio of technical terms compared with general language. Figures 37A,B provide the Tag Clouds for 2015 and 2020. We clearly see the burst of terms related to machine learning *(“BERT,” “CNN,” “decoder,” “embedding,” “encoder,” “pretraining,” “transformer”*) that were absent in 2015, and the sustainability of “*neural network*,” “a*nnotation*,” “*metric*,” and “*LM*” (“*Language Model”*).

### Research Topic Prediction

#### Machine Learning for Time Series Prediction

We explored the feasibility of predicting the research topics for the coming years based on the past (Francopoulo et al., 2016a). We selected in the time series plug-in of the Weka^14^ machine-learning software package (Witten et al., 2011) the *Gaussian Processes* algorithm with an 18-year window that provided the best results on predicting the term frequency. We then applied this software to the full set of the NLP4NLP+5 corpus, year by year.

Table 22 provides the ranking of the most frequent terms in 2018 and 2019 with their observed frequency, the topic predicted by the selected Weka algorithm for 2020 based on the smallest gap between predictions and observations on the past rankings and the ranking actually observed in 2020. We see that the prediction to have the term within the most frequent top 10 is correct for 8 of them.

**Table 22:** Research topic prediction based on term frequency using the selected Weka algorithm.

**Rank**	**Term**	**Observed**	**Term**	**Observed**	**Term**	**Predicted**	**Term**	**Observed**
		**2018**		**2019**		**2020**		**2020**
1	DATASET	0.019411	Dataset	0.019293	Embedding	0.020756	Dataset	0.020833
2	EMBEDDING	0.012028	Embedding	0.018099	Dataset	0.017509	Embedding	0.015237
3	ANNOTATION	0.008888	Encoder	0.009572	Encoder	0.011884	BERT	0.01076
4	LSTM	0.008571	LSTM	0.008271	BERT	0.008609	Annotation	0.009168
5	DNN	0.006005	Decoder	0.007093	Decoder	0.008261	Encoder	0.009156
6	SR	0.005689	LM	0.006079	Classifier	0.007376	LM	0.006342
7	RNN	0.005585	Metric	0.005929	LM	0.006825	Transformer	0.006299
8	Encoder	0.005373	BERT	0.005745	Metric	0.006738	SR	0.006232
9	Classifier	0.005365	SR	0.005388	LSTM	0.006276	Metric	0.00604
10	Neural network	0.005334	Annotation	0.005326	Transformer	0.004887	LSTM	0.005866

*Predictions are marked in green*.

#### Prediction Reliability

As we published such predictions for the years 2016–2020 in our previous paper, we were eager to verify whether these predictions were correct or not. We thus compared these predictions with the actual figures for these 5 years (Table 23). It appears that the predictions were quite reliable: the number of terms correctly predicted to appear within the 10 top terms varies from 7 to 8 in the first 3 years (2016, 2017, and 2018) that follow the year when the prediction was made (2015) and then decreases to 4 on the 4th year (given that BERT did not exist at the time of the prediction and therefore could not be predicted) and 3 in the 5th year, whereas one term (“dataset”) was correctly predicted with the right ranking for the 5 years. It therefore confirms the assumption we made in our previous papers that predictions seem to be reasonable within a 3-year horizon (unless a major discovery happens in the meanwhile) in this research domain.

**Table 23:** Comparison of the terms predicted in 2015 for the next 5 years (2016–2020) with the actual observations on these years (Predictions are in italics. Terms correctly predicted to appear among the 10 top terms are marked in yellow, term correctly predicted at its rank is marked in green).

***Predicted in 2015***						**Observed in 2020**				
***Prediction 2016***	***Prediction 2017***	***Prediction 2018***	***Prediction 2019***	***Prediction 2020***	**Rank**	**Observation 2016**	**Observation 2017**	**Observation 2018**	**Observation 2019**	**Observation 2020**
*Dataset*	*Dataset*	*Dataset*	*Dataset*	*Dataset*	1	Dataset	Dataset	Dataset	Dataset	Dataset
*DNN*	*DNN*	*DNN*	*DNN*	*DNN*	2	Annotation	Embedding	Embedding	Embedding	Embedding
*Annotation*	*Neural network*	*Neural network*	*Neural network*	*Neural network*	3	DNN	DNN	Annotation	Encoder	BERT
*POS*	*SR*	*RNN*	*RNN*	*RNN*	4	embedding	LSTM	LSTM	LSTM	Annotation
*Neural network*	*Classifier*	*POS*	*Parser*	*Parser*	5	SR	SR	DNN	Decoder	Encoder
*Classifier*	*LM*	*Parser*	*SR*	*SR*	6	LSTM	RNN	SR	LM	LM
*Parser*	*POS*	*Annotation*	*LM*	*Metric*	7	POS	Annotation	RNN	Metric	Transformer
*SR*	*RNN*	*Classifier*	*Classifier*	*POS*	8	Classifier	Neural network	Encoder	BERT	SR
*LM*	*parser*	*SR*	*Metric*	*Parsing*	9	Neural network	Classifier	Classifier	SR	Metric
*HMM*	*HMM*	*Metric*	*POS*	*Classifier*	10	RNN	LM	Neural network	Annotation	LSTM
*Rightly predicted in 10 tops*						*7*	*7*	*8*	*4*	*3*
*Rightly predicted at rank*						*1*	*1*	*1*	*1*	*1*

#### Scientific Paradigms Ruptures

**Figure 38:**
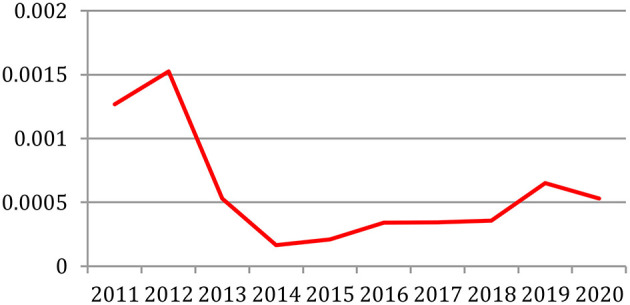
Evolution of the distance between prediction and observation over the years.

As expressed in our previous paper (Mariani et al., 2019b), “*the difference between the prediction and the observation on each year provides a measure of the ‘surprise' between what was expected and what actually occurred. The years where this ‘surprise' is the largest may correspond to epistemological ruptures.”* Figure 38 provides the evolution of this distance between 2011 and 2020, computed as the average absolute value of the difference between prediction and observation for the 200 most frequent terms. It suggests that 2012 was a year of big changes, which then reduced for 2 consecutive years and then slightly evolved since 2014.

**Figure 39:**
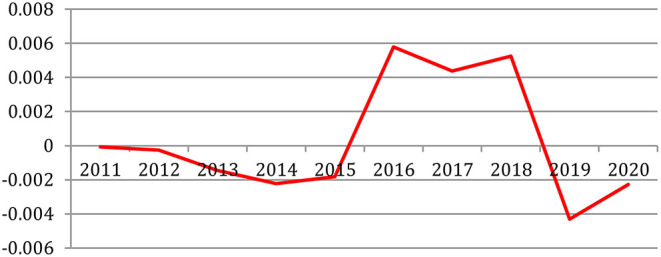
Measure of the expectation of an emerging research topic: Deep Neural Networks (DNN).

The same distance between prediction and observation for a specific topic illustrates the way this term evolved compared with what was expected. Figure 39 shows the evolution of the “*Deep Neural Network” (DNN)* term. It suggests that the popularity (as measured by the frequency of the term) of this approach in the next year was underestimated up to 2015, then overestimated until 2018.

#### Predictions for the Next 5 Years

The predictions for the next 5 years (2021–2025) are provided in Table 24: it is expected that methods based on *machine learning, word embedding*, and *neural networks* will keep on attracting the researchers' attention, with a sustained interest for “*Language Models (LM)*” and a growing interest for “*BERT*” and “*transformer*.”

**Table 24:** Predictions for the next 5 years (2021–2025).

**Rank**	**Observed 2019**	**Observed 2020**	**Prediction 2021**	**Prediction 2022**	**Prediction 2023**	**Prediction 2024**	**Prediction 2025**
1	Dataset	Dataset	Dataset	Dataset	Dataset	Dataset	Dataset
2	Embedding	Embedding	Embedding	Embedding	BERT	BERT	BERT
3	Encoder	BERT	BERT	BERT	Embedding	Embedding	Embedding
4	LSTM	Annotation	Encoder	Annotation	Encoder	Annotation	Transformer
5	Decoder	Encoder	Transformer	Encoder	Transformer	Transformer	Encoder
6	LM	LM	LM	transformer	LM	Encoder	LM
7	Metric	Transformer	Annotation	LM	Annotation	LM	Annotation
8	BERT	SR	Metric	Metric	Metric	Metric	Metric
9	SR	Metric	SR	SR	SR	SR	SR
10	Annotation	LSTM	Decoder	Decoder	Decoder	Annotator	Decoder

*Predictions are marked in green*.

### Innovation

#### New Terms Introduced by the Authors

We studied who introduced new terms which became popular, where and when, to assess the innovative contributions of authors and sources to the advances of the scientific domain (Mariani et al., 2018b). We considered the 81,634 documents written in English and the 61,431 authors who used the 4,488,498 terms contained in these documents. A number of 2,968 of these terms are present in the 22 documents of the first year (1965) that we considered as the starting date for the introduction of new terms, while we found 594,807 of these terms in the 5,313 documents published in 2020. We should stress that the birth of a term is only searched in the 34 NLP4NLP+5 sources and that it may have had a different meaning when it was first introduced.

Table 25 provides the ranked list of the 10 most popular terms according to their presence in 2020 and a comparison with the ranking in 2015. We should notice that 50% of the top 10 terms have changed, with a spectacular increase in the terms related to machine learning and neural networks *(“embedding,” “encoder,” “hyperparameter,”* and “*LSTM”*).

**Table 25:** The number of 10 most present terms in 2020, with variants, date, authors, and publications where they were first introduced, number of occurrences and existences in 2020, number of occurrences, frequency, number of existences and presence in the 55-year archive, with ranking and average number of occurrences of the terms in the documents where they appear, and comparison with the ranking in 2015 (the terms which joined the top 10 are marked in green, while the 5 which went out are marked in orange with their new and former ranking).

**Rank 2020**	**Previous Rank 2015**	**Term**	**Variants of all sorts**	**Event when the term appeared**	**Authors who** ** introduced the term**	**Documents**	**Archive #occurences**	**Archive frequency**	**Archive #existence**	**Archive presence**	**Archive rank** ** occurrences**	**Archive rank** ** existences**	**Archive occurrence/existence ratio**	**#Occurrences of** ** the term in 2020 (by other people than the inventors)**	**#Existences in 2020 (by other people than the inventors)**	**Frequency in 2020**	**Presence in 2020**
1	1	Dataset	*Data-set, data-sets, datasets*	1966	Laurence Urdang	cath1966-3	240,691	0.0076	24,288	0.290	1	2	9.91	59,794	4,313	0.0224	0.795
2	30	Embedding	*Embeddings*	1967	Aravind K. Joshi, Danuta Hiz, Jane J. Robinson, Steven I. Laszlo	C67-1007 C67-1010 C67-1015	145,845	0.0046	11,804	0.141	6	25	12.36	37,346	3,193	0.0140	0.588
3	2	Metric	*Metrics*	1965	A Andreyewsky	C65-1002	95,056	0.0030	20,451	0.244	12	4	4.65	14,352	2,915	0.0054	0.537
4	7	Neural network	*ANN, ANNs, Artificial Neural Network, Artificial Neural Networks, NN, NNs, Neural Network, Neural Networks, NeuralNet, NeuralNets, neural net, neural nets, neural networks*	1972	P J. Brown	cath1972-21	97,031	0.0031	18,716	0.223	11	8	5.18	9,190	2,623	0.0034	0.483
5	>200	Encoder	*Encoders*	1968	Raymond F. Erickson	cath1968-2	62,324	0.0020	6,874	0.082	28	74	9.07	21,444	2,350	0.0080	0.433
6	6	Annotation	*Annotations*	1967	Kenneth Janda, Martin Kay	cath1967-12 cath1967-8	187,175	0.0059	19,942	0.238	2	5	9.39	21,751	2,160	0.0081	0.398
7	67	Hyperparameter	*hyperparam, hyperparameters*	1989	G Demoment	taslp1989-131	22,593	0.0007	7,900	0.094	104	58	2.86	5,232	2,110	0.0020	0.389
8	9	LM	*LMs, Language Model, Language Models, language model, language models*	1965	Sheldon Klein	C65-1014	164,564	0.0052	19,080	0.228	4	6	8.62	14,850	1,977	0.0056	0.364
9	14	NLP	*Natural Language Processing*	1965	Denis M. Manelski, Gilbert K. Krulee	C65-1018	46,094	0.0015	14,243	0.170	40	14	3.24	6,978	1,946	0.0026	0.359
10	146	LSTM		1999	Felix A. Gers, Fred Cummins, Juergen Schmidhuber	e99_93	68,445	0.0022	7,090	0.085	23	70	9.65	13,767	1,934	0.0051	0.356
12	3	Subset	*Sub set, sub sets, sub-set, sub-sets, subsets*	1965	Denis M. Manelski, E. D. Pendergraft, Gilbert K. Krulee, Itiroo Sakai, N. Dale, Wojciech Skalmowski	C65-1006 C65-1018 C65-1021 C65-1025	65,243	0.0021	24,171	0.288	26	29	2.70	5,239	1,913	0.0020	0.353
14	4	Classifier	*Classifiers*	1967	Aravind K. Joshi, Danuta Hiz	C67-1007	143,885	0.0045	18,540	0.221	7	13	7.76	11,125	1,847	0.0042	0.340
24	5	SR	*ASR, ASRs, Automatic Speech Recognition, Speech Recognition, automatic speech recognition, speech recognition*	1965	Denis M. Manelski, Dániel Várga, Gilbert K. Krulee, Makoto Nagao, Toshiyuki Sakai	C65-1018 C65-1022 C65-1029	179,579	0.0056	25,916	0.309	3	1	6.93	14,630	1,423	0.0055	0.262
27	10	Optimization	*Optimization, optimisations, optimizations*	1967	Ellis B. Page	C67-1032	48,412	0.0015	15,221	0.182	36	13	3.18	3,514	1,356	0.0013	0.250
33	8	POS	*POSs, Part Of Speech, Part of Speech, Part-Of-Speech, Part-of-Speech, Parts Of Speech, Parts of Speech, Pos, part of speech, part-of-speech, parts of speech, parts-of-speech*	1965	Denis M. Manelski, Dániel Várga, Gilbert K. Krulee, Makoto Nagao, Toshiyuki Sakai	C65-1018 C65-1022 C65-1029	135,022	0.0042	18,946	0.226	8	14	7.13	7,278	1,158	0.0027	0.213

#### Measuring the Importance of Topics

In our previous paper, we proposed to measure the “*innovation score*” of a term, as the sum of the yearly *presence* of the term since its introduction.

We initially considered the 1,000 most frequent terms over the 55-year period, but given the poor quality and low number of different sources and papers in the first years, we decided to only consider the 45-year period from 1975 to 2020. Table 26 provides the overall ranking of the terms overall and specifically for the NLP and speech processing categories. This list is very similar to the one we computed in 2015 with a slightly different ranking.

**Table 26:** Global ranking of the innovation score of the terms overall and separately for speech and NLP up to 2020.

**Rank**	**Overall**	**NLP**	**Speech**
1	Speech recognition	Semantic	Speech recognition
2	Subset	NP	Spectral
3	Semantic	Syntactic	HMM
4	LM	POS	Filtering
5	Filtering	Parsing	Subset
6	POS	Subset	Acoustics
7	HMM	Parser	Gaussian
8	Iteration	Lexical	Fourier
9	Spectral	Machine translation	Acoustic
10	Metric	Annotation	Linear

**Figure 40:**
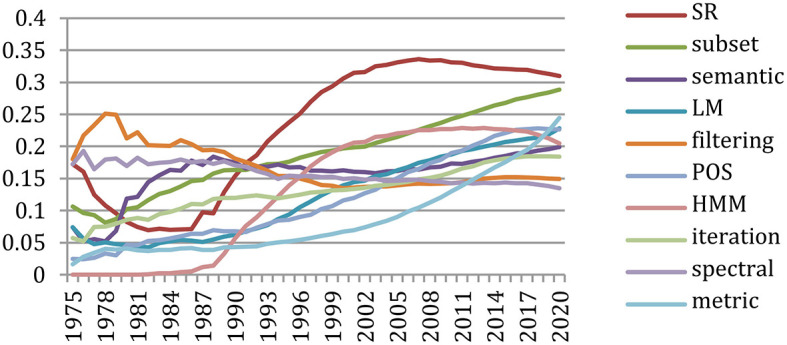
Cumulative presence of the 10 most important terms over time (% of all papers).

We studied the evolution of the *cumulative presence* of the terms over the years (percentage of papers containing a given term **up to** a given year), to check the changes in paradigm while avoiding the noise due to the conference frequency. Figure 40 provides the evolution of the 10 most popular terms according to this measure. Their global ranking over the years corresponds to the order of the terms in the legend of the figure, as it will be the case for all figures in this section Innovation. Percentages are provided either in relation to the total number of papers (qualified as “all papers”), or with the papers related to a specific topic (qualified as “topical papers”).

We see for example that *Speech Recognition* (“*SR*”*)* has been a very popular topic over the years, reaching a presence in close to 35% of the papers published up to 2008, then slightly decreasing.

#### Measuring Authors' Innovation

We computed in a similar way an *innovation score* for each author, illustrating his or her contribution in the introduction and early use of new terms that subsequently became popular, as the sum over the years of the annual presence of the terms in papers published by the author (percentage of papers containing the term and signed by the author on a given year), overall and specifically for the NLP and for the speech processing categories (Table 27). The names in this table are also very similar to those of 2015, with a slightly different ranking.

**Table 27:** Global ranking of the authors overall and separately for speech and NLP up to 2020.

**Rank**	**Overall**	**NLP**	**Speech**
1	Lawrence R. Rabiner	Ralph Grishman	Lawrence R. Rabiner
2	Hermann Ney	Jun'Ichi Tsujii	Shrikanth S. Narayanan
3	Shrikanth S. Narayanan	Kathleen R. Mckeown	John H. L. Hansen
4	John H. L. Hansen	Aravind K. Joshi	Hermann Ney
5	Chin Hui P. Lee	Christopher D. Manning	Chin Hui P. Lee
6	Haizhou Li	Mark A. Johnson	Haizhou Li
7	Mark J. F. Gales	Noah A. Smith	Mark J. F. Gales
8	Mari Ostendorf	Ralph M. Weischedel	Li Deng
9	Li Deng	Eduard H. Hovy	Hervé Bourlard
10	Alex Waibel	Timothy Baldwin	Frank K. Soong

This measure does not place on the forefront uniquely the “inventors” of a new topic, as it is difficult to identify them, given that we only consider a subset of the scientific literature (the NLP4NLP+5 corpus), but it includes the early adopters who published a lot when or just after the topic was initially introduced. Therefore, authors of highly cited papers introducing innovative approaches (such as *Glove* or *BERT* recently) do not fully benefit for their innovation, as many authors immediately adopted, used, and published with these approaches on the same year.

#### Measuring the Innovation in Sources

We also computed an *innovation score* for each source as the sum over the years of the annual presence of the terms in papers published in the source, conference or journal (percentage of papers containing the term which were published in the publication on a given year), overall and specifically for NLP and for Speech Processing (Table 28). The names in this table are also very similar to those of 2015, with a slightly different ranking (progress for EMNLP in NLP and ISCA overall and in Speech Processing).

**Table 28:** Global ranking of the sources overall and separately for Speech and NLP up to 2020.

**Rank**	**Overall**	**NLP**	**Speech**
1	isca	acl	isca
2	taslp	coling	taslp
3	icassps	lrec	icassps
4	acl	emnlp	lrec
5	coling	cath	csal
6	lrec	cl	speechc
7	emnlp	hlt	mts
8	hlt	eacl	lre
9	cl	trec	ltc
10	csal	naacl	acmtslp

#### Measuring the Contribution of Authors and Sources to a Specific Topic

We may also study the contributions of authors or sources to a specific topic, using the cumulative innovation score of authors and sources attached to this topic.

##### Contributions to the Study of “HMM”

**Figure 41:**
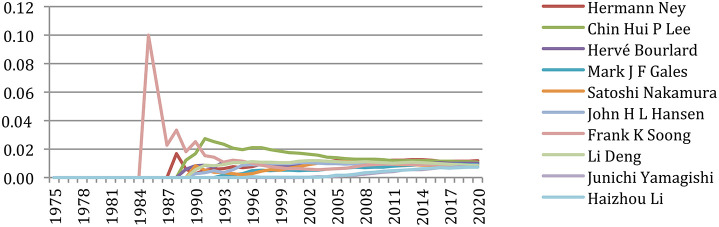
Authors' contributions to “HMM” in speech and NLP (% of topical papers).

Figure 41 provides the cumulative percentage of papers containing the term “*HMM* “published up to a given year by the 10 most contributing authors, ranked according to the innovation measure up to 2020^15^. Compared with 2015, we only observe the appearance of Junishi Yamagishi on innovative HMM-based speech synthesis.

**Figure 42:**
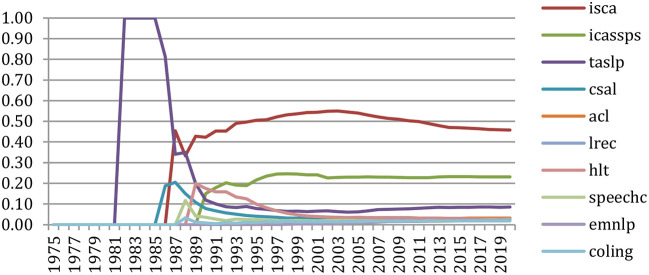
Sources' contributions to “HMM” in speech and NLP (% of topical papers).

We also do not observe much difference regarding the contributions of the various sources to HMMs (Figure 42), with the *IEEE TASLP* as a pioneer in this area since 1982^16^, while *ISCA Conference series* represents 45% and *IEEE-ICASSP* 25% of the papers published on HMM up to 2020 and publications that are placed in both speech and NLP (CSL, HLT, LREC) help to spread the approach from speech processing to NLP as well (ACL, EMNLP).

##### Contributions to the Study of “DNN”

**Figure 43:**
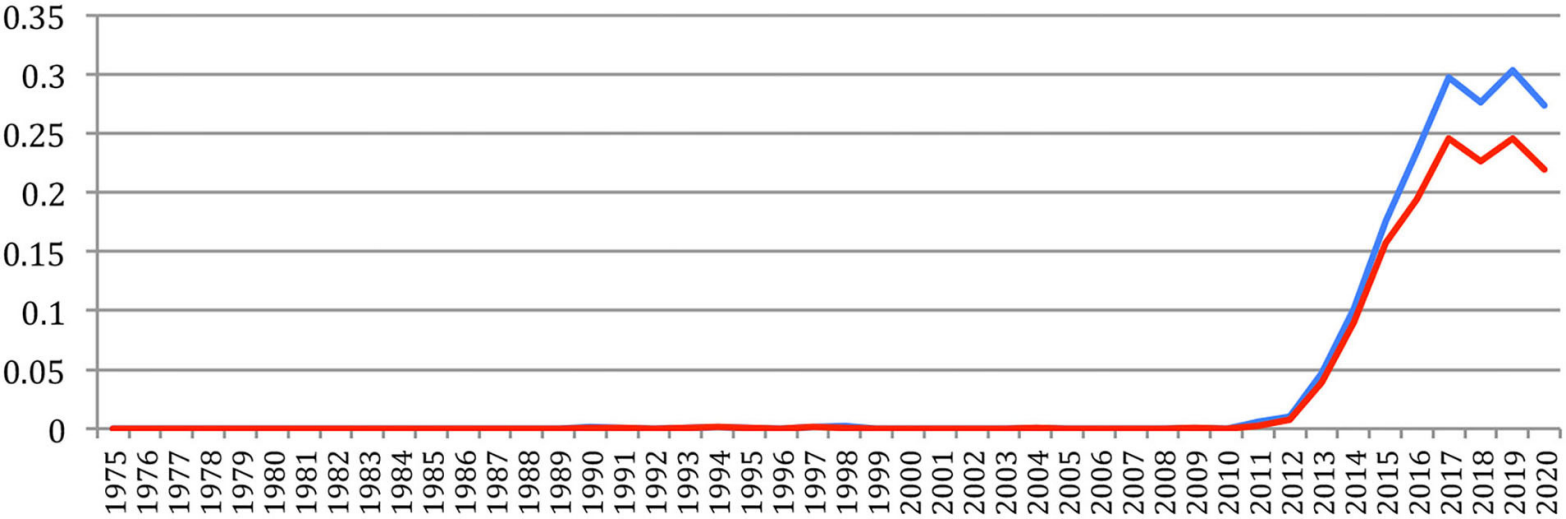
Percentages of authors (blue) and of papers (red) mentioning “DNN” in speech and NLP over the years (% of all papers).

We studied the authors' contributions over the years^17^ to *deep neural networks (“DNNs”*) that recently gained a large audience, in terms of percentage of authors and papers (“*presence*”) mentioning the term (Figure 43).

**Figure 44:**
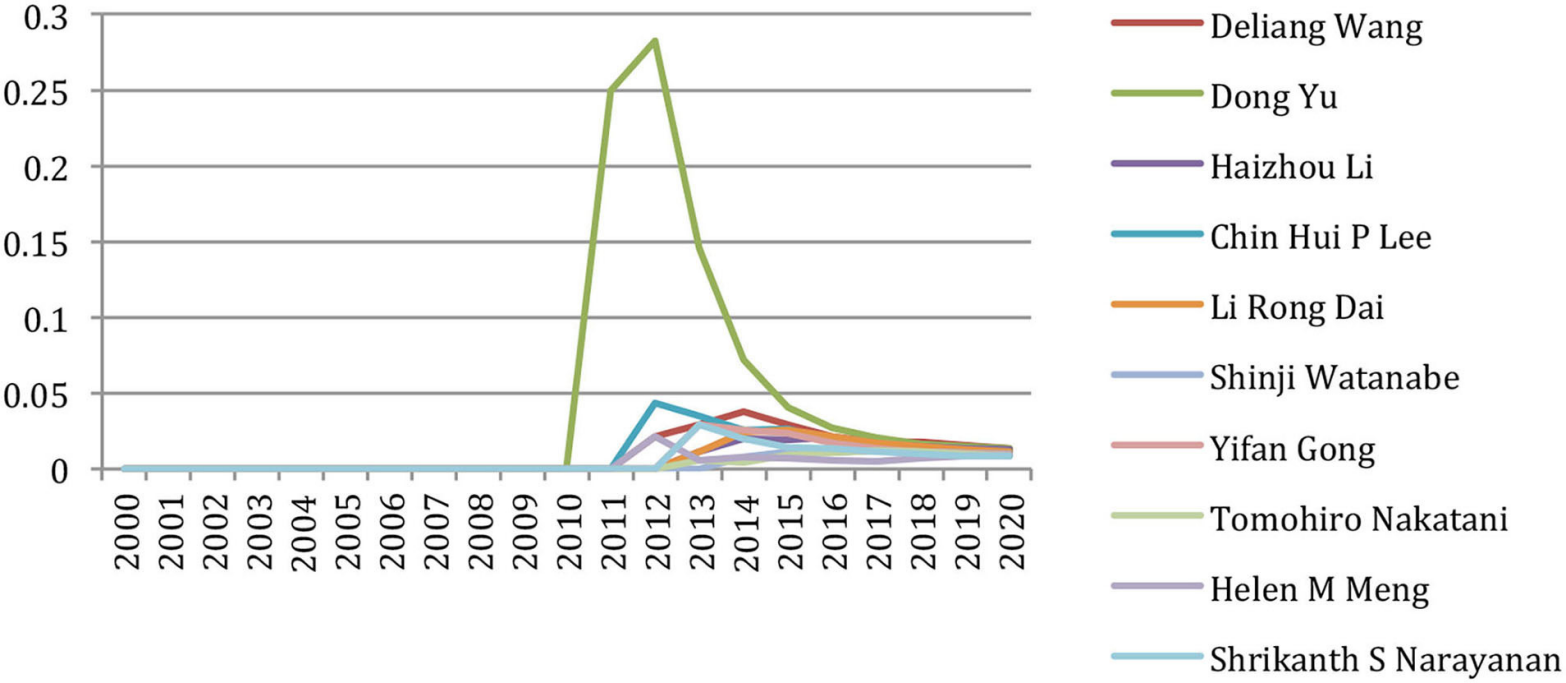
Cumulative authors' contributions to the study of DNN in speech and NLP (% of topical papers).

We notice the important contribution of Asian authors to this topic (Figure 44), with the pioneering contributions of Dong Yu who published about 30% of the papers published on this topic until 2012. Compared with 2015, we notice larger changes than in the case of HMMs, as it is a more changing field, with the appearance of new names (Shinji Watanabe, Tomohiro Nakatani, Helen Meng, and Shri Narayanan).

**Figure 45:**
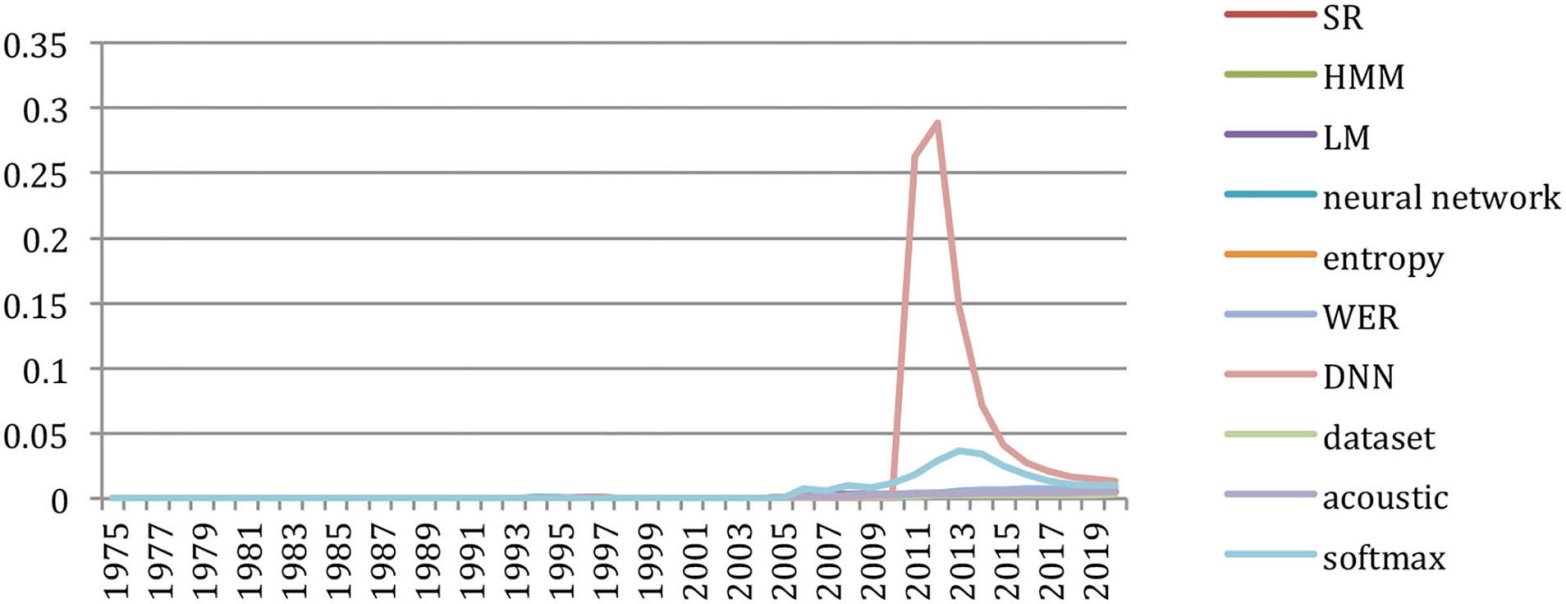
Main contribution areas for Dong Yu (% of topical papers).

We may then also study how an author contribution to a specific topic compares with his/her main contributions to other topics, and how it evolved over the years. Figure 45 illustrates the fact that the contributions of Dong Yu are essentially focused on deep neural networks and second on the Softmax function in the years 2011–2013.

**Figure 46:**
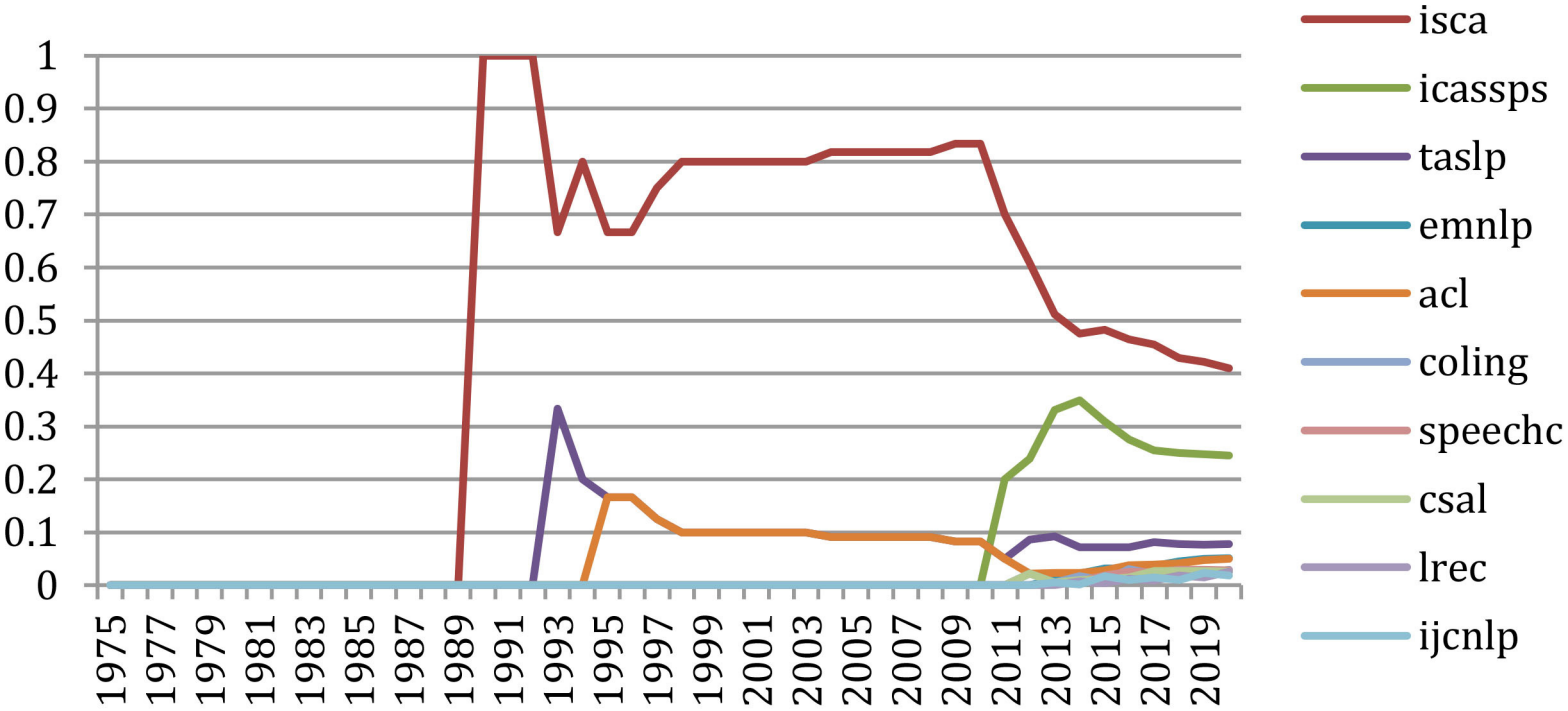
Cumulative sources' contributions to “DNN” in speech processing and NLP (% of topical papers).

Looking at the source contribution to “*DNN*,” we see that it started from the speech community (ISCA Interspeech and IEEE TASLP) and then diffused in the natural language processing community (starting with ACL) (Figure 46).

##### Contributions to the Study of “Embedding”

**Figure 47:**
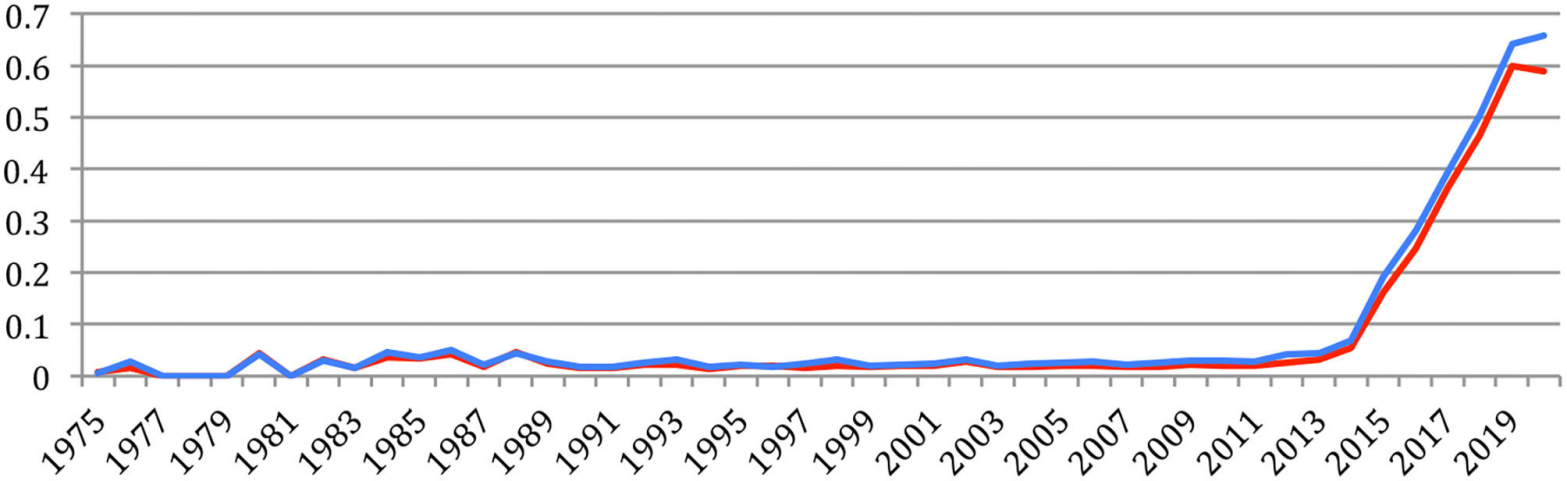
Percentages of authors (blue) and of papers (red) mentioning “Embedding” in speech and NLP (% of all papers).

Similarly, we studied the authors' contributions over the years^18^ to “*Embedding”* which was used for many years from the 70s but gained since 2015 a large audience, in terms of percentage of authors and papers (“*presence*”) mentioning the term (Figure 47).

**Figure 48:**
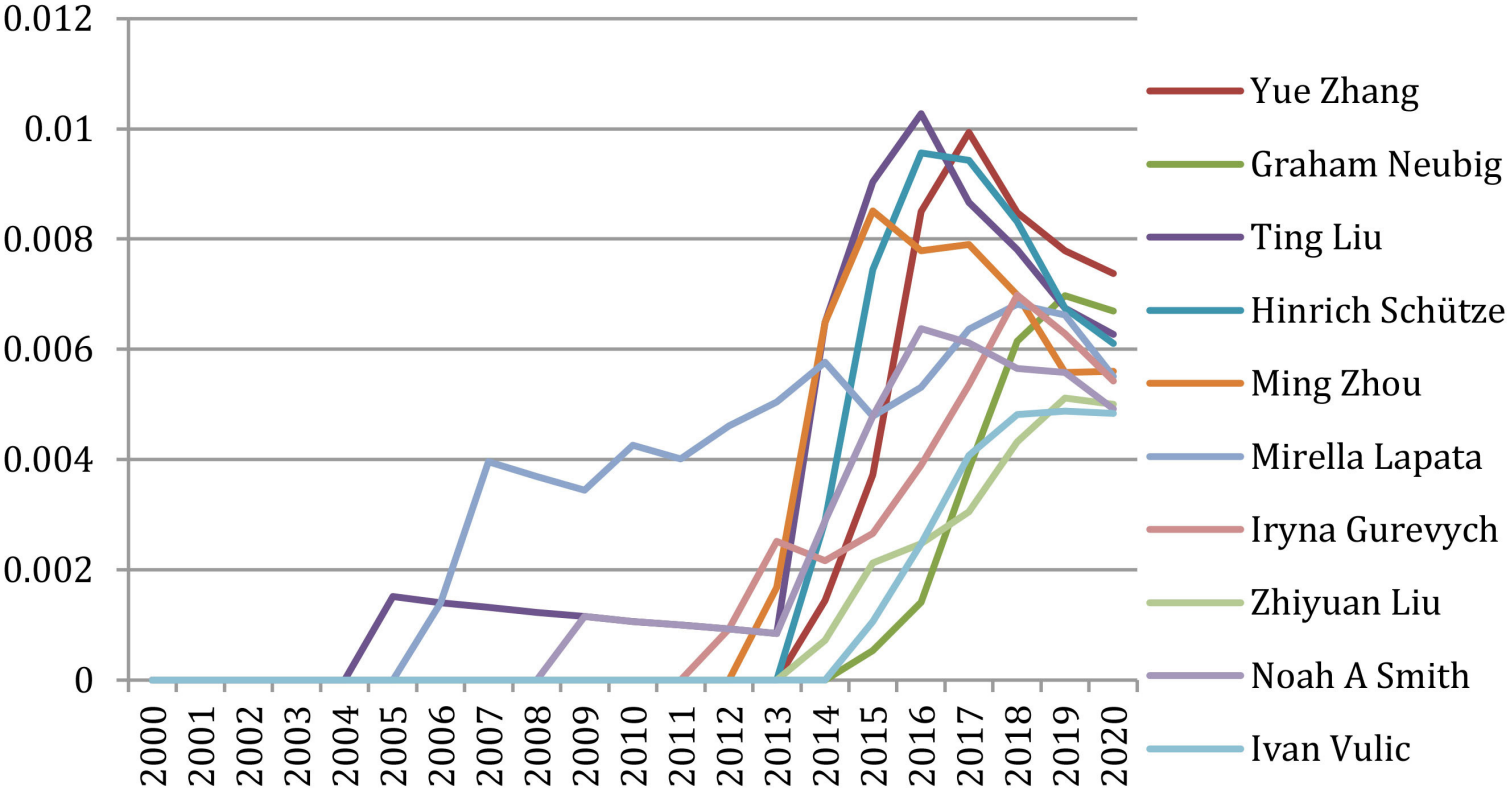
Cumulative authors' contributions to the study of “Embedding” in speech and NLP (% of topical papers).

Figure 48 shows the contribution of authors to the topic of *Embedding*, which both shows the individual contributions and the large increase in the presence of the topic since 2015, as illustrated in Figure 47.

**Figure 49:**
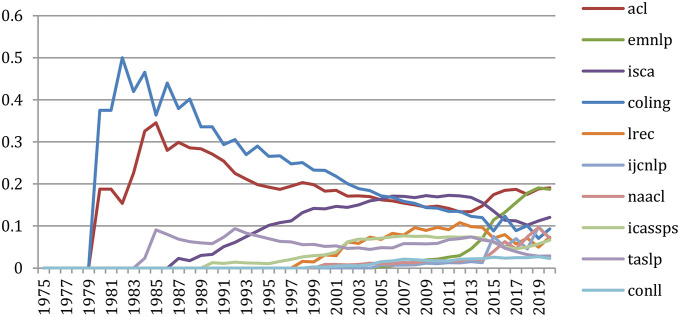
Cumulative sources' contributions to the study of “Embedding” in speech and NLP (% of topical papers).

In this case, the topic was initiated in the natural language processing community (COLING, ACL, IJCNLP) and then diffused in the speech community as well (ISCA Interspeech, ICASSP, TASLP) (Figure 49).

**Figure 50:**
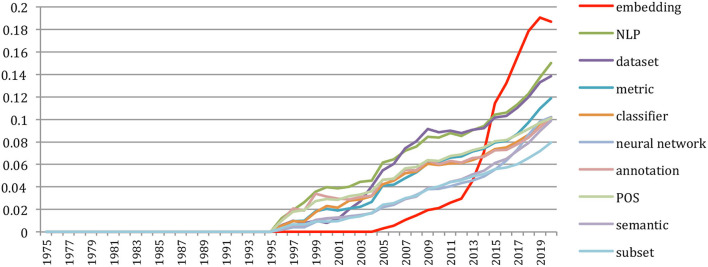
Main contributions of the EMNLP conference series (% of topical papers).

We may then also study how a source contribution to a specific topic compares with its main contributions to other topics, and how it evolved over the years. Figure 50 clearly illustrates the major contribution in the recent years of the EMNLP conference to the research on “*embedding*,” where almost 20% of the papers produced so far on this topic have been published.

### Use of Language Resources

#### The LRE Map

We have conducted an analysis of language resources (LR) as bricks that are used by the researchers to conduct their research investigations and develop their systems (Francopoulo et al., 2016b). We consider here language resources in the broad sense, embracing data (e.g., corpus, lexicons, dictionaries, terminological databases, language models, etc.), tools (e.g., morpho-syntactic taggers, prosodic analyzers, annotation tools, algorithms, software packages, etc.), system evaluation resources (e.g., metrics, training, dry run or test corpus, evaluation packages, etc.), and meta-resources (e.g., best practices, guidelines, norms, standards, etc.) that are mentioned in the LRE Map (Calzolari et al., 2012). This database is produced by the authors of papers at various conferences and workshops of the domain who are invited when submitting their paper to fill in a questionnaire which provides the main characteristics of the language resources produced or used in the research investigations that they report in their paper.

The version of the LRE Map that we used in our previous paper contained information harvested from the authors in 10 conferences from 2010 to 2012, for a total of 4,396 different resources. In the present paper, we use an updated version of the LRE Map containing data harvested in 53 conferences and workshops from 2010 to 2018 (6 more years), for a total of 9,725 resources, that we cleaned up (correct the name of the resources, eliminate the duplicates, regroup the various versions of resources from the same family, etc.). We finally ended up with 5,609 different resources that we searched in the articles of the NLP4NLP+5 corpus.

#### Evolution of the Use of Language Resources

**Figure 51:**
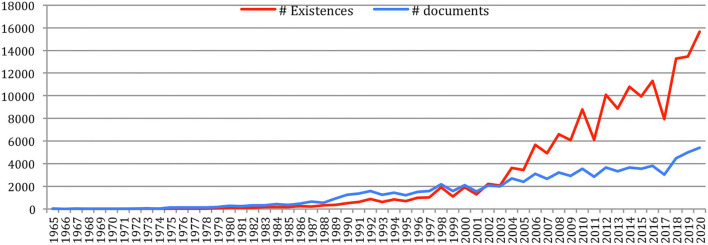
Evolution of the number of papers and of mentions of language resources in papers over the years.

**Figure 52:**
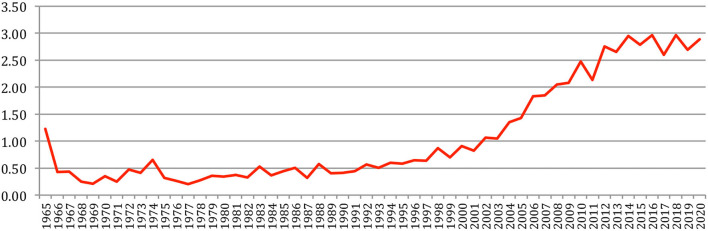
Evolution of the ratio between the number of mentions of Language Resources in papers and the number of papers over the years.

Figure 51 provides the evolution of the number of different resources mentioned in the papers (that we call “*existence*”) compared with the evolution of the number of papers over the years, whereas Figure 52 provides the average number of language resources mentioned in a paper (that we call “*presenc*e”). The corresponding curves cross in 2002, when more than one language resource was mentioned on average in a paper, reflecting the shift from *knowledge-based* approaches to *data-driven* approaches in SNLP research. Since 2015, the number of mentions of language resources largely increased, to attain 16,000 mentions in 2020, whereas the number of papers also greatly increased, and the ratio stays at about 3 language resources mentioned in a paper on qaverage.

Table 29 provides the ranking of language resources according to their “*existence*” (number of papers where they are mentioned), their type (corpus, lexicon, tool, etc.), their number of “*occurrences*” (number of mentions in the papers), the first authors who mentioned them as well as the first publications, and the first and final years when they were mentioned, and a comparison with the 2015 ranking. We see that half of the language resources that were in the previous ranking are still present, “Wikipedia” now being at the first rank, while the other half were previously at a rank higher than 10th (BLEU, MATLAB, AnCora) or were not considered in the LRE Map, being posterior to 2012 (Word2Vec, Glove). We also see that a language resource such as Word2Vec was immediately adopted by many authors on the very same year when it appeared first in a paper of the NLP4NLP+5 corpus.

**Table 29:** Presence of the LRE Map language resources in NLP4NLP+5 articles (2020 compared with 2015).

**Rank 2020**	**Rank 2015**	**Name**	**Type**	**# Existences**	**# Occurences**	**First author**	**First corpora**	**First year**	**Last year**
1	3	Wikipedia	NLPCorpus	6,348	36695	Ana Licuanan, Jinxi Xu, Ralph M. Weischedel	trec	2003	2020
2	1	WordNet	NLPLexicon	5,803	37654	Kenji Sakamoto, Kouichi Yamaguchi, Toshio Akabane, Yoshiji Fujimoto	isca	1990	2020
3	>10	BLEU	NLPSpecification	4,595	42311	Ludovic Lebart	modulad	2001	2020
4	2	Timit	NLPCorpus	3,982	15984	Andrej Ljolje, Benjamin Chigier, David Goodine, David S. Pallett, Erik Urdang, Fileno Alleva, Francine R. Chen, George R. Doddington, Hong C. Leung, Hsiao Wuen Hon, James L. Hieronymus, James R. Glass, Jan Robin Rohlicek, Jeff Shrager, Jeffrey N. Marcus, John Dowding, John F. Pitrelli, John S. Garofolo, Joseph H. Polifroni, Judith R. Spitz, Julia B. Hirschberg, Kai Fu Lee, L. G. Miller, Mari Ostendorf, Mark Liberman, Meiyuh Hwang, Michael D. Riley, Michael S. Phillips, Robert Weide, Stephanie Seneff, Stephen E. Levinson, Vassilios V. Digalakis, Victor W. Zue	hlt, isca, taslp	1989	2020
5	4	Penn Treebank	NLPCorpus	2,786	10,622	Beatrice Santorini, David M. Magerman, Eric Brill, Mitchell P. Marcus	hlt	1990	2020
6	>10	Word2Vec	NLPTool	2,536	8,245	Allan Hanbury, Amir Globerson, Angelina Ivanova, Baobao Chang, Bin Gao, Bing Qin, Bo Tang, Brigitte Grau, Bruno Martins, Bryan Rink, Carina Silberer, Carlos Guestrin, Carmen Banea, Chengqing Zong, Christopher D. Manning, Chuchu Huang, Claire Cardie, Cícero Nogueira Dos Santos, Cícero Nogueira Dos Santos, D. Song, Dakun Zhang, Daniel Zeman, Daniel P. Flickinger, Danqi Chen, David B. Bracewell, Daxiang Dong, Deniz Yuret, Di Chen, Dianhai Yu, Dimitri Kartsaklis, Dmitrijs Milajevs, Duyu Tang, Emanuela Boros, Enhong Chen, Fabin Shi, Fei Tian, Filip Ginter, Furu Wei, Georgiana Dinu, Germán Kruszewski, Guang Chen, Guoxin Cui, Haifeng Wang, Haiyang Wu, Hal Daumé Iii, Hanjun Dai, Heike Adel, Hinrich Schütze, Hu Junfeng, Hua Wu, Idan Szpektor, Ido Dagan, Ignacio Cano, Ion Androutsopoulos, Ivan Titov, Jacob Goldberger, Jan Hajic, Janyce M. Wiebe, Jason Weston, Jeffrey Pennington, Jenna Kanerva, Jiajun Zhang, Jiang Bian, Jiang Guo, Jianlin Feng, Jianwen Zhang, Johan Bos, Johannes Bjerva, John Pavlopoulos, Jordan Boyd Graber, João Filgueiras, João Palotti, Juhani Luotolahti, Jun Zhao, Jun Cheng Guo, Kai Hakala, Kang Liu, Karen Livescu, Kazuma Hashimoto, Keith Adams, Kevin Gimpel, Leonardo Claudino, Li Dong, Liheng Xu, Linda Anderson, Liumingjing Xiao, Maira Gatti, Makoto Miwa, Malvina Nissim, Maosong Sun, Marc Tomlinson, Marco Baroni, Marco Kuhlmann, Marek Rei, Mark Dredze, Matthew Purver, Mehrnoosh Sadrzadeh, Michael Mohler, Miguel B. Almeida, Mikhail Kozhevnikov, Ming Zhou, Mirella Lapata, Mo Yu, Mohit Bansal, Mohit Iyyer, Mu Li, Mário J. Silva, Nan Yang, Navid Rekabsaz, Nianwen Xue, Olivier Ferret, Omer Levy, Oren Melamud, P. Zhang, Peng Hsuan Li, Peter Enns, Philip Resnik, Pontus Stenetorp, Qinlong Wang, Rada F. Mihalcea, Regina Barzilay, Richard Socher, Rob Van Der Goot, Romaric Besançon, Rui Zhang, Sameer Singh, Sanda Maria Harabagiu, Shaoda He, Shizhu He, Shujie Liu, Silvio Amir, Stephan Oepen, Sumit Chopra, Suwisa Kaewphan, Tao Ge, Tao Li, Tatsuya Izuha, Ted Briscoe, Tie Yan Liu, Ting Liu, Travis R. Goodwin, Wanxiang Che, Wei He, Weiran Xu, Wen Ting Wang, Wenzhe Pei, Xiaobo Hao, Xiaoguang Hu, Xiaojun Zou, Xiaolei Liu, Xiaozhao Zhao, Xingxing Zhang, Xinxiong Chen, Xueke Xu, Xueqi Cheng, Yang Liu, Yi Zhang, Yoav Goldberg, Yonatan Belinkov, Yongqiang Chen, Yoon Chul Kim, Yoshimasa Tsuruoka, Yuanyuan Qi, Yuanzhe Zhang, Yuchen Zhang, Yue Liu, Yusuke Miyao, Yuta Tsuboi, Zhen Wang, Zheng Chen, Zhenjun Tang, Zhiqiang Toh, Zhiyuan Liu	acl, coling, conll, eacl, emnlp, lrec, sem, tacl, trec	2014	2020
7	5	Praat	NLPTool	2,123	4,359	Carlos Gussenhoven, Toni C. M. Rietveld	isca	1997	2020
8	>10	MATLAB	NLPTool	1,915	2,842	Demosthenis Stavrinides, Michael D. Zoltowski	taslp	1989	2020
9	>10	GloVe	NLPTool	1,863	6,686	Christopher D. Manning, Jeffrey Pennington, Richard Socher	emnlp	2014	2020
10	>10	AnCora	NLPCorpus	1,694	3,233	Barbara J. Grosz, Jaime G. Carbonell, Mitchell P. Marcus, Ralph M. Weischedel, Raymond Perrault, Robert Wilensky, Wendy G. Lehnert	hlt	1989	2020

Table 30 provides over the past 20 years (2000 to 2020) the number of mentions of the different language resources from the LRE Map together with the number of documents that were published and the list of the 10 most cited language resources on that year. We see in the recent years, the increase of language resources related to machine learning and neural networks (Word2Vec, Weka, Glove, Keras, Seq2Seq, ROBERTa), as well as to the use of metrics (BLEU and now ROUGE) and the appearance of new speech corpora (LibriSpeech, SquaD).

**Table 30:** Ranked top 10 mentioned LRE Map language resources per year (2000–2020).

**Year**	**# Existences**	**# Documents**	**Top10 cited resources**
2000	1,923	2,118	Timit, WordNet, RST, HPSG, Penn Treebank, AnCora, EAGLES, ATIS, LFG, Pronunciation Dictionary
2001	1,283	1,551	WordNet, Timit, Penn Treebank, NOISEX, ATIS, SENSEVAL, HPSG, MATLAB, Maximum Likelihood Linear Regression, TDT
2002	2,200	2,074	WordNet, Timit, Penn Treebank, MATLAB, HPSG, British National Corpus, AnCora, Praat, EAGLES, BAF
2003	2,085	1,991	Timit, WordNet, Penn Treebank, BLEU, BAF, AQUAINT, Pronunciation Dictionary, British National Corpus, HPSG, TAG
2004	3,633	2,695	WordNet, Timit, Penn Treebank, AnCora, Praat, BLEU, British National Corpus, FrameNet, AQUAINT, EuroWordNet
2005	3,453	2,416	WordNet, Timit, BLEU, Penn Treebank, Praat, AQUAINT, GIZA++, MATLAB, Pronunciation Dictionary, ICSI
2006	5,681	3,101	WordNet, Timit, BLEU, Penn Treebank, AnCora, Praat, PropBank, AQUAINT, FrameNet, MATLAB
2007	4,910	2,663	WordNet, BLEU, Timit, Penn Treebank, GIZA++, Praat, MATLAB, SRILM, GALE, Wikipedia
2008	6,582	3,208	WordNet, BLEU, Wikipedia, Timit, Penn Treebank, Praat, AnCora, PropBank, GALE, FrameNet
2009	6,067	2,919	WordNet, BLEU, Wikipedia, Timit, Penn Treebank, Praat, SRILM, GALE, Europarl, GIZA++
2010	8,782	3,547	WordNet, Wikipedia, BLEU, Penn Treebank, Timit, AnCora, GIZA++, MATLAB, Europarl, FrameNet
2011	6,105	2,864	Wikipedia, WordNet, BLEU, Timit, Penn Treebank, GIZA++, MATLAB, SRILM, Praat, Weka
2012	10,097	3,663	Wikipedia, WordNet, BLEU, Timit, Penn Treebank, Praat, Europarl, AnCora, GIZA++, MATLAB
2013	8,874	3,342	Wikipedia, WordNet, BLEU, Timit, Penn Treebank, SRILM, Weka, GIZA++, MATLAB, Praat
2014	10,793	3,663	Wikipedia, WordNet, BLEU, Timit, Penn Treebank, Praat, AnCora, MATLAB, Weka, SRILM
2015	9,932	3,568	Wikipedia, WordNet, Word2Vec, BLEU, Timit, SemEval, MATLAB, Penn Treebank, Praat, Weka
2016	11,303	3,814	Wikipedia, Word2Vec, WordNet, BLEU, Timit, Praat, Penn Treebank, MATLAB, AnCora, Europarl
2017	7,915	3,042	Wikipedia, Word2Vec, BLEU, WordNet, Timit, GloVe, Praat, MATLAB, Penn Treebank, Keras
2018	13,295	4,482	Wikipedia, Word2Vec, BLEU, GloVe, WordNet, Seq2seq, Timit, Penn Treebank, ROUGE, CoreNLP
2019	13,461	5,003	Wikipedia, BLEU, GloVe, Word2Vec, Seq2seq, WordNet, ROUGE, Timit, Penn Treebank, SQuAD
2020	15,652	5,426	Wikipedia, BLEU, GloVe, Word2Vec, Seq2seq, RoBERTa, WordNet, ROUGE, LibriSpeech, SQuAD

#### Language Resource Impact Factor

We proposed to define the “Impact Factor” of a language resource as its existence, in recognition of the importance of the corresponding language resources for conducting research in NLP and of the researchers who provided these useful language resources, similar to the role of a citation index. Table 31 provides the impact factors for the language resources of the “Data,” “Evaluation,” and “Tools” types. We can notice the importance of quality measures (BLEU introduced for machine translation and ROUGE for text summarization) and of the recent burst of machine-learning toolkits (Word2Vec, GloVe, Weka, Seq2seq).

**Table 31:** Language resources impact factor (data, specifications, and tools).

**Data**	**Impact**	**Evaluation**	**Impact**	**Tools**	**Impact**
	**factor**		**factor**		**factor**
Wikipedia	6,348	BLEU	4,595	Word2Vec	2,536
WordNet	5,803	ROUGE	1,335	Praat	2,123
Timit	3,982			MATLAB	1,915
Penn Treebank	2,786			GloVe	1,863
AnCora	1,694			SRILM	1,375
Europarl	1,405			GIZA++	1,314
SemEval	1,257			Weka	1,220
FrameNet	1,202			Seq2seq	1,162
CoNLL	1,091				

### Text Reuse and Plagiarism

Here we studied the reuse of the textual content of NLP4NLP+5 papers in other NLP4NLP+5 papers (Mariani et al., 2016, 2018a).

#### Data

We considered here 88,752 documents published by 66,995 authors from 1965 to 2020, which constitute a large part of the published articles in the field of SNLP, apart from the workshop proceedings and the published books.

The preparation of the textual data is described in the study of Francopoulo et al. (2015b). The overall number of words is roughly 380 MWords. Only the texts in English and French have been retained.

#### Algorithm for Computing Papers Similarity

The detection of “copy & paste” as defined in Appendix 5 is conducted through an algorithm described in the study of Mariani et al. (2019b). The comparison is conducted on a window of seven tokens, using the Jaccard distance and a threshold of 0.04. We therefore consider as potentially reused or plagiarized all couples of articles with a similarity score of 4% or more according to our measure of similarity.

#### Categorization of the Results

Our previous experiments showed that it is necessary to carefully check the results as it may contain false alarms due to the presence of short texts, such as acknowledgments, or of truncated or merged documents due to OCRization for the eldest data. In many cases, the author appears with a different spelling, or references are properly quoted, but with a different wording, a different spelling (e.g., American vs. British English) or an improper reference to the source. We had to manually correct these cases and move the corresponding couples of papers to the right category (from “reuse” or “plagiarism” to “self-reuse” or “self-plagiarism” in the case of papers' authors names, from “plagiarism” to “reuse” in the case of references). This manual correction can be done for the articles placed in the “reuse” and “plagiarism” categories, as they are not very numerous, whereas the detection of the authors' name ensures a good reliability for the “self-reuse” and “self-plagiarism” categories.

For each of the 4 copy and paste categories, we produced the list of couples of “similar” papers according to our criteria, with their similarity score, identification of the common parts, and indication of a similar list of authors or of the same title.

#### Results on NLP4NLP+5

We do not include in this paper the matrices for each of the four categories (self-reuse, self-plagiarism, reuse, and plagiarism) displaying the number of papers that are similar for each couple of the 34 sources (considered as “using sources” and “used sources”) that were presented in our previous paper, as they do not show a large difference with the previous findings. On the 13,068 cases detected using the NLP4NLP+5 corpus, 5,799 (44%) are identified as self-reuse, 6,942 (53%) as self-plagiarism, 152 (1.5%) as reuse, and 175 (1.5%) as plagiarism.

**Figure 53:**
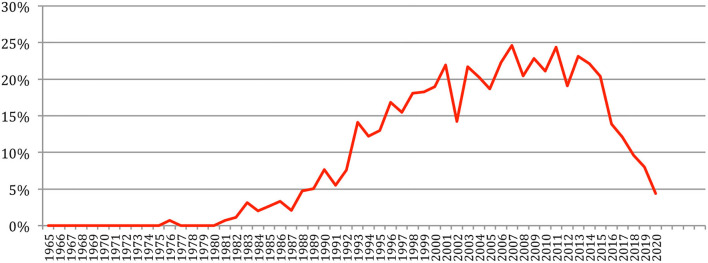
Percentage of papers reusing a part of other papers over the years.

**Figure 54:**
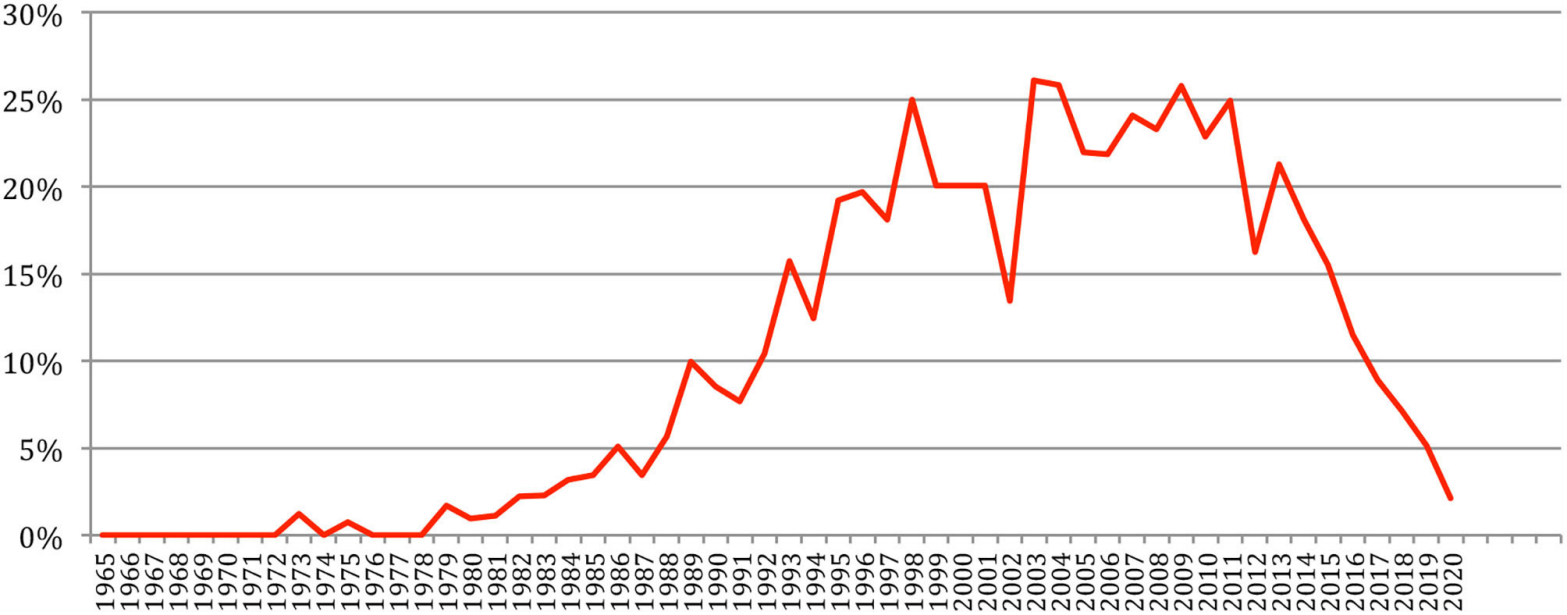
Percentage of papers being reused by other papers over the years.

Figure 53 provides the percentage of papers that are detected as using parts of other papers over the years, whereas Figure 54 provides the percentage of papers that are detected as having been used by other papers over the years, given that they almost entirely correspond to self-reuse and self-plagiarism.

As it clearly shows, self-reuse and self-plagiarism keep being very common: about 25% of papers use parts of previous papers, whereas parts of 25% of papers are used in a new paper. This may also be related to the submission of similar papers at two different conferences on the same year, or to the publication in a journal of a paper previously published in a conference.

Just as noticed in our previous NLP4NLP study, the reuse of papers is done within a short time period (on the same year in 40% of the cases, and within 2 years in 85% of the cases). The reuse of conference papers in journal articles is done with a slightly longer delay (on the same year in 11% of the cases, and within 3 years in 84% of the cases).

#### Plagiarism

We characterized plagiarism by authors using in a paper a large part (more than 4%) of textual content from a paper of other authors without citing the source paper.

In our previous study related to publications up to 2015, 116 cases of possible plagiarisms were detected over 50 years on a total of 63,357 papers (less than 0.4%), which reduced to only one case with a 10% similarity score after a careful manual checking made possible by the small number of detected cases, as described in the study of Mariani et al. (2019b).

From 2015 to 2020, 47 cases of possible plagiarism are detected on a total of 20,649 papers, which also reduce to a single case after a careful manual checking! In addition to the various reasons for the false detection of plagiarism identified in our previous study, we also found out that a paper may be identified as plagiarizing another paper, whereas the authors of that other paper actually plagiarize themselves a former paper of the previous authors!

## Conclusions

When comparing the study contained in this paper with the findings of our two previous papers, we may first consider the results that reinforce on 55 years the conclusions that we made on 50 years.

As already encountered while pursuing the study reported in our previous papers, we appreciated the benefit to have access to a large quantity of publications that are now freely accessible online, while we faced the difficulty of dealing with proprietary data which requires extensive discussions with the publishers to explain the nature of our investigations that necessitates a large collection of papers. It also raises the problem of distributing the data, replicating the results and updating the corpus.

We still struggled with the lack of a consistent and uniform identification of entities (titles, authors names, gender, affiliations, conference and journal names, funding agencies, names of language resources, etc.), which required a tedious manual correction process. This problem would need an international effort to provide unique identifiers to these entities, or else sophisticated disambiguation processes.

We also see that there is less and slow progress in the feminization of the research community.

This study confirmed the possibility to predict some future scientific developments for the next 3 years.

The study of reuse and plagiarism still concludes in the scarcity of real plagiarism cases, a conclusion which, however, needs a careful manual checking in addition to any automatic process, and in the commonness of the reuse of previously published textual content by the same authors which is very widespread and easily understandable, especially when turning a conference paper into a journal article.

While confirming these previous findings, this study also illustrates the tremendous changes in the speech and natural language processing community that happened during the past 5 years.

We first notice a very intense research activity reflected by a huge increase in the number of papers and authors, similar in the single year 2020 to what occurred in the first 25 years of our corpus.

More and more collaborations took place among authors, who formed new clusters. Important changes appear in the ranked list of the most productive, most collaborative, most cited, and best h-indexed authors, with the appearance of many new names, especially of Asian origins, who publish a lot, while many researchers of the pioneering times are now gradually retiring. Also, it seems that slightly more focus is nowadays devoted to NLP compared to speech processing where many breakthroughs have already been achieved in the recent past and which now shares many scientific challenges and many similar approaches in common with NLP.

This is due to the appearance of new paradigms, such as *deep learning, word embedding*, or *unsupervised machine learning*, which immediately attracted a large community of researchers, due to the acceleration in publishing, who increasingly publish in conferences and journals either within NLP4NLP, where we notice a specific increase in activity for the *Transactions of the ACL*, or outside of the NLP4NLP core research area and publications, especially in *arXiv* which now appears as a popular free open-access not-peer-reviewed publication facility.

The use of language resources is also increasing a lot, according to the crucial need of data in machine-learning approaches for developing and improving the quality of systems related to a language, a population or a task, and of proper metrics to measure quality and progress. New language resources of various kinds (dataset, tools, metrics) specifically related to these paradigms became quickly very popular.

Some research domains were initiated or reactivated, such as semantic analysis, sentiment analysis, speech translation, or processing of low-resourced languages.

## Perspectives

We would like to improve the quality of the automatic extraction of information (such as authors' names, references, sources, terms, language resources) to reduce the burden of manual corrections by taking into account the context through novel approaches of disambiguation based on word embedding.

We believe that the raw data that we gathered and the information that we extracted after substantial manual cleaning would provide interesting training and test data for evaluation campaigns (such as automatic name extraction, named entity disambiguation or gender detection).

## Data Availability Statement

The datasets presented in this study can be found in online repositories. The names of the repository/repositories and accession number(s) can be found below: http://www.nlp4nlp.org.

## Author Contributions

JM coordinated the production and writing of the paper. GF produced the corpus and developed the software packages. PP provided specific analyses and regular advices. FV developed the GapChart visualization tool. All authors contributed to the article and approved the submitted version.

## Conflict of Interest

GF was employed by Tagmatica. The remaining authors declare that the research was conducted in the absence of any commercial or financial relationships that could be construed as a potential conflict of interest.

## Publisher's Note

All claims expressed in this article are solely those of the authors and do not necessarily represent those of their affiliated organizations, or those of the publisher, the editors and the reviewers. Any product that may be evaluated in this article, or claim that may be made by its manufacturer, is not guaranteed or endorsed by the publisher.

## Footnotes

^1^ http://www.nlp4nlp.org

^2^ https://arxiv.org/

^3^We call “event” the holding of a conference or the publication of a volume of a journal.

^4^“Authorship” is the signature of a paper by a given author, whatever the number of affiliations he/she may have. If an author published two papers in a conference, it counts for two authorships for this author.

^5^“Epicene” means that the given name is gender ambiguous.

^6^We notice a small discrepancy in the numbers due to the 2015 papers which were not counted in the previous study.

^7^Keynote papers are not taken into account if their content was not included in the conference proceedings.

^8^NLP4NLP sources are slightly different here, as it is no more possible to differentiate papers specifically related to speech in the ICASSP conference.

^9^ http://scholar.google.com/citations?view_op=top_venues&hl=en&vq=eng_computationallinguistics

^10^ https://scholar.google.fr/citations?view_op=top_venues&hl=fr&vq=eng_signalprocessing

^11^Also called “Boolean frequency” or “binary frequency”.

^12^Gapchart: http://vernier.frederic.free.fr/Infovis/rankVis4/.

^13^ www.tagcrowd.com

^14^ www.cs.waikato.ac.nz/ml/weka

^15^Some authors have published earlier on that topic, but don't belong to the 10 most contributive ones on the long run.

^16^We only had access to IEEE ICASSP proceedings since 1990, while *Computer Speech and Language* started in 1986 and the *ISCA Conference Series* in 1987.

^17^The term “DNN” appeared first in a paper published in 1990 (Yamaguchi et al., 1989), but with the meaning of “Dynamic Programming Neural Network”, with a reference to a paper published by Sakoe at ICASSP 1989 (which is not considered in NLP4NLP+5). However, the low presence of the term with this meaning rightly prevents these authors to appear among the main contributors to “*DNN*” in its present meaning.

^18^Here also, the term appeared early in 1967, but with a different meaning and a low presence until the recent years.

## Appendix

### Appendix 1: Acknowledgments

The authors wish to thank the ACL colleagues, Ken Church, Sanjeev Khudanpur, Amjbad Abu Jbara, Dragomir Radev, and Simone Teufel, who helped them in the starting phase, Isabel Trancoso, who gave her ISCA Archive analysis on the use of assessment and corpora, Wolfgang Hess, who produced and provided a 14 GBytes ISCA Archive, Emmanuelle Foxonet who provided a list of authors' given names with the genre, Florian Boudin, who made available the TALN Anthology, Helen van der Stelt and Jolanda Voogd (Springer) who provided the LRE data and Douglas O'Shaughnessy, Denise Hurley, Rebecca Wollman, Casey Schwartz, Dilek Hakkani-Tür, Kathy Jackson, William Colacchio and Shirley Wisk (IEEE) who provided the IEEE ICASSP and TASLP data. They also thank Khalid Choukri, Alexandre Sicard, Nicoletta Calzolari, Riccardo del Gratta, Sara Goggi, and Gabriella Pardelli who provided information about the past LREC conferences and the LRE Map, Victoria Arranz, Ioanna Giannopoulou, Johann Gorlier, Jérémy Leixa, Valérie Mapelli, and Hélène Mazo, who helped in recovering the metadata for LREC 1998, Nancy Ide and Christopher Cieri for their advice and all the editors, organizers, reviewers and authors over those 55 years without whom this analysis could not have been conducted!

### Appendix 2: Apologies

This survey has been made on textual data, which covers a 55-year period, including scanned content. The analysis uses tools that automatically process the content of the scientific papers and may make errors. Therefore, the results should be regarded as reflecting a large margin of error. The authors wish to apologize for any errors the reader may detect, and they will gladly rectify any such errors in future releases of the survey results.

### Appendix 3: Relationship With Other Papers and Reuse of Previous Material

The present paper is the follow up of a series of two papers:

- Mariani et al. (2019a). The NLP4NLP Corpus (I): 50 Years of Publication, Collaboration, and Citation in Speech and Language Processing
- Mariani et al. (2019b). The NLP4NLP Corpus (II): 50 Years of Research in Speech and Language Processing,

which appeared in the same special issue on “Mining Scientific Papers: NLP-enhanced Bibliometrics” of the *Frontiers in Research Metrics and Analytics* journal, edited by Iana Atanassova, Marc Bertin, and Philipp Mayr.

### Appendix 4: Collaboration and Citation Graphs Terminology

Definitions of a *collaboration graph* as a model of social network and on the various structures and measures (*nodes, edges, connected component, giant component, cliques, collaboration distance, collaboration path, diameter, degree* of a node, *clustering coefficient, density, etc.)* are given in Mariani et al. (2018a). We assessed the position of each author in the Collaboration Graph according to various kinds of centrality: *Closeness centrality*, also called *harmonic centrality* (average closeness distance of an author with all other authors belonging to the same connected component), *degree centrality* (number of different co-authors of each author, i.e. the number of edges attached to the corresponding node), *betweenness centrality* [number of paths crossing a node, which reflects the importance of an author as a bridge across different sets of authors (or sub-communities)]. We also defined citing and cited papers and authors *citation graphs* and their structure and measures (*strongly connected components, symmetric strongly connected components, citation distance, diameter, degree* of a node*, average clustering coefficient, density, etc*.) (Mariani et al., 2018a). We studied the four Citing and Cited/Authors and Papers Graphs for each of the 34 sources, either internally to the source or in the context of the NLP4NLP+5 corpus, for the authors:

-the citation by the source authors of the source authors (“*Internal Authors Citations”*),
-the citation by the source authors of NLP4NLP+5 authors (“*Outgoing Global Authors Citations”*),
-the citation by NLP4NLP+5 authors of the source authors (“*Ingoing Global Authors Citations”*).

and for the papers:

-the citation in the source papers of the same source (“*Internal Papers Citation”)*,
-the citation in the source papers of NLP4NLP+5 papers (“*Outgoing Global Papers Citation”)*,
-the citation in NLP4NLP+5 papers of the source papers (“*Ingoing Global Papers Citations”)*.

### Appendix 5: “Copy and Paste” Terminology

As the terminology is fuzzy and contradictory among the scientific literature, we first defined four types of “copy & paste” (Table A1):

- “**self-reuse**” when the source of the copy has an author who belongs to the group of authors of the text of the paste and when the source is cited.
- “**self-plagiarism**” when the source of the copy has an author who belongs to the group of authors of the text of the paste, but when the source is not cited.
- “**reuse**” when the source of the copy has no author in the group of authors of the paste and when the source is cited.
- “**plagiarism**” when the source of the copy has no author in the group of the paste and when the source is not cited.

**Table A1:** Definition of terms.

	**Source is quoted**	**Source is not quoted**
At least one author in both papers	Self-Reuse	Self-Plagiarism
No author in common	Reuse	Plagiarism
